# Supplementary material for: Effectiveness of a ‘Workshop on Decluttering and Organising’ programme for teens and middle-aged adults with difficulty decluttering: a study protocol of an open-label, randomised, parallel-group, superiority trial in Japan
Source: BMJ Open. 2017 Jun 10;7(6):e014687. doi: 10.1136/bmjopen-2016-014687 (PMC5541631; doi:10.1136/bmjopen-2016-014687)
Supplement: Supplementary material 1 [file bmjopen-2016-014687supp001.pdf]

研究参加者への説明文書（12 歳〜14 歳のご本人用）

研究名「整理整頓が苦手な人向けの“片づけ教室”の効果を調べる研究（くじ引きで、片づけ教室を受けるか、受けないかを分けることによって片づけ教室の効果を見る研究)」への参加をお願いしたく、その内容を説明いたします。この研究へ参加するかどうかは、説明を聞いたうえで、あなたが自由に決めることができます。いつでも質問に応じますし、いったん決めた後でも取り消すことができます。ただ、研究を受けていただくことの条件が合わない場合、こちらから参加をお断りする場合もあります。

□研究の目的・意味

この研究の目的は、整理整頓が苦手な 12 歳から 54 歳までの人に「片付け教室+専門家の訪問による片づけ」を行うチーム(教室あり群)と、「専門家の訪問による片づけ」だけを実施するチーム（教室なし群）にくじ引きで分け、調査前、調査開始すぐ、1 か月後、2 カ月後、4 か月後、7 か月後にあなたのお部屋の使いやすさ、片づけが苦手かどうかなどについてアンケートで伺い、「片付け教室」の効果を見ます。どちらの群になるかは、くじ引きで決めるため、あなたがどちらになるかはわかりません。

若い時に整理整頓や片づけがとても苦手な人が年を取った時に、さらにお部屋が散らかっている人が多いことが分かっています。また、片づけや整理整頓の方法は、学校や本で勉強することができますが、片づけ教室の先生から学ぶことで整理整頓が上手になることが考えられます。今回の研究の結果をたくさんの人に伝え、将来、「片付け教室」が、いろいろなところで実施されるようになることによって、片づけが苦手な人のお部屋がきれいになり、生活がしやすくなることを目指します。

□調査を受ける人の集め方と調査の方法

応募をしてくれた人へ、「お部屋の状態を聞くアンケート」を行い、お部屋が散らかっていたり、片づけが苦手な人を調査を受ける人とします。ただし、病気やケガ、障害などで、自分ではお部屋の片づけができない人は、今回の調査は受けられません。調査を受ける人を教室ありチームと教室なしチームにクジで分け、教室ありチームには「4 回の片付け教室+1 回の専門家の訪問による片づけ」を行います。教室なしチームには、「1 回の専門家による片づけ」だけを行い、片づけ教室は行いませんが、調査期間が終わった後に希望する場合は「片づけ教室」を受けることができます。両方のチームについて、あなたと専門家で一緒にあなたのお部屋の片づけを行い、その後のお部屋の片づけの回数や状態、自分のことが好きかどうかなどについてなどのアンケートと、あなたが撮影したお部屋の写真を見て、片づけ状態を判定し、教室ありチームと教室なしチームを比べます。あなたは定期的にアンケートに答えたり、お部屋の写真をメールや郵送で送る必要があります。

この調査に参加することによりあなたのお部屋は整理整頓された状態になりますが、その後、時間がたつにつれ、お部屋がまた、散らかった状態に戻ってしまう可能性はあります。この調査に参加しない場合や途中で参加をやめた場合でも、希望があれば片づけ方法について書かれた本や相談できる場所をお知らせすることができます。

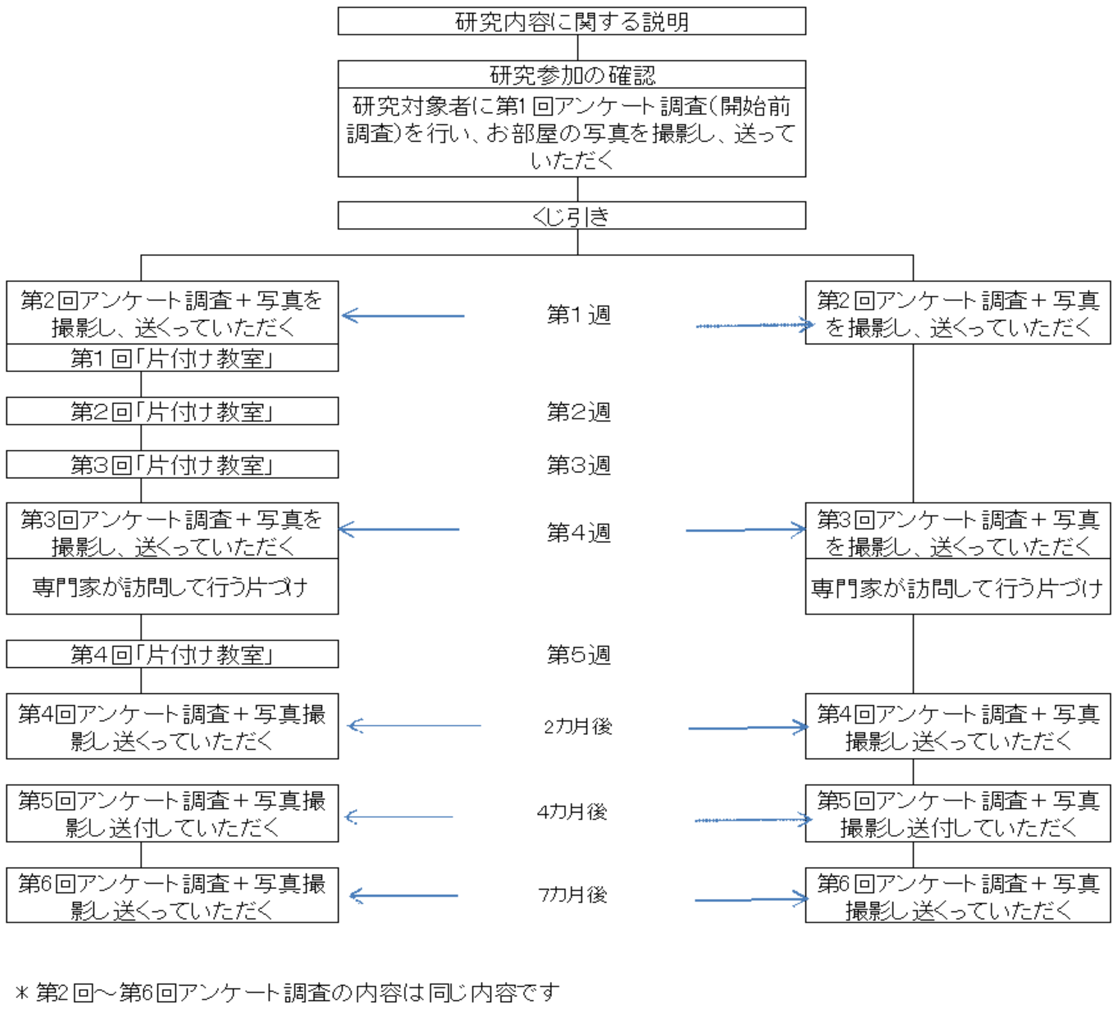

図１　研究全体の流れ

□参加をやめたいとき

　この研究に参加するかどうかは、あなたが自由に決定し、参加しなくても、また、途中で参加をやめても、損をしたり、つらい目にあうことはありません。また、調査が始まった後や、調査が終わった後に、研究への参加をやめたいと思った時は、全員のアンケート結果をまとめる前であれば、あなたのデータを消すことができ、その事により、あなたが損をすることや困ることはありません。

　研究に参加しないと決めた場合や、途中でやめることにした場合でも、研究結果を知りたい場合は、連絡をいただければ、結果についてご報告を致します。

□研究を行う人

　研究を担当する人は以下の通りです。

＜研究代表者＞

＜担当者＞

＜研究協力者＞
＜研究事務局＞

□研究の場所・期間

「片づけ教室」は帝京大学内で行い、専門家の訪問による片づけ・整理整頓作業は、あなたのお部屋であなたと専門家が一緒に行います。

研究開始の許可が下りてから参加する人を募集し、調査開始後 7 か月後までが、あなたにアンケート調査を行う期間です。

□研究で使う資料と情報の取り扱い

調査によって集まったデータは番号が付けられ、誰のものかわからないようになっています。データの扱いは、パソコンに保管してパソコンには他の人が勝手に開けられないよう、パスワードをかけます。紙に書いた情報はデータをパソコンに入力後、お部屋の写真は、判定した乱雑レベルを入力後すぐにシュレッダーで細かく切って内容が分からないようにします。

また、研究代表者は、研究で使用した書類（倫理委員会からのお知らせ文書、申請書・報告書などのコピー、対象者番号が書かれた物、同意書、報告書等のコピー、その他必要な書類）などは保存し、研究発表後 5 年後に捨てます。研究がきちんと行われているかチェックされる場合や、上に書いた研究メンバーが行う将来の研究で使用する時も、データからどの人の内容かが特定されることはありません。

あなたのご自宅に専門業者が訪問する時は、あなたの個人情報が守られるように、文書で専門業者と約束を取り交わします。また、あなたの個人情報が守られるよう、きまりを守るように求めます。

□研究結果の扱い

研究結果は、学会等へ発表することと帝京大学への提出を予定しています。また、片づけ教室のテキストなどは、公表する予定ですが、あなたの個人情報や個人データが広まることはありません。

□研究に必要な費用の出どころ

本研究は平成 26～29 年度「慢性的に片づけられない若年者の実態と効果的介入プログラムの開発」（科学研究費補助金：挑戦的萌芽研究　研究代表者　麻生保子、課題番号 26671045）の研究として行われ、そちらから費用を使用します。

□損・得に関わる行為

この研究に関して、特定の企業や団体から資金はもらっていません。また、この研究によって得をする人と損をする人がいるかどうかについては、「帝京大学板橋キャンパス利益相反管理委員会」で確認してもらっています。また、この調査に参加していただくこと事により、整理整頓方法を学ぶことはできますが、参加費は不要です。アンケート調査や写真を送付していただいた時に、お礼として 200 円～500 円程度の文房具またはクオカード等を差し上げます。

□研究参加の費用等について

「片づけ教室」参加費用や、専門家によるお部屋の片づけ作業の費用、専門家があなたの自宅へ行く時の交通費等をあなたが支払う必要はありません。ただし、お部屋の片づけをする時に、物品をしまう戸棚類をあなたやあなたの保護者が買うことを決めた場合は、あなたや保護者の方が支払うことになります。また、あなたの教室参加の際の交通費およびお部屋の写真撮影やデータを送る時にかかる費用はあなたや保護者の方に支払っていただく事になります。

□研究に参加中にあなたに困ることが起きた際の対応

調査中に体調の悪化やいやな気持ちになった時などは、すぐに調査を中止します。調査中にあなたが何らかのケガや病気になった場合は、研究チームが対応し、大学の保健室へ一緒に行きます。あなたのお部屋の片づけの時に、家具類が壊れたり、あなたやご家族の健康に害があると考えられることがあった場合は、片づけの専門家が入っている損害保険により対応しますが、健康保険での対応となります。担当者の言葉がけや態度、作業内容により、あなたがいやな思いをした場合は、下記の連絡先に連絡してください。

□研究中止の条件

１．あなたが以下の条件となった際には、調査を中止します。

1) あなたや、保護者、一緒に住んでいる方が調査参加をやめると決めた場合
2) 調査の対象者でないことが解った場合
3) あなたの健康や生活にとっての損や不都合が大きく、調査を続けることが難しい場合
4) あなたの体調等により調査を続ける事が良くないと判断された場合
5) あなたがかかっている病気の悪化により調査が難しい場合
6) 調査研究全体が中止された場合
7) その他の理由により研究代表者が調査研究を中止した方が良いと決めた場合

２．調査自体が中止となる条件

1) 調査の安全性や効果がないのではないかと考えられる情報が得られたとき。
2) 調査に参加してくれる人を探す事が難しく予定の人数を探すことが不可能と考えられる時。
3) 予定調査数または予定期間になる前に、調査の目的が達成されたとき。

＊以上の事があった場合は、直ぐに大学内の倫理委員会で話し合い、検討結果をご連絡します。

□質問への対応の仕方・連絡先

研究計画書や研究の方法に関する資料を見たり欲しい場合や質問がある場合は研究代表者に連絡し、資料を見たり、もらったり、質問することができます。連絡先は次の通りです。

説明日：　　　年　　　月　　　日

説明者：
