# Supplementary material for: Effectiveness of a ‘Workshop on Decluttering and Organising’ programme for teens and middle-aged adults with difficulty decluttering: a study protocol of an open-label, randomised, parallel-group, superiority trial in Japan
Source: BMJ Open. 2017 Jun 10;7(6):e014687. doi: 10.1136/bmjopen-2016-014687 (PMC5541631; doi:10.1136/bmjopen-2016-014687)
Supplement: Supplementary material 2 [file bmjopen-2016-014687supp002.pdf]

Explanation Document for Research Participants
(Individuals from 12 years old to 14 years old)

We would like to ask for your participation in, and explain the research on the effect of the "Decluttering and Organizing Class" for people who have difficulty in organizing (separating participants into groups of those with or without class sessions by drawing in order to fairly monitor the effectiveness). You can decide whether you want to join or not after reviewing detailed information on the study. We will address your questions at any time, and you can cancel your participation even after agreeing to participate. If you do not meet our research requirements, we may not be eligible for participation in the study.

□ Purpose and meaning of the research
The purpose of this research is to examine the effect of the "Decluttering and Organizing class" by separating the participants into two groups by drawings: one for those who will attend the Decluttering and Organizing Class as well as an organizing work visit at home (the group with class) and the other for those who conduct an organizing work visit at home for those who have difficulty in organizing between the age of 12 and 54. We will ask questions about the functionality of your room and whether you can organize the room after one month, two months, four months, and seven months. Which group you belong to will be decided by a drawing.

When people have difficulty in organizing at a young age, they tend to be more disorganized when they grow older. Also, while people can learn organizing methods at school or by reading books, they can learn more by working hands on with professionals in organizing classes or workshops. With our research findings, we hope to see more decluttering and organizing classes available for those who are disorganized so that their lives will be a lot easier.

□ How to recruit participants for the research; how the research will be conducted
We will ask the candidates to fill out the "questionnaire about conditions of the rooms", and select people whose rooms are cluttered, or who have difficulty organizing. However, those who are disorganized due to illness, injury, or disabilities cannot participate. The participants will be separated into groups by drawings: one with the class, and one without the class. For the group with the class, 4 "Decluttering & Organizing class sessions and 1 organizing work session at home by organizing professionals" will be offered. For the group without the class, they will have only 1 organizing work session with professionals so that we can measure whether conducting the class is effective or not. In both groups, the organizing professionals will conduct the decluttering & organizing work sessions with the participants. We will ask questions to total of 60 people from both groups, about the number of organizing work sessions conducted, room conditions, and whether you feel positive about yourself or not, and compare the group with the class and the

group without the class. The participants need to answer questionnaires periodically, and send the photos of their rooms via email or a regular mail.

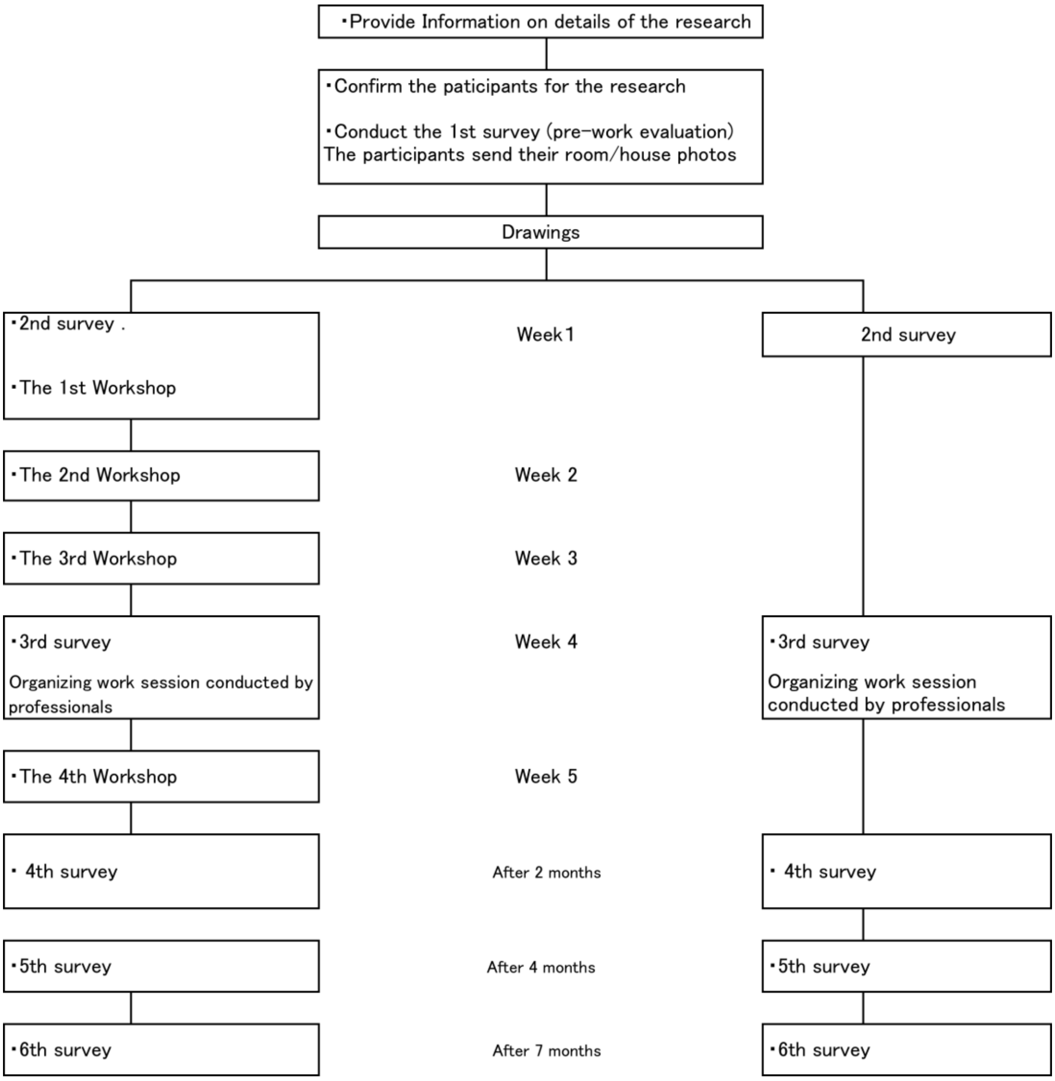

＊The contents of the 2nd through the 6th questionnaire are the same. The participants send their room/house photos when surveyed .

☐ If you want to quit participating

You can freely decide whether you would like to participate in the research or not, and even if you don't participate, you won't lose anything or be at a disadvantage. Also, after the research has started, or has been completed, if you think you want to quit participation, we can delete your data if it is before we compile the findings of the questionnaire. You won't lose anything because of your withdrawal.

In the event that you decide not to participate in the research or chose to withdraw, but you

would like to know the research findings, you can still obtain that information by contacting us.

□ Who will be conducting the research?

<Research representatives>

< Research members>

< Associate Researchers>

<Research administrative>

☐ Location and timeframe of the Research

We will host “the Decluttering & Organizing Class” at Teikyo University. Organizing professionals will visit your home to conduct organizing work sessions with you.

When the research is approved and ready to proceed, we will start recruiting for participants. Seven months after the start will be the end of your (the participants) evaluation period.

☐ Handling of research materials and personal information

Data collected for research will be numbered, so no one will know whose data it is. Then the data will be stored in a computer, which is secured with passwords, so unauthorized people cannot access the computer. Any information on paper will be shredded and destroyed immediately after they are entered in the computer, and photos of your rooms will be shredded after the review.

Also, the research representative chief securely stores documents used for research (announcements from the ethics committee, copies of applications and the reports, documents containing the participants numbers, consents copies of the reports, and other necessary documents), and he or she deletes them after five years. If we check the data in the future to see if the research was done properly or revisit the data for other research projects, we will not be able to identify whose data it is.

When the organizing professionals visit your home, we will sign an agreement with them to not disclose any of your personal information. Additionally, we will ask them to comply with the rules so your personal information is securely protected.

☐ Handling Research Results

We plan to submit and publish the research findings to Teikyo University and academia. Also the

class materials for the Decluttering and Organizing Class will be released; however personal information and individual data will not be shared.

☐ Funding for the research

This research will be done as a part of the study “The reality of young people who are Chronically Disorganized; program development for the effective intervention” (From 2014 to From 2017) (The research subsidy: Challenging *Houga* (Bud flush) Research, Research Representative, Yasuko Aso, Research Number 26671045)

☐ Conflicts of interest

We don’t receive any funds from specific corporate sponsors or organizations for this research. Also, we will have potential conflict of interest of the research screened by the Committee by Itabashi Campus of Teikyo University. You can gain knowledge about organizing by participating in this research, but you don’t have to pay for it. Also, you will not earn any compensation for participating in this research.

☐ Cost for research participation

You are not required to pay for participating in the Decluttering and Organizing Class. You are also not required to pay for the organizing work sessions and any expenses for the vendor’s transportation. However, in the event that the participants (or their parents / guardians) need to purchase closets to store goods when they conduct the organizing work and decide the purchase it, the participants will have to pay for them. It is the participants’ responsibility to pay for any transportation to attend the class, and the cost of mailing their room photos.

☐ How to address adverse events

If your health condition becomes worse or you do not feel well, we will stop the evaluation immediately. If you need to seek medical attention, the medical staff members in the research group will respond accordingly. We will then take you to the nurse’s office at the University. If a piece of furniture or other items at your home are damaged, or you or your family’s health is affected during the organizing work, the liability insurance policy of the vendor will address the issue, but your health insurance is also needed. If you are uncomfortable with words, attitudes, or actions of the workers during the research period, please contact the research members or the following research administrator.

□ Conditions to stop research

１. If you fall under the following conditions, the evaluation will be cancelled.

1) If you or your parents or those who live together with you wish for your withdrawal from

the research

2) If it is determined that you are not eligible for the research
3) If you suffer a large serious setback to your health and lifestyle, and have difficulties continuing the evaluation.
4) If it is decided that continuing the research is not recommended due to health, etc. ,
5) If you develop complications from an illness, and have difficulty continuing the evaluation,
6) If the entire research program is cancelled.
7) If the research representative decides that it is appropriate to stop the examination due to other reasons

２． Conditions under which the entire research program would be cancelled.

1) Significant information about a lack of safety and effectiveness in the research is reported
2) It is decided that recruiting participants for the study is difficult and it interferes with the schedule.
3) The research purpose is achieved before the scheduled date or goal of the evaluation is achieved.

* If any of the above matters are observed, the internal screening committee and ethics screening committee at the university will discuss the matters and the decision will be announced.

□ How to address questions and contact information

If you wish to obtain the materials regarding the research plan or the method, to access them, or to ask questions, please contact the research representative. You will be provided with access, and obtain information or an opportunity to ask questions about the materials.

Please contact:

Date of Explanation ：　　　　Year　　　　Month　　　　Date

Explained by ：______________________

＊ （English version (actually using Japanese version)
