# Supplementary material for: Effectiveness of a ‘Workshop on Decluttering and Organising’ programme for teens and middle-aged adults with difficulty decluttering: a study protocol of an open-label, randomised, parallel-group, superiority trial in Japan
Source: BMJ Open. 2017 Jun 10;7(6):e014687. doi: 10.1136/bmjopen-2016-014687 (PMC5541631; doi:10.1136/bmjopen-2016-014687)
Supplement: Supplementary material 3 [file bmjopen-2016-014687supp003.pdf]

研究参加者への説明文書（15 歳以上のご本人・保護者または同居の方用）

研究名「整理整頓が苦手な若年者を対象とした、無作為割り付けによる “片づけ・整理整頓教室”の効果に関する研究（くじ引きで教室をする群としない群を分け、教室の効果を見る研究)」の参加を依頼したく、その概要を説明いたします。この研究への参加について下記の項目に従い、十分な説明をいたしますので、よく理解された上で、あなた(調査研究対象の方）の自由意思により参加するか否か決めてください。いつでも質問に応じますし、いったん決めた後でも取り消すこともできます。この際に不利益はありません。ただ、条件が合わない場合、こちらの方から参加をお断りする場合もあります。

□研究の目的・意義

本研究の目的は、整理整頓が苦手で、かつ、アンケート調査により居室状態が比較的乱雑であると判定された方を対象とし、くじ引きにより「片付け・整理整頓教室+家庭訪問による整理整頓作業」を実施する群(教室実施群)と、「家庭訪問による整理整頓作業」のみを実施する群（教室なし群）に分けて、教室の効果を見ることを目的としています。尚、研究対象者の方がどちらの群になるかを選択することはできません。両群の比較は、調査前、調査直後、1 か月後、2 カ月後*、4 か月後、7 か月後に対象者の居室状態と生活の質、整理整頓への知識や苦手意識、自尊感情等について評価し、「片付け・整理整頓教室」の効果を比較します。極端に居室状態が乱雑な人は、加齢により体力の衰えとともに、整理整頓能力が衰えることが分かっています。昨今、いわゆる「片付け本」に沿って、自分で整理整頓方法を学ぶ人も増えていますが、片づけ支援教室による学びと、効果的な整理整頓作業は、片付けが苦手な人にとっては有効であることも、既存の研究で公表されています。本調査結果を広く公表し、将来、効果的な「片付け・整理整頓教室」が、広く行政や片付け支援業者によって実施されるようになることによって、整理整頓が苦手な方の生涯にわたる生活の質の向上に寄与することを目指します。

□研究の対象と方法

公募等による応募者へ質問紙により事前調査を行い、研究代表者が以前行った調査結果（2 大学 450 人の調査結果）の平均点より高かった人のうち、自力で整理整頓作業を行うことができる人を今回調査の対象者とします。対象者を教室実施群と教室なし群にくじ引きで分け、教室実施群には「4 回の片付け・整理整頓教室+1 回の家庭訪問による整理整頓作業」を行います。教室なし群には、「1 回の家庭訪問による整理整頓作業」のみを行い、教室の効果を比較します。教室なし群には「片づけ・整理整頓教室」は行いませんが、調査終了後、希望があれば「片づけ・整理整頓教室」を実施します。両群の比較に当たっては、写真による居室状況の把握の他、居室状況に関する調査票や、自尊感情尺度得点、整理整頓への知識や苦手意識、直近 1 週間の整理整頓状況（整理整頓の頻度、居宅内環境活用状況)、直近 1 週間の自宅への来訪者数等を伺います（以下、これら一連の調査を「アンケート調査」とします)。また、調査開始前に同居者数や整理整頓の頻度、居室の数と広さ他、整理整頓状況に関わる質問や、あなた(調査研究対象の方）の状況がわかる、年齢、学歴、勤務形態等に係る研究開始前調査を行います。調査実施に当たり、定期的に居宅状況に関するアンケート調査への回答やご自宅の写真をメールか郵送で送付していただく必要があります。(図 1)本研究参加による、専門家があなたのご自宅を整理整頓することにより一時的にあなたのお部屋は整理整頓されますが、時間の経過とともに、再度、お部屋が乱雑状態になる可能性はあります。本研究に参加しない場合や途中で参加を取りやめ

た場合でも、ご希望があれば片づけ方法について書かれている資料や、状態に応じた相談機関をご紹介することができます。

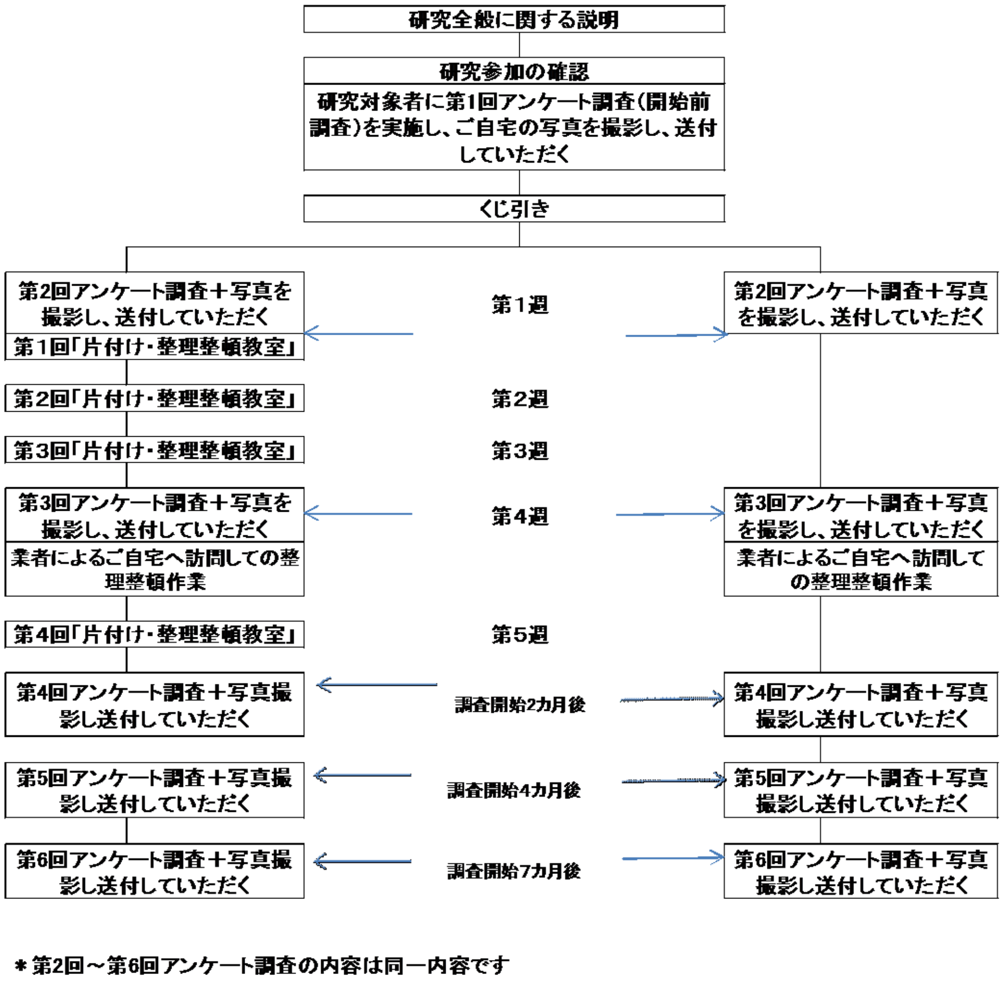

図１　研究全般の流れ図

□研究への自由意思参加・同意取消しの自由

本研究への参加は全くの自由意思で決定し、調査への参加の拒否をしても不利益を被ることはありません。また、調査の途中で参加を中止する場合も、不利益を被ることは一切ありません。調査が行われた後においても同意を取り消すことができます。調査開始または終了後に同意を取り消した場合、データが統計解析前であれば、データの削除を行います。また、同意を取り消すことにより不利益は生じません。研究への参加に同意されなかった場合または途中で参加を取りやめた場合であっても、希望があれば研究結果をご報告いたします。

□研究の責任者・組織

本研究は帝京大学を主とした多施設共同研究です。研究組織は以下の通りです。

＜研究代表者＞
＜研究担当者＞
＜連携研究者＞
＜研究事務局＞

□研究の場所・期間

「片づけ・整理整頓教室」は帝京大学内で開催し、家庭訪問による整理整頓作業は、あなた(調査研究対象の方）のご自宅に専門業者が訪問して行います。

研究の実施期間は帝京大学倫理審査終了後から参加者を募集し、調査開始後 7 か月後までが、あなた(調査研究対象の方）の調査実施期間ですが、最終調査終了時期は、2017 年 3 月までとなります。

□研究試料と情報の取り扱い

調査によって得られた居室の写真を含むデータは ID 番号によって管理され、個人が特定される事はありません。データの取り扱いは、パソコンに保管してパソコンには重層的にセキュリティーをかけ、紙媒体の情報はデータ入力後、居室の写真は、判定した乱雑状態レベルを入力後速やかに裁断します。また、研究代表者は、調査等の実施に係わる文書（申請書類の控え、倫理委員会からの通知文書、各種申請書・報告書の控、対象者識別コードリスト、同意書、報告書等の控、その他データの信頼性を保証するのに必要な書類または記録などは保存し、研究発表後 5 年後に廃棄します。モニタリング・監査を行う場合の閲覧や、研究組織員が行う将来の研究での使用の際も、ID 番号ごとに得点化されたデータのみを取扱い、データから個人が特定されることはありません。

ご自宅に専門業者が訪問する際の個人情報保護に関して、収集する情報と管理、保護に関し別紙誓約書を取り交わし、研究対象者の個人情報が保護されるよう、規則を遵守するよう求めます。

□研究結果の扱い

研究結果および得られた知見は学術学会や帝京大学公衆衛生大学院特殊研究報告書への提出を予定しています。学会発表や論文投稿、報告書記載に当たり、個人情報および個人データが公表されることはありません。

□研究資金源

本研究は平成 26～29 年度「慢性的に片づけられない若年者の実態と効果的介入プログラムの開発」（科学研究費補助金：挑戦的萌芽研究　研究代表者　麻生保子、課題番号 26671045）の研究の一環として行われます。

□利益相反

本研究の実施に際しては特定の企業や団体からの資金援助は受けておりません。また、本臨床研究の利益相反関係は、帝京大学板橋キャンパス利益相反管理委員会の審査を受けております。

□研究参加に伴う負担や支払いの有無

「片づけ・整理整頓教室」参加費用および業者による居宅の整理整頓作業代金、整理整頓作業者の対象者自宅への交通費等への対象者の負担はありません。但し、居宅内整理整頓作業を実施する際に、物品を収納する戸棚類が必要となった場合で、対象者が購入等を決定した場合は、対象者の自己負担とします。また、教室参加の際の交通費および居室の写真撮影、データ送付に要する費用は対象者の自己負

担となります。居宅状況調査や写真データを送付していただいた際に200円から500円程度の文房具またはクオカードの謝礼を予定しております。

□被る可能性のある個人の利益、不利益、人的トラブルを含む有害事象とその対応

研究に参加することで、整理整頓方法について、教室または居宅内整理整頓作業にて専門家より学ぶ機会を得ることができます。また、将来の「溜め込み症」予防の方法論確立の進歩に貢献できる可能性があります。調査中に体調の悪化や著しい情動の変化等が認められた際は、即座に調査を中止します。万が一何らかの医療的措置が必要な事態が大学内で生じた場合は、研究チーム内の医療従事者が適宜対応し、大学内保健室へ同行します。対象者の居宅内整理整頓作業に伴う、家財等の破損や、整理整頓作業による健康被害が認められた場合は、整理整頓業者が加入する損害保険により対応しますが、健康保険の範囲とします。研究期間中の研究者や担当者の言動や態度、配慮に欠けた対応等により対象者が不快と受け取れる事象等があった場合は、研究担当者または下記の研究事務局代表へご連絡ください。

□研究中止の条件

１．あなた(調査研究対象の方）が以下の条件となった際には、調査を中止します。

1) あなた(調査研究対象の方）や、保護者、同居人に方から調査参加の辞退の申し出や同意の撤回があった場合
2) 登録後に調査に適格でないと判明された場合
3) あなた(調査研究対象の方）の健康や生活にとっての不利益が大きく、調査の継続が困難な場合
4) あなた(調査研究対象の方）の体調等により調査継続が好ましくないと判断された場合
5) あなた(調査研究対象の方）の合併症等の増悪により調査実施が困難な場合
6) 調査研究全体が中止された場合
7) その他の理由により研究代表者が調査研究実施を中止することが適当と判断した場合

２．調査研究自体が中止となる条件

1) 研究調査の安全性、有効性に関する重大な情報が得られたとき。
2) 対象者の確保が困難で予定例を達成することが、困難であると判断されたとき。
3) 予定調査数または予定期間に達する前に、調査の目的が達成されたとき。

＊上記の事象が生じた際は、すみやかに大学内の倫理委員会で検討し、検討結果を対象者にご連絡します。

□質問への対応の仕方・連絡先

研究計画書及び研究の方法に関する資料を入手又は閲覧希望の際や質問がある際は研究代表者に連絡し、資料を閲覧・入手、質問できます。連絡先は以下の通りです。

説明日：　　年　　月　　日

説明者：
