# Supplementary material for: Effectiveness of a ‘Workshop on Decluttering and Organising’ programme for teens and middle-aged adults with difficulty decluttering: a study protocol of an open-label, randomised, parallel-group, superiority trial in Japan
Source: BMJ Open. 2017 Jun 10;7(6):e014687. doi: 10.1136/bmjopen-2016-014687 (PMC5541631; doi:10.1136/bmjopen-2016-014687)
Supplement: Supplementary material 4 [file bmjopen-2016-014687supp004.pdf]

Explanation Document for Research Participants
(Individuals 15 years of age or older, Parents or Guardians)

We would like to ask for your participation in, and provide an overview of, the research on the effect of the "Decluttering and Organizing Class" by RCT for young people who have difficulty with organizing (separating participants into groups of those with or without class sessions by drawing in order to fairly monitor the effectiveness). I will explain the research participation fully, based on the following items, so please decide whether you wish to participate (as a participant in the research) or not. We will address your questions or concerns at any time, and you can cancel your participation even after you have signed up for the research. You will not be disadvantaged for any reason due to participation. If you do not meet our research requirements, we may not be eligible for participation.

□Purpose and Meaning of the Research

The purpose of this research is to evaluate the effectiveness of the class, by separating the participants into two groups by drawings: one for those who attend the Decluttering and Organizing Class as well as an organizing work visit at home (the group with class), and the other for those who conduct an organizing work visit at home (the group without class), and targeting those who are determined to have a room condition that is relatively disorganized by answering the questionnaire. Also, the participants of the group cannot select which group they belong to. Both groups will have their room conditions, quality of life, and knowledge and resistance toward organizing, and their self-esteem compared after one month, two months, four months, and seven months, and the effectiveness of the "Decluttering and Organizing Class" will be evaluated.

It is shown that participants whose room conditions are disorderly weaken their ability to organize as their physical energy is weakened. Recently, more and more people are learning how to organize by themselves following so called "tidying up books". Existing research shows that learning about tidying up in class and effective organizing operations are valuable for those who are not good at tidying up. We aim to contribute to the improvement of the quality of life for the entire life of those who are not good at organizing by widely releasing the results of the research, having an effective "Tidying Up and Organizing Class" held by the governments and vendors that support tidying up.

□ Subjects and Method of the Research

We will conduct the pre-research by sending the questionnaire to applicants through open recruitment, and targeting people who can conduct organizing tasks by themselves out of those whose average score was high in the previous evaluation conducted by the research representative (The findings consist of 450 subjects from two universities.) We will separate the

participants into two groups: a group with the class and a group without the class by drawing. For the group with the class, 4 Decluttering & Organizing Class sessions and 1 organizing work session at home by organizing professionals will be offered. For the group with no class, they will have only 1 organizing work session by the professionals so that we can measure whether conducting the class is effective or not. In order to compare the groups, we will observe the room conditions by photos, examination forms, scores for self-esteem, and ask about knowledge and resistance toward organizing, the organizing condition in the past week (frequency of organizing, whether they are embracing the housing environment or not), and the number of visitors to their home in the past one week. (The series of research is called "Questionnaire Evaluation.") Also, we will conduct the evaluation prior to the class, regarding their age, educational background, and work style. It is necessary for you to send the answers to the questionnaire regarding your current house condition periodically, and to also send photos of your home via email or regular mail. (Chart 1)

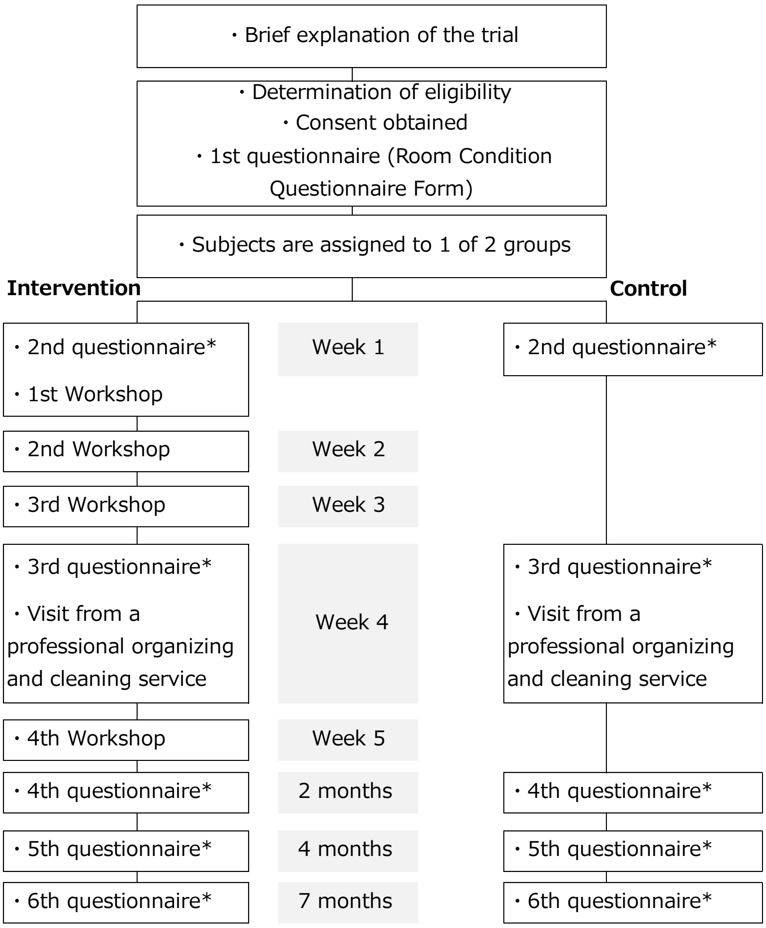

* Room Condition Questionnaire Form. The participants will supply photos of their room/house with their completed questionnaire.

☐ Voluntary Participation and Right to Withdraw Consent

The participants decide whether they will participate in the research or not of their own will, and won't be at any disadvantage even if they refuse participation in the research. Also, even for participants who want to cancel participation during the research, there will be no disadvantage. The participants can even cancel their consent after the research has been conducted. Furthermore, if the participants cancel their consent at the time of the research commencement or after the research, we will delete the data if the data is not yet analyzed. Also, the participants will not be disadvantaged by cancelling their consent. Additionally, in the event that the participants don't participate or cancel their participation, if they wish, we will provide the research findings to them.

□Persons Responsible for and Organization of the Research

This research involves multiple facilities' common research and is mainly done by Teikyo University. The organization structure is as follows.

<Research representative>
< Research members>
< Associate Researchers>

☐ Location and Timeframe of The Research

We will host the "Decluttering & Organizing Class" at Teikyo University. Organizing professionals will visit your home to conduct organizing work sessions with you.

When the research is approved and ready to proceed, we will start recruiting for participants. Seven months after the start will be the end of your (the participants) evaluation period.

☐ Handling of Research Materials and Personal Information

Data collected for the research will be numbered, so no one will know whose data it is. Then the data will be stored in a computer, which is secured with passwords so unauthorized people cannot access the computer. Any information on paper will be shredded and destroyed immediately after they are entered into the computer, and photos of your rooms will be shredded after the review.

Also, the research representative chief securely stores documents used for the research (announcements from the ethics committee, copies of applications and the reports, documents containing the participants numbers, consent, copies of the reports, and other necessary documents), and he or she deletes them after five years. If we check the data in the future to see if the research was done properly or revisit the data for other research projects, we will not be able to identify whose data it is.

We will pursue compliance to protect the personal information of the people targeted for the

research by exchanging Commitment Documents on collected information, management and protection regarding personal information protection when the vendor visits the home.

☐ Handling of Research Results

We plan to submit and publish the research findings to Teikyo University and academia. Also, the class materials for the Decluttering and Organizing Class will be released; however personal information and individual data will not be shared.

☐ Funding for the Research

This research will be done as a part of the study “The reality of young people who are Chronically Disorganized; program development for the effective intervention” (From 2014 to From 2017) (The research subsidy: Challenging *Houga* (Bud flush) Research, Research Representative, Yasuko Aso, Research Number 26671045)

☐ Conflicts of Interest

We don’t receive any funds from specific corporate sponsors or organizations for this research. Also, we will have potential conflicts of interest regarding the research screened by the Committee by Itabashi Campus of Teikyo University.

☐ Cost and Payment for Participation

You are not required to pay for participating in the Decluttering and Organizing Class. You are also not required to pay for the organizing work sessions and any expenses for the vendor’s transportation. However, in the event that the participants (or their parents / guardians) need to purchase closets to store goods when they conduct the organizing work and decide to purchase them, the participants will have to pay for them. It is the participants’ responsibility to pay for any transportation to attend the class, and the cost of mailing their room photos.

☐ Adverse Events

If we see the participants feeling worse or experiencing drastic emotional changes, we will cancel the investigation immediately. If they need to have any necessary medical measures, the medical staff members in the research group will respond to them accordingly, and go to the health room at the University. If the participants see any damage to their furniture or health damage caused by organizing work, the vendor's damage insurance will address the issue, but it is limited to the scope of health insurance. If the participants feel discomforted by the language and attitude and uncompassionate acts by the researchers and other staff members during the research period, please contact the research members or the following research administrative representative.

☐ Possible Advantages and Disadvantages for Individuals

The participants can have the opportunity to learn about organizing by participating in the research, and do not need to pay for participation. Also, they will not be paid for the incentive.

☐ Conditions to Cancel the Research

1. If you (the subject of the research) fall under any of the following conditions, the evaluation will be cancelled.
    1) If you or your parents or those who you live with wish for your withdrawal from the research
    2) If it is determined that you are not eligible for the research
    3) If you suffer a serious setback to your health and lifestyle, and have difficulty continuing the evaluation.
    4) If it is decided that continuing the research is not recommended due to health, etc. ,
    5) If you develop complications from an illness, and have difficulty continuing the evaluation
    6) If the entire research program is cancelled.
    7) If the research representative decides that it is appropriate to stop the examination due to other reasons
2. Conditions under which the entire research program would be cancelled
    1) Significant information about a lack of safety and effectiveness in the research is reported
    2) It is decided that recruiting participants for the study is difficult and it interferes with the schedule.
    3) The research purpose is achieved before the scheduled date or goal of the evaluation is achieved.

* If any of the above matters are observed, the internal screening committee and ethics screening committee at the university will discuss the matter and the decision will be announced.

□ How to Address Questions and Contact Information

If you wish to obtain materials regarding the research plan or the method, to access them, or to ask questions, please contact the research representative. You will be provided with access, and obtain information or an opportunity to ask questions about the materials.

Please contact:

Date of Explanation : Year Month Date

Explained by : ____________________

(English version (actually using Japanese version))
