# Supplementary material for: Effectiveness of a ‘Workshop on Decluttering and Organising’ programme for teens and middle-aged adults with difficulty decluttering: a study protocol of an open-label, randomised, parallel-group, superiority trial in Japan
Source: BMJ Open. 2017 Jun 10;7(6):e014687. doi: 10.1136/bmjopen-2016-014687 (PMC5541631; doi:10.1136/bmjopen-2016-014687)
Supplement: Supplementary material 5 [file bmjopen-2016-014687supp005.pdf]

同意書　（12 歳～14 歳ご本人用）

医学医療技術学部長殿

研究テーマ：「整理整頓が苦手な若年者を対象とした、無作為化比較試験を用いた“片づけ・整理整頓教室”の効果に関する研究（くじ引きで教室をする群としない群を分け、教室の効果を見る研究）」

私は、この研究について書類をもとに、下の内容について説明を受け、十分理解のうえ、自分の意志でこの調査に参加することを決めました。

説明を受けた内容：

□ 調査の目的・役割
□ 誰に調査を行うかとその方法
□ 私が調査への参加や取消しをすることが自由に決められることについて
□ この調査を行う人とその人が働く場所
□ 調査の場所・期間
□ 個人情報の守り方
□ 調査結果の活用方法
□ 調査に必要な費用の出どころ
□ この調査に関わることによる損・得に関わる内容
□ 私や保護者の負担や支払いについて
□ 私や保護者が損をすることや困ることが起きた時の対応について
□ この調査が中止になる時の条件
□ 質問の仕方・連絡先

平成　　年　　月　　日
本人氏名（直筆）

説明者の所属・部署

説明者の職名・氏名（自署）

印
