# Supplementary material for: Effectiveness of a ‘Workshop on Decluttering and Organising’ programme for teens and middle-aged adults with difficulty decluttering: a study protocol of an open-label, randomised, parallel-group, superiority trial in Japan
Source: BMJ Open. 2017 Jun 10;7(6):e014687. doi: 10.1136/bmjopen-2016-014687 (PMC5541631; doi:10.1136/bmjopen-2016-014687)
Supplement: Supplementary material 6 [file bmjopen-2016-014687supp006.pdf]

Consent Form
(For Individuals 12 years old to 14 years old)

Dear Department Head of Medical Technology of Teikyo University

Research Title: Research on the effect of “Decluttering and Organizing Class” using random comparison experiment for the young people who have difficulty in organizing their rooms (separating in groups with or without class sessions by drawing in order to monitor the effects)

I received a document containing the following information from a person who explained the research, and having fully understood it, agree to participate in the research voluntarily.

What was explained to me

- ☐ Purpose and meaning of the research
- ☐ Subjects and method of the research
- ☐ Voluntary participation and right to withdraw consent
- ☐ Personnel responsible for and organization of the research
- ☐ Location and timeframe of the research
- ☐ Handling of research materials and personal information
- ☐ How the research results will be used
- ☐ Funding for the Research
- ☐ Conflicts of interest
- ☐ Any cost to the participants
- ☐ Potential advantages and disadvantages such as adverse events that may cause difficulties and how to handle them
- ☐ Conditions under which the research may be cancelled
- ☐ How to address questions and contact information

Year, Month, Date

Name (Signature)

Seal

The parents or guardians (If the person is a minor, or if there is anyone who lives together with him/her)

Name

Seal

Department of the person who provided the explanation

Name and Title of the person who provided the explanation (Signature) Seal

＊ （English version (actually using Japanese version)
