# Supplementary material for: Effectiveness of a ‘Workshop on Decluttering and Organising’ programme for teens and middle-aged adults with difficulty decluttering: a study protocol of an open-label, randomised, parallel-group, superiority trial in Japan
Source: BMJ Open. 2017 Jun 10;7(6):e014687. doi: 10.1136/bmjopen-2016-014687 (PMC5541631; doi:10.1136/bmjopen-2016-014687)
Supplement: Supplementary material 7 [file bmjopen-2016-014687supp007.pdf]

同意書　（15歳以上本人および保護者・代表同居人用）

帝京大学医療技術学部長殿

研究課題名：「整理整頓が苦手な若年者を対象の無作為化比較試験を用いた“片づけ・整理整頓教室”の効果に関する研究（くじ引きで教室をする群としない群を分け、教室の効果を見る研究）」

私は、この研究について説明者から文書により下記の項目について説明を受け、十分理解のうえ自由意思により本研究に参加することに同意します。

説明を受けた項目：

☐ 研究の目的・意義
☐ 研究の対象と方法
☐ 研究への自由意思参加・同意取消しの自由
☐ 研究の責任者・組織
☐ 研究の場所・期間
☐ 研究試料と情報の取り扱い
☐ 研究結果の扱い
☐ 研究資金源
☐ 利益相反
☐ 研究参加者の負担や支払いの有無
☐ 被る可能性のある個人の利益、不利益、人的トラブルを含む有害事象とその対応
☐ 研究中止の条件
☐ 質問への対応の仕方・連絡先

平成　　年　　月　　日

本人氏名（自署）

印

保護者・同居の代表者（未成年または同居の方がいらっしゃる場合）

氏名

印

説明者の所属・部署

説明者の職名・氏名（自署）

印
