# Supplementary material for: Effectiveness of a ‘Workshop on Decluttering and Organising’ programme for teens and middle-aged adults with difficulty decluttering: a study protocol of an open-label, randomised, parallel-group, superiority trial in Japan
Source: BMJ Open. 2017 Jun 10;7(6):e014687. doi: 10.1136/bmjopen-2016-014687 (PMC5541631; doi:10.1136/bmjopen-2016-014687)
Supplement: Author's manuscript [file bmjopen-2016-014687.draft_revisions.pdf]

BMJ Open

Effectiveness of "Workshop on Decluttering and Organizing" program for teens and middle-aged adults with difficulty decluttering: a study protocol of an open-label, randomized, parallel-group, superiority trial, in Japan.

Journal:	*BMJ Open*
Manuscript ID	bmjopen-2016-014687
Article Type:	Protocol
Date Submitted by the Author:	13-Oct-2016
Complete List of Authors:	Aso, Yasuko; Teikyo University, Department of Nursing, Faculty of Medical Technology<br>Yamaoka, Kazue; Teikyo University, Graduate School of Public Health<br>Nemoto, Asuka; Teikyo University, Graduate School of Public Health<br>Naganuma, Yuki; Teikyo University, Department of Nursing, Faculty of Medical Technology<br>Saito, Masashige; Nihon Fukushi University, Department of Social Welfare
**Primary Subject Heading**:	Public health
Secondary Subject Heading:	Nursing, Mental health, Health services research
Keywords:	Saving Inventory–Revised, Workshop on Decluttering and Organizing, Study protocol, Randomized controlled trial, Teens and midd, Protocols & guidelines < HEALTH SERVICES ADMINISTRATION & MANAGEMENT, PUBLIC HEALTH

SCHOLARONE™
Manuscripts

For peer review only

Effectiveness of "Workshop on Decluttering and Organizing" program for teens and middle-aged adults with difficulty decluttering: a study protocol of an open-label, randomized, parallel-group, superiority trial, in Japan.

Yasuko Aso[1,2], Kazue Yamaoka[2], Asuka Nemoto[2], Yuki Naganuma[1], Masashige Saito[3]

Author Affiliations
[1] Department of Nursing, Faculty of Medical Technology, Teikyo University, Tokyo, Japan
[2] Teikyo University Graduate School of Public Health, Tokyo, Japan
[3] Department of Social Welfare, Faculty of Social Welfare, Nihon Fukushi University, Aichi, Japan

Correspondence to
Yasuko Aso.
Postal address: 2-1-11,Kaga, Itabashi-ku, Tokyo 173-8605
Email: asou@med.teikyo-u.ac.jp
Tel: +81-3-3964-1211 Ext.46115

Key words: Saving Inventory–Revised, Workshop on Decluttering and Organizing, Study protocol, Randomized controlled trial, Teens and middle-aged adults, Effectiveness

Word 2937

For peer review only

Abstract

Introduction: Hoarding disorders can cause problems with work performance, personal hygiene, health, and wellbeing. These disorders are a growing social problem in Japan. Having difficulty discarding rubbish, decluttering and organizing can signal a future hoarding disorder, and early intervention is important. We developed an educational workshop on decluttering and organizing for teens and adults with difficulty organizing. The objective of this study is to evaluate the effectiveness of the workshop for reducing clutter and improving quality of life among younger people with difficulty decluttering and organizing.

Methods and analysis: An open-label, parallel-group randomized controlled trial will be conducted among volunteers aged 12–55 years with mild difficulty decluttering and organizing. The intervention group will attend the workshop and receive a visit from a professional cleaning company to declutter their living space. The control group will have only the latter. The primary outcome will be the score on the Japanese version of the Saving Inventory–Revised (a higher score indicates a greater tendency to hoard). Secondary outcomes will be scores on the Clutter Image Rating Scale, the Japanese version of the Rosenberg Self-Esteem Scale, and the Roles of Private Space Scale. The results will be examined for differences between the two groups in changes from baseline to 7 months. We will examine crude effects and adjust for gender, age and marital status, using a general linear model for continuous variables and a logistic regression model for dichotomous variables. Sample size was calculated assuming a significance level of 5% (two-tailed), a power of 80%, and an effect size of 0.75. In total, 60 subjects (30 in each group) will be required.

Ethics and dissemination: The study protocol has been approved by the Medical Ethical Committee of Teikyo University (No. 15-065). The findings will be disseminated widely through peer-reviewed publication and conference presentations.

Trial registration number: UMIN000020568. Issue Date: 16 January 2016
Protocol Amendment Number: 07. Author: Y.A.

Strengths and limitations of this study

- The study will be an open-label, parallel-group randomized controlled trial and, to our knowledge, is the first such study on this subject in Japan.
- It will test a simple but feasible intervention for those having difficulty decluttering and organizing.
- The outcomes measures are clearly set using reliable scales.
- The test is adequately powered to detect effects on the outcomes.
- Blinding is not possible because of the type of intervention, which may affect the results.

For peer review only

INTRODUCTION

A hoarding disorder can cause problems with work performance, personal hygiene, health, and wellbeing. Hoarding disorders occasionally take the form of a house full of rubbish, where individuals accumulate trash or clutter over a prolonged period.[1] This poses a number of dangers, such as the potential for fire and falls. The trash or clutter also affects an individual's overall quality of life.[2] This issue is a growing social problem in Japan.

An individual living in such conditions can become isolated from the rest of society. This can be particularly the case for older people.[3] The unsanitary living conditions will eventually become a public health problem affecting neighbors. Hoarding disorders are therefore not only an individual problem but also a problem for society, and a practical solution is warranted from a public health point of view.

Hoarding disorders are often accompanied by a psychiatric disorder such as attention deficit hyperactivity disorder (ADHD) or depression.[4] Signs of hoarding appear in early adolescence[5] and the severity of hoarding increases with age.[6] A hoarding disorder may begin to develop from the age of 13 and tends to worsen over the age of 55.[5,6]

In the United States and Europe, a previous study has estimated that the incidence of hoarding disorders is 1.5%.[7] Another study has reported that cognitive behavioral therapy, either individually or in groups, and home visits are effective at assisting patients with a hoarding disorder.[8] However, this type of treatment is not widely available in Japan despite the aging population. Having difficulty decluttering and organizing may be related to a hoarding disorder, and around 60% of Japanese adults report that they do not like decluttering and organizing cleaning.[9] Japan therefore urgently needs to establish a system of early intervention and support to prevent younger people who have difficulty decluttering and organizing from developing a hoarding disorder.

A previous study examined the state of living space of 450 undergraduates from two universities.[10] The results showed that younger people who had difficulty decluttering and organizing tended to live in cluttered or disorganized living spaces and to have low self-esteem. The study used the Japanese version of the Saving Inventory–Revised (SI-R).[11] This was developed in the United States[12] and translated into Japanese, and its reliability and validity have been verified.[11] The previous study's SI-R scores had a mean of 32.1 points (±13.0) and a normal distribution. SI-R score has been reported as high among people who said that they did not like to clean,[10] but many such people also want to improve the state of their living space.[9,10] There are various ways to learn how to clean effectively in Japan, including workshops, books and professional support, but it is not clear which method is most effective.

We have therefore developed an education program, of a series of workshops on decluttering and organizing, for teens and adults who find it difficult to organize themselves. The workshop emphasizes the importance of having a sense of values in life. It also covers the state of the individual's living space and its relationship with physical and mental health. The purpose of the workshop is to improve recognition of the importance of cleaning and to change behavior.

This study examines behavior 7 months after the intervention, using a control group, and is designed to assess both changes in habits, and also levels of stress. It is impossible to blind this type of intervention, so the study was made open label to minimize bias.

The aim of the study is to evaluate the effectiveness of the program in reducing clutter and improving the quality of life in teens and middle-aged adults who have difficulty decluttering and organizing, and who have a cluttered living space.

METHODS AND ANALYSIS

Study design

The design of this study is an open-label, parallel-group, stratified, randomized controlled trial (RCT). The aim of this trial is to investigate the effect of the workshop plus a professional organizing and cleaning service. We hope that the program will

For peer review only

improve the quality of life and help to prevent a hoarding disorder in younger people who currently have difficulty decluttering and organizing. Participants will be involved for a period of 7 months. The design of the trial is shown in Figure 1. Based on previous experience, it is expected that the study will include around 90% of eligible participants. Perioperative protocols are standardized.

[Insert Figure 1 (Trial design) about here]

Participants

The study will look for volunteers aged between 12 and 55 with mild organization difficulties. They will need to meet all of the inclusion criteria below and not meet any of the exclusion criteria (see Table 1).

Table 1 Inclusion criteria and exclusion criteria

Inclusion criteria

- Individuals who have difficulty decluttering and organizing. （SI-R score≧30）
- Individuals with a total score of four points or more on the Japanese version of the Clutter Image Rating (CIR) scale [Note 1].
- Individuals who are responsible for decluttering and organizing their own rooms.
- Individuals with no planned relocations, extensive renovations of living space, or room changes (to allow comparison of the state of living spaces before and after the intervention).
- Individuals who are able to attend at least three workshop sessions and receive visits from an organizing and cleaning service.
- Individuals who want to have a more organized and tidy living space.
- Individuals who can send photographs of their living spaces at the specified time, i.e. without delay.
- Individuals or a roommate/family member (the informed consent form for minors if the individual is under the age of 15) who consent in writing to their participation in the study, and to having a better organized and tidied living space. If the individual is a minor, consent must be provided by a guardian.
- Individuals between the ages of 12 and 55 at the start of the study.

Exclusion criteria

- Individuals who are unable to organize and tidy up their living spaces during the study period, for example:
  - ✓ Individuals with an illness or disability
  - ✓ Individuals who are not living at the registered residence or are physically unable to regularly organize and tidy up
  - ✓ Individuals in a living space that is so highly cluttered that they are unable to organize and tidy up that space themselves.
- Individuals who harass other attendees while attending the workshop or who are deemed incapable of obeying the specified rules of attendance.
- Individuals who are rendered incapable of participation during this study.
- Individuals suffering from a condition or disability that is deemed to preclude study participation. If there are difficulties with that determination, an individual may be barred from participation at the discretion of a physician.
- Individuals who are otherwise deemed ineligible by an investigator (or sub-investigator).
-

[Note 1] The CIR scale score for the general public is a mean of 3.8 points (±1),[10] so the subjects in this study must have at least that score.

For peer review only

Recruitment and allocation

Subject recruitment

In a previous study, hoarding participants were grouped into three classes: "non-comorbid" hoarding (42%), hoarding with depression (42%), and hoarding with depression and inattention (16%). [16] Participants will be recruited in several ways so that the study population is reasonably representative.

We will recruit participants in several ways, including notices posted at Teikyo University; and open recruitment from notices at an ADHD mutual self-help group, society, helper station and visiting care station. We will also put up posters at helper stations, visiting care stations, community pharmacies, and a university, high school and junior high school in northwest Tokyo. Participants will also be recruited from among the general public via a research recruitment website, an events firm, publicity information sent to cleaning companies, a non-profit organization providing support for people with ADHD and a notice at the university.

Applicants will visit Teikyo University to receive an oral and written explanation of the purpose of the study. After initial agreement, and assessment using the questionnaire, we will determine eligibility.

Subject assignment

We will provide potential participants with an overview of the study. They will be asked to complete a preliminary study to assess their eligibility. Once a potential subject is verified as eligible, their full consent will be obtained and they will be enrolled. Subjects will be enrolled until the required sample size (30 subjects per group) is obtained. Data collection will take about 7 months.

Randomization and blinding

Based on a permuted block method with block size 4 and a random number table created before the start of the study, each subject will be assigned to either intervention or control group. Visits from a professional organizing and cleaning service will be determined based on a random number table that differentiates the assigned groups, but assigns the home organizing and cleaning services at random, to maintain the fairness of this study. Effectiveness of the intervention will be determined using surveys completed by the participants, and sent directly to the Study Office, and a photograph of the room to assess room cleanliness or clutter. The home organizing and cleaning services will not be able to see the results for individual subjects.

Interventions

Intervention group

This group will attend a workshop program, undergo inspections of the cleanliness or clutter of their rooms, and receive visits from an organizing and cleaning service. The workshop will consist of four sessions. Room cleanliness or clutter will be inspected six times. The first inspection will take place once eligibility is verified and consent is obtained. The second will take place before the start of the study. Others will take place 4 weeks, 2 months, 4 months and 7 months after the start of the study (see Figure 1).

Control group

The control group will receive an initial inspection of room cleanliness or clutter once eligibility is verified and consent obtained. The second inspection will take place before the intervention group starts the workshop. Further inspections will take place 4 weeks, 2 months, 4 months, and 7 months after the start of the study. Members of the control group who wish to attend the workshop may do so after the study period ends.

The content of the workshop

The workshop's purpose is to improve participant behavior and develop habits of

For peer review only

decluttering and organizing. It starts by covering the relationship between physical and mental health and the state of the living space. It then discusses participants' ideal life.

Table 2 Syllabus for "Workshop on Decluttering and Organizing"

1st	<Reduce><br>・Orientation and goal setting.<br>・Understanding the importance of working as a group.<br>・Three fundamentals for healthy and comfortable living (reduce, organize, maintain).<br>・Organizing in small steps: around your desk and your bed<br>・Explanation of homework.
2nd	<Organize><br>・Review of previous session<br>・The relation between decluttering and organizing and health (fall prevention, allergic disease)<br>・Organizing in small steps: the kitchen<br>・Explanation of homework.
3rd	・Review of previous session<br>・Solutions for people who have difficulty discarding things and buy (or receive) unnecessary things<br>・Organizing in small steps: a place to relax<br>・Explanation of homework.
4th	< Maintain><br>・Review of previous session<br>・Organizing in small steps: closets and wardrobes<br>・Schedule of questionnaire submission

Outcome measures and subject's characteristics

Primary outcome

The primary outcome is the score on the Japanese version of the Saving Inventory-Revised (SI-R). The SI-R score is an index used to gauge the difficulties posed by a hoarding disorder. It measures mental anguish caused by hoarding, and the impacts of hoarding on life. A higher score indicates a greater tendency to hoard.[11,12]

The primary effect size is the difference changes in SI-R score from baseline to 7 months between the two groups. The hypothesis of this study is that the mean SI-R score of the intervention group will decrease more than that of the control group.

Secondary outcomes

Secondary outcomes include:

- Score on the Clutter Image Rating (CIR) scale, which measures clutter using photographs of rooms (a higher score indicates more clutter).[13,14]
- Score on the Japanese version of the Rosenberg Self-Esteem Scale (RSES).[17] This measures the degree of self-respect and the value placed on the self. A high score shows stronger feelings of self-esteem.
- Score on the Roles of Private Space Scale (RPSS). This linear measure observes the function , the necessity and securement level of private space. [18]

These outcomes will be used to examine the amount of decluttering and organizing, including frequency of cleaning and number of visitors in a week. They will allow the researchers to assess the state of subjects' living spaces and their ability to declutter and organize as a result of the workshop.

Subject characteristics

During the first meeting, participants will provide information about their gender, age, co-morbidities, previous history, medication, number of roommates/family members in their household, number and size of living spaces, how often their living space is

For peer review only

organized and tidied up, number of visitors to their living space in the previous week, whether they have difficulty decluttering and organizing, and are living with a roommate or family members, and aspects of social status such as occupation, marital status, educational background, and work pattern (whether they are working on a shift basis).

Statistical Analysis

Sample size

A previous study divided 46 patients with a hoarding disorder (mean age: 53.9 years) into two groups. One group of 23 subjects were scheduled for 26 sessions of cognitive behavioral therapy approximately weekly. The second group of 23 subjects had yet to undergo cognitive behavioral therapy.[8] A comparison of the two groups indicated that subjects who had completed the 12th session of cognitive behavioral therapy had a 10-point drop in their SI-R score. A meta-analysis in another study indicated that intervention was more effective in younger individuals than older ones. This study therefore assumed a potential 10-point improvement in SI-R score.[19] Previous results indicated that the SI-R score had a normal distribution and SD was 13.0.[10] The sample size was therefore calculated as 28 per group under the assumptions of a significance level of 5% (two-tailed), a power of 80%, and an effect size of 0.75. Considering the likely drop-out rate, we set the sample size needed for this trial as 60 subjects in total, 30 in each group.

Statistical Analysis

Analyses will be conducted under the intention-to-treat (ITT) principle. The main target set for analysis is the full analysis set (FAS). Missing data will be permutated under the assumption of missing at random (MAR) following the last observation carried forward (LOCF) principle. A sensitivity analysis will be performed using the multiple imputation (MI) method. The secondary target set for the analyses will be the per protocol set (PPS).

Summary statistics (maximum, median, minimum, mean, and standard deviation) will be calculated for all continuous data, and frequency and proportion for categorical data.

The differences in effect sizes between the two groups will be examined using a t-test. Differences will be assessed in a general linear model by adjusting for gender, age and marital status at the start of the study, and outcome measure at the baseline. Crude and adjusted odds ratios will be calculated for binary variables, and the two groups will be compared using logistic regression analysis. The significance level for testing will be set at $p < 0.05$ (two-tailed).

Timeframe of the study

Participants were registered between January and May 2016. Eight workshops have been run between 3 February and 11 June 2016. Data is currently being collected.

Data management

Personal information obtained in this study will be coded. Participant files will be stored in numerical order in a secure but accessible place and manner. Data will be anonymized before analysis. Data will be kept on a password-protected computer with limited access. The study organizer will keep all documents related to this trial in a locked cabinet. Five years after the study is presented, or three years after the final publication of the results (whichever is later), all documents will be disposed of using a document destruction service contracted by the University.

Conditions for discontinuation of this study and actions in that event

When significant information about the safety or effectiveness of the intervention is obtained, the study organizer will determine whether to continue the study.

Treatment of subjects after this study is conducted

If the intervention group is performing significantly better than the control group at the

For peer review only

end of the study, the control group will be informed. Members of the control group who wish to do so may then attend the workshop after the conclusion of this study.

Monitoring

Information on when the study begins, the conduct of the study (sample size), ethical considerations that have been made, the occurrence of any detrimental or adverse events, the results of the study, and registration of the study with a public database will be submitted to the ethics committee in an annual interim report. A report will also be submitted to the ethics committee upon the conclusion of the study, and when the final results are presented.

Follow-up of adverse events

This study involves attending a workshop program to help people declutter and organize, and receiving visits from a professional organizing and cleaning service. There is little likelihood of any health hazards to the subjects. If any serious adverse events occur, they will be reported in line with the standard operating procedure on Reporting Serious Adverse Events in Clinical Research.

Ethics and dissemination

The study protocol has been approved by the Medical Ethical Committee of Teikyo University (No. 15-065). The findings will be disseminated widely through peer-reviewed publication and conference presentations.

Conflicts of interest

Based on a random number table, home organizing and cleaning services will be randomly assigned to clean the subjects' living space (the organizers will be blind to whether they are in the control or intervention group), to maintain the fairness of this study. Effectiveness of the intervention will be determined using a survey completed by the subject and sent directly to the Study Office, together with a photograph to assist determination of room cleanliness or clutter. Home organizing and cleaning services will not be able to see any results for individual subjects.

DISCUSSION

One aim of this study is to assess the effect of an intervention (a workshop) on the state of living spaces and the quality of life of younger people who have difficulty decluttering and organizing and who therefore have a cluttered living space. If the approach is found to be effective, then the findings of this study will be widely publicized and the workshop materials will be made widely available, so that it can be conducted by others, including administrative bodies and professional organizers and cleaners. This should improve the quality of life and help to prevent a hoarding disorder in younger people who have difficulty decluttering and organizing.

This study has strengths and weaknesses. Blinding is not possible, because of the type of intervention. It is impossible to create a convincing placebo for the workshop on decluttering and organizing, but this lack of blinding may affect the results. As far as possible, subjects need to have an experience equivalent to undergoing training from a professional organizing and cleaning service.

Acknowledgments

We are grateful for advice from Yukiko Mochizuki, Yuka Nojiri, and Mihoko Shimozono about preparing a study protocol.

The text for the workshop was prepared with help from Keiko Takayama and Mayumi Takahara.

Contributors:

For peer review only

YA conceived the study. KY, NA were involved in the development of the intervention and design of the trial. NY has contributed to the acquisition and interpretation of the data.SM was been involved in drafting the work or revising it critically for important intellectual content. All authors have read and approved the final manuscript for publication.

Funding
This work was supported by JSPS KAKENHI Grant Number JP26671045(2014–2017) Grant-in-Aid for Scientific Research on "The state of young people who chronically fail to organize or tidy up and development of a program for effective intervention" (Challenging Exploratory Research, Study Organizer: Yasuko Aso, grant no. 26671045).

Competing interests statement
No financial assistance or free services will be received from any company or organization during this study. The researchers have no equity, such as stock, in any of the companies involved, and are not involved in their management, nor have the researchers received any services, free goods or pharmaceuticals, or discounts from those companies.

Home organizing and cleaning services will, based on a random number table, be randomly assigned to clean a subject's living space to maintain the fairness of this study. Effectiveness of the intervention will be determined based on a survey sent by the subject directly to the Study Office and a determination of room cleanliness or clutter based on photographs. Home organizing and cleaning services will not be able to view results for individual subjects.

Subjects' consent
Obtained.

Ethics approval
This trial has been approved by Teikyo University Review Board15-065

Data sharing statement
The informed consent documents are available on request.

REFERENCES

1. American Psychiatric Association. Diagnostic and statistical manual of mental disorders (DSM). Washington, DC: American Psychiatric Association .1994:143–7.
2. Tolin DF, Frost RO, Steketee G, et al. The economic and social burden of compulsive hoarding. *Psychiatry Res* 2008;160(2):200–11.
3. Kishi E, Nojiri Y, Yonezawa J, et al. An analysis of forms of intervention by nurses at comprehensive community support centers in cases of self-neglect. *Journal of the Japan Academy for the Prevention of Elder Abuse* 2014;10(1):106–20.
4. Hall BJ, Tolin DF, Frost RO, et al. An exploration of comorbid symptoms and clinical correlates of clinically significant hoarding symptoms. *Depress Anxiety* 2013;30(1):67–76.
5. Grisham JR, Frost RO, Steketee G, et al. Age of onset of compulsive hoarding. *J Anxiety Disord* 2006;20(5):675–86.
6. Eckfield MB, Wallhagen MI. The synergistic effect of growing older with hoarding behaviors. *Clin Nurs Res* 2013;22(4):475–91.
7. Nordsletten AE. Epidemiology of hoarding disorder. *Br J Psychiatry* 2013;203:445–52.
8. Tolin DF, Frost RO, Steketee G. Cognitive behavioral therapy for hoarding disorder: A meta-analysis. *Depress Anxiety* 2015;32(3):158–66.
9. Katadukeha nigatedesuka? [Do you have difficulty organizing your home?] Asahi

For peer review only

news peper. 2013 Mar 30,;sect.b 10(col.1).
10. Aso Y, Masashige S, Yuka N. Attitudes of undergraduates studying nursing or welfare work towards organizing and tidying up and the state of their living spaces. [abstract]. Nagasaki; Japan, The 74th Annual Meeting of the Japanese Society of Public Health;2015.pp294.
11. Aki T, Ken-ichi K, Toko I, et al. A consideration of clinical characteristics of non-clinical hoarding among Japanese adolescents. *Japanese Society of Anxiety and Related Disorders* (2015;6(2):72–85.
12. Frost RO, Steketee G, Grisham JR. Measurement of compulsive hoarding: saving inventory-revised. *Behav Res Ther* (2004;42(10):1163–82.
13. Frost, RO, Steketee G. Compulsive Hoarding and Acquiring: Therapist Guide. Japanese edition. Toko I, translator. Tokyo: Kanekoshobou 2013:170–3.
14. Frost RO, Steketee G, Tolin DF et al. Development and validation of the Clutter Image Rating. *J Psychopathol Behav Assess* 2008;30(3):193–203.
15. Hall BJ, Tolin DF, Frost RO, et al. An exploration of comorbid symptoms and clinical correlates of clinically significant hoarding symptoms. *Depress Anxiety* 2013;30(1):67–76.
16. Varness K. The ICD Guide to Challenging Disorganization: For Professional Organizers. Japanese edition. Junko B, translator. Tokyo: Hazuki Press 2013:16–21.
17. MarikoY, Yutaka M, Yukiko Y. The Structure of Perceived Aspects of Self. *Japanese Association of Educational Psychology*. 1982;30(1):64–8.
18. Tomari S, Yoshida F. Psychological review of meanings of private space and its privacy research and proposal for functions: new models. *Tsukuba Psychological Research* 1998;20:173–90.
19. Steketee G, Frost RO, Tolin D, et al. Waitlist-controlled trial of cognitive behavior therapy for hoarding disorder. *Depress Anxiety* (2010;27(5):476–84.

FIGURE TITLES

Figure 1. Trial design

For peer review only

Figure 1 Trial design

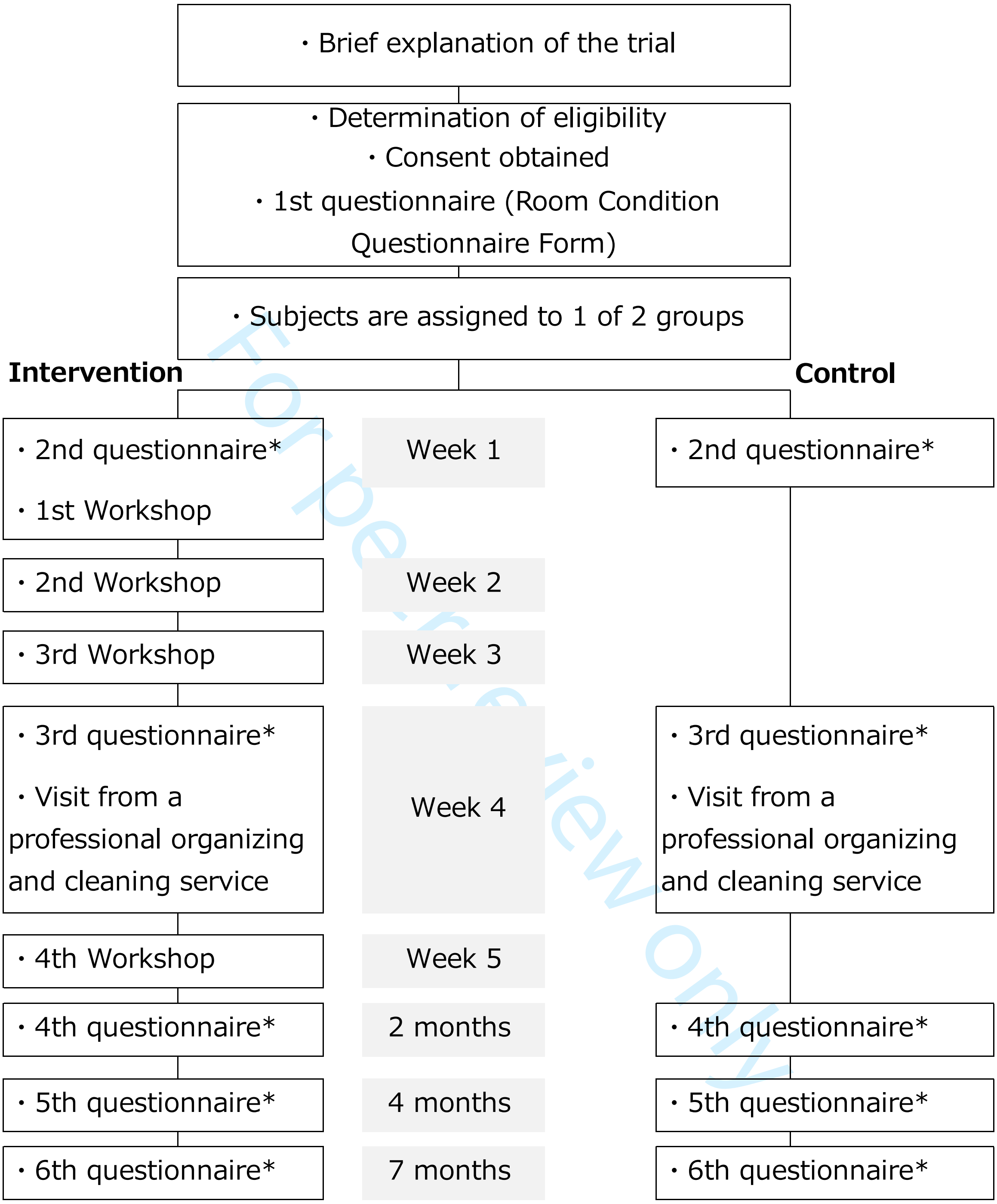

* Room Condition Questionnaire Form. The participants will supply photos of their room/house with their completed questionnaire.

For peer review only

CONSORT 2010 checklist of information to include when reporting a randomised trial*

Section/Topic	Item No	Checklist item	Reported on page No
**Title and abstract**			
	1a	Identification as a randomised trial in the title	1
	1b	Structured summary of trial design, methods, results, and conclusions (for specific guidance see CONSORT for abstracts)	2
**Introduction**			
Background and objectives	2a	Scientific background and explanation of rationale	3
	2b	Specific objectives or hypotheses	3
**Methods**			
Trial design	3a	Description of trial design (such as parallel, factorial) including allocation ratio	3
	3b	Important changes to methods after trial commencement (such as eligibility criteria), with reasons	4
Participants	4a	Eligibility criteria for participants	4
	4b	Settings and locations where the data were collected	1
Interventions	5	The interventions for each group with sufficient details to allow replication, including how and when they were actually administered	5-7
Outcomes	6a	Completely defined pre-specified primary and secondary outcome measures, including how and when they were assessed	6
	6b	Any changes to trial outcomes after the trial commenced, with reasons	-
Sample size	7a	How sample size was determined	7
	7b	When applicable, explanation of any interim analyses and stopping guidelines	-
Randomisation:			
Sequence generation	8a	Method used to generate the random allocation sequence	5
	8b	Type of randomisation; details of any restriction (such as blocking and block size)	5
Allocation concealment mechanism	9	Mechanism used to implement the random allocation sequence (such as sequentially numbered containers), describing any steps taken to conceal the sequence until interventions were assigned	5
Implementation	10	Who generated the random allocation sequence, who enrolled participants, and who assigned participants to interventions	5
Blinding	11a	If done, who was blinded after assignment to interventions (for example, participants, care providers, those	5,9

For peer review only

For peer review only - http://bmjopen.bmj.com/site/about/guidelines.xhtml

		assessing outcomes) and how	
	11b	If relevant, description of the similarity of interventions	-
Statistical methods	12a	Statistical methods used to compare groups for primary and secondary outcomes	7
	12b	Methods for additional analyses, such as subgroup analyses and adjusted analyses	7
**Results**			
Participant flow (a diagram is strongly recommended)	13a	For each group, the numbers of participants who were randomly assigned, received intended treatment, and were analysed for the primary outcome	Figure1
	13b	For each group, losses and exclusions after randomisation, together with reasons	-
Recruitment	14a	Dates defining the periods of recruitment and follow-up	7
	14b	Why the trial ended or was stopped	-
Baseline data	15	A table showing baseline demographic and clinical characteristics for each group	-
Numbers analysed	16	For each group, number of participants (denominator) included in each analysis and whether the analysis was by original assigned groups	-
Outcomes and estimation	17a	For each primary and secondary outcome, results for each group, and the estimated effect size and its precision (such as 95% confidence interval)	-
	17b	For binary outcomes, presentation of both absolute and relative effect sizes is recommended	-
Ancillary analyses	18	Results of any other analyses performed, including subgroup analyses and adjusted analyses, distinguishing pre-specified from exploratory	-
Harms	19	All important harms or unintended effects in each group (for specific guidance see CONSORT for harms)	-
**Discussion**			
Limitations	20	Trial limitations, addressing sources of potential bias, imprecision, and, if relevant, multiplicity of analyses	2,8
Generalisability	21	Generalisability (external validity, applicability) of the trial findings	8
Interpretation	22	Interpretation consistent with results, balancing benefits and harms, and considering other relevant evidence	8
**Other information**			
Registration	23	Registration number and name of trial registry	2
Protocol	24	Where the full trial protocol can be accessed, if available	2,9
Funding	25	Sources of funding and other support (such as supply of drugs), role of funders	9

*We strongly recommend reading this statement in conjunction with the CONSORT 2010 Explanation and Elaboration for important clarifications on all the items. If relevant, we also recommend reading CONSORT extensions for cluster randomised trials, non-inferiority and equivalence trials, non-pharmacological treatments, herbal interventions, and pragmatic trials. Additional extensions are forthcoming: for those and for up to date references relevant to this checklist, see www.consort-statement.org.

For peer review only - http://bmjopen.bmj.com/site/about/guidelines.xhtml

BMJ Open

Effectiveness of “Workshop on Decluttering and Organizing” program for teens and middle-aged adults with difficulty decluttering: a study protocol of an open-label, randomized, parallel-group, superiority trial in Japan.

Journal:	*BMJ Open*
Manuscript ID	bmjopen-2016-014687.R1
Article Type:	Protocol
Date Submitted by the Author:	31-Dec-2016
Complete List of Authors:	Aso, Yasuko; Teikyo University, Department of Nursing, Faculty of Medical Technology<br>Yamaoka, Kazue; Teikyo University, Graduate School of Public Health<br>Nemoto, Asuka; Teikyo University, Graduate School of Public Health<br>Naganuma, Yuki; Teikyo University, Department of Nursing, Faculty of Medical Technology<br>Saito, Masashige; Nihon Fukushi University, Department of Social Welfare
**Primary Subject Heading**:	Public health
Secondary Subject Heading:	Nursing, Mental health, Health services research
Keywords:	Saving Inventory–Revised, Workshop on Decluttering and Organizing, Study protocol, Randomized controlled trial, Teens and midd, Protocols & guidelines < HEALTH SERVICES ADMINISTRATION & MANAGEMENT, PUBLIC HEALTH

SCHOLARONE™
Manuscripts

Effectiveness of "Workshop on Decluttering and Organizing" program for teens and middle-aged adults with difficulty decluttering: a study protocol of an open-label, randomized, parallel-group, superiority trial in Japan.

Yasuko Aso[1,2], Kazue Yamaoka[2], Asuka Nemoto[2], Yuki Naganuma[1], Masashige Saito[3]

Author Affiliations
[1] Department of Nursing, Faculty of Medical Technology, Teikyo University, Tokyo, Japan
[2] Teikyo University Graduate School of Public Health, Tokyo, Japan
[3] Department of Social Welfare, Faculty of Social Welfare, Nihon Fukushi University, Aichi, Japan

Correspondence to
Yasuko Aso.
Postal address: 2-1-11 Kaga, Itabashi-ku, Tokyo 173-8605
Email: asou@med.teikyo-u.ac.jp
Tel: +81-3-3964-1211 Ext.46115

Key words: Saving Inventory–Revised, Workshop on Decluttering and Organizing, Study protocol, Randomized controlled trial, Teens and middle-aged adults, Effectiveness

Words 3359

For peer review only

For peer review only - http://bmjopen.bmj.com/site/about/guidelines.xhtml

Abstract

Introduction: Hoarding disorder can cause problems with work performance, personal hygiene, health, and well-being. The disorder is a growing social problem in Japan. Having difficulty discarding rubbish, decluttering and organizing can signal a future hoarding disorder, and early intervention is important. We developed an educational workshop on decluttering and organizing for teens and adults with difficulty organizing. The objective of this study is to evaluate the effectiveness of the workshop for reducing clutter and improving quality of life among younger people with difficulty decluttering and organizing.

Methods and analysis: An open-label, parallel-group randomized controlled trial will be conducted among volunteers aged 12–55 years with mild difficulty decluttering and organizing. The intervention group will attend the workshop and receive a visit from a professional cleaning company to declutter their living space. The control group will have only the latter. The primary outcome will be the score on the Japanese version of the Saving Inventory–Revised.Secondary outcomes will be scores on the Clutter Image Rating Scale, the Japanese version of the Rosenberg Self-Esteem Scale, and the Roles of Private Space Scale. The results will be examined for differences between the two groups in changes from baseline to 7 months. We will examine crude effects and adjust for gender, age and marital status, using a general linear model for continuous variables and a logistic regression model for dichotomous variables. Sample size was calculated assuming a significance level of 5% (two-tailed), a power of 80%, and an effect size of 0.75. In total, 60 subjects (30 in each group) will be required.

Ethics and dissemination: The study protocol has been approved by the Medical Ethical Committee of Teikyo University (No. 15-065) The findings will be disseminated widely through peer-reviewed publication and conference presentations.

Trial registration number: UMIN000020568. Issue Date: 16 January 2016

Strengths and limitations of this study

- The study will be an open-label, parallel-group randomized controlled trial and, to our knowledge, is the first such study on this subject in Japan.
- It will test a simple but feasible intervention for those having difficulty decluttering and organizing.
- The outcomes measures are clearly set using reliable scales.
- The test is adequately powered to detect effects on the outcomes.
- Blinding is not possible because of the type of intervention, which may affect the results.

For peer review only - http://bmjopen.bmj.com/site/about/guidelines.xhtml

INTRODUCTION

A hoarding disorder can cause problems with work performance, personal hygiene, health, and wellbeing. Hoarding disorder occasionally takes the form of a house full of rubbish, where individuals accumulate trash or clutter over a prolonged period.[1] This poses a number of dangers, such as the potential for fire and falls. The trash or clutter also affects an individual's overall quality of life.[2] This issue is a growing social problem in Japan.

An individual living in such conditions can become isolated from the rest of society. This can be particularly the case for older people.[3] The unsanitary living conditions will eventually become a public health problem affecting neighbors. Hoarding disorder is therefore not only an individual problem but also a problem for society, and a practical solution is warranted from a public health point of view.

Hoarding disorder is often accompanied by psychiatric disorders such as attention deficit hyperactivity disorder (ADHD), depression, or obsessive-compulsive disorder (OCD) [4-10]. Its relationship with distress tolerance and anxiety sensitivity has also been pointed out in a sample of young adults. [11] Signs of hoarding appear in early adolescence and the severity of hoarding increases with age. A hoarding disorder may begin to develop from the age of 13 and tends to worsen over the age of 55.[12,13] These points suggest prevention in the young generation is important and we therefore targeted those aged 12–55 years old. As age 12 is the middle junior high school entrance age in the Japanese education system, we used this instead of 13.

Concerning the United States and Europe, a previous study estimated that the incidence of hoarding disorder is 1.5%.[14] Another study reported that cognitive behavioral therapy, either individually or in groups, and home visits are effective at assisting patients with hoarding disorder.However, these cognitive behavioral therapies needed relatively long intervention periods and the subjects were older people [15-17].

Additionally, this type of treatment is not widely available in Japan, despite the aging population. Having difficulty decluttering and organizing may be related to a hoarding disorder, and around 60% of Japanese adults report they do not enjoy performing these activities.[18] In an intervention study that examined education to alleviate hoarding behavior through improving anxiety sensitivity in young subjects, the beneficial effects were not clearly differentiated from those of general health education.[11] Effective education is needed to prevent hording behavior in younger people.

Japan, therefore, has urgent need to establish a system of early intervention and support to prevent younger people who have difficulty decluttering and organizing from developing a hoarding disorder.

A previous study examined the state of living space of 450 undergraduates from two universities.[19] The results showed that younger people who had difficulty decluttering and organizing tended to live in cluttered or disorganized living spaces and to have low self-esteem. The study used the Japanese version of the Saving Inventory–Revised (SI-R).[20] This was developed in the United States[21] and translated into Japanese, and its reliability and validity have been verified.[20] The previous study's SI-R scores had a mean of 32.1 points (±13.0) and a normal distribution. SI-R score has been reported as high among people who said that they did not like to clean,[19] but many such people also want to improve the state of their living space.[18,19] There are various ways to learn how to clean effectively in Japan, including workshops, books and professional support, but it is not clear which method is most effective.

We have therefore developed an education program, of a series of workshops on decluttering and organizing, for teens and adults who find it difficult to organize. In the workshops, we emphasize the importance of a sense of values (such as necessity) that each person has for things. It also covers the state of the individual's living space and its relationship with physical and mental health. The purpose of the workshop is to improve recognition of the importance of cleaning and to change behavior.

For peer review only - http://bmjopen.bmj.com/site/about/guidelines.xhtml

This study examines behavior 7 months after the intervention, using a control group, and is designed to assess both changes in habits, and also levels of stress. It is impossible to blind this type of intervention, so the study was made open label to minimize bias.

The aim of the study is to evaluate the effectiveness of the program in reducing clutter and improving the quality of life in teens and middle-aged adults who have difficulty decluttering and organizing, and who have a cluttered living space.

METHODS AND ANALYSIS

Study design

The design of this study is an open-label, parallel-group, stratified, randomized controlled trial (RCT). The aim of this trial is to investigate the effect of the workshop plus a professional organizing and cleaning service. We hope that the program will improve the quality of life and help to prevent a hoarding disorder in younger people who currently have difficulty decluttering and organizing. Participants will be involved for a period of 7 months. The design of the trial is shown in Figure 1. Based on previous experience, it is expected that the study will include around 90% of eligible participants. Perioperative protocols are standardized.

[Insert Figure 1 (Trial design) about here]

Participants

The study will look for volunteers aged between 12 and 55 with mild organization difficulties. They will need to meet all of the inclusion criteria below and not meet any of the exclusion criteria (see Table 1).

Table 1 Inclusion criteria and exclusion criteria

Inclusion criteria

- Individuals who have difficulty decluttering and organizing. (SI-R score≥30)
- Individuals with a total score of four points or more on the Japanese version of the Clutter Image Rating (CIR) scale [Note 1].
- Individuals who are responsible for decluttering and organizing their own rooms.
- Individuals with no planned relocations, extensive renovations of living space, or room changes (to allow comparison of the state of living spaces before and after the intervention).
- Individuals who are able to attend at least three workshop sessions and receive visits from an organizing and cleaning service.
- Individuals who responded in the questionnaire, “I want to organize my room according to my will”.
- Individuals who can send photographs of their living spaces at the specified time, i.e. without delay.
- Individuals or a roommate/family member (the informed consent form for minors if the individual is under the age of 15) who consent in writing to their participation in the study, and to having a better organized and tidied living space. If the individual is a minor, consent must be provided by a guardian.
- Individuals between the ages of 12 and 55 at the start of the study.

Exclusion criteria

- Individuals who are unable to organize and tidy up their living spaces during the study period, for example:
  - ✓ Individuals with an illness or disability
  - ✓ Individuals who are not living at the registered residence or are physically unable to regularly organize and tidy up
- Individuals living in a space that is too messy (state of clutter judged as too great to organize even in a 2.5-hour visit by two experts).

For peer review only - http://bmjopen.bmj.com/site/about/guidelines.xhtml

- Individuals who harass other attendees while attending the workshop or who are deemed incapable of obeying the specified rules of attendance.
- Individuals who are rendered incapable of participation during this study.
- Individuals suffering from a condition or disability that is deemed to preclude study participation. If there are difficulties with that determination, an individual may be barred from participation at the discretion of a physician.
- Individuals who are otherwise deemed ineligible by an investigator (or sub-investigator).
-

Note 1 The CIR scale score for the general public is a mean of 3.8 points (±1),[10] so the subjects in this study must have at least that score.

Recruitment and allocation

Subject recruitment

In a previous study, hoarding participants were grouped into three classes: "non-comorbid" hoarding (42%), hoarding with depression (42%), and hoarding with depression and inattention (16%). [4] We considered recruitment so the participants would be representative of a society that includes hoarding behavior. We will recruit participants in several ways, including notices posted at Teikyo University; and open recruitment from notices at an ADHD mutual self-help group, society, helper station and visiting care station. We will also put up posters at helper stations, visiting care stations, community pharmacies, and a university, high school and junior high school in northwest Tokyo. Participants will also be recruited from among the general public via a research recruitment website, an events firm, publicity information sent to cleaning companies, a non-profit organization providing support for people with ADHD and a notice at the university.

Applicants will visit Teikyo University to receive an oral and written explanation of the purpose of the study. After initial agreement, and assessment using the questionnaire, we will determine eligibility.

Subject assignment

We will provide potential participants with an overview of the study. They will be asked to complete a preliminary study to assess their eligibility. When potential subjects are verified as eligible, they will be asked to attend an information session about the trial. At the session, if they give their full consent, they will be enrolled. Each subject will be randomly assigned to either the intervention or control group. Subjects will be enrolled until the required sample size (30 subjects per group) is obtained. We consider that the effects of lifestyle education need several months to show progress, and many studies use 6 months or longer [22,23]. We therefore set a term of 7 months by considering 6 months after the intervention begins as the observation period in this study.

Randomization and blinding

A sealed envelope method is used for random allocation. This is based on a permuted block method with a block size of four and a random number table created before the start of the study. Each pre-sealed envelope, prepared by a person not involved with the research, contains a random number. Participants publicly choose one envelope and open it at the place of the session meeting, where consent is obtained.

Subject are randomly assigned to either the intervention or control group, and then a professional organizing and cleaning service is randomly assigned, also via random number. The group assignment is not based on participants' previous experiences with organizing and cleaning.

Because of the open-label design, the participants know whether they are assigned to the

For peer review only - http://bmjopen.bmj.com/site/about/guidelines.xhtml

intervention or control group. However, this information is blinded for the professional organizing and cleaning service to avoid risk of bias.
Effectiveness of the intervention will be determined using surveys completed by the participants, and sent directly to the Study Office, and a photograph of the room to assess room cleanliness or clutter. The home organizing and cleaning services will not be able to see the results for individual subjects.

Interventions

Intervention group

This group will attend a workshop program, undergo inspections of the cleanliness or clutter of their rooms, and receive visits from an organizing and cleaning service. The workshop will consist of four sessions. Room cleanliness or clutter will be inspected six times. The first inspection will take place once eligibility is verified and consent is obtained. The second will take place before the start of the study. Others will take place 4 weeks, 2 months, 4 months and 7 months after the start of the study (see Figure 1).

Control group

The control group will receive an initial inspection of room cleanliness or clutter once eligibility is verified and consent obtained. The second inspection will take place before the intervention group starts the workshop. Further inspections will take place 4 weeks, 2 months, 4 months, and 7 months after the start of the study. Members of the control group who wish to attend the workshop may do so after the study period ends.

The content of the workshop

The workshop's purpose is to improve participant behavior and develop habits of decluttering and organizing. It starts by covering the relationship between physical and mental health and the state of the living space. It then discusses participants' ideal life. A study representative has developed a workshop program based on the material from the Japan Association of Life Organizers and Edison Club (a nonprofit organization supporting patients with ADHD, their families, and teachers). A public health nurse and an organizer deliver four lectures, with the former leading 30 minutes and the latter 1.5 hours. (see Table 2).

Table 2 Syllabus for "Workshop on Decluttering and Organizing"

1st (2_hours)	<Reduce><br>·Orientation and goal setting. (public health nurse)<br>·Understanding the importance of working as a group. (public health nurse)<br>·Three fundamentals for healthy and comfortable living (reduce, organize, maintain). (organizer)<br>·Organizing in small steps: around your desk and your bed. (organizer)<br>·Explanation of homework. (public health nurse)
2nd (2_hours)	<Organize><br>·Review of previous session (public health nurse)<br>·The relation between decluttering and organizing and health (fall prevention, allergic disease) (public health nurse)<br>·Organizing in small steps: the kitchen (organizer)<br>·Explanation of homework. (public health nurse)
3rd (2_hours)	< Review ><br>·Review of previous session (public health nurse)<br>·Solutions for people who have difficulty discarding things and buy (or receive) unnecessary things (public health nurse)<br>·Organizing in small steps: a place to relax(organizer)<br>·Explanation of homework. (public health nurse)
4th	< Maintain>

For peer review only - http://bmjopen.bmj.com/site/about/guidelines.xhtml

(2_hours)	·Review of previous session (public health nurse)<br>·Organizing in small steps: closets and wardrobes (organizer)<br>·Schedule of questionnaire submission (public health nurse)

Outcome measures and subject's characteristics

Primary outcome

The primary outcome is the score on the Japanese version of the Saving Inventory-Revised (SI-R). The SI-R score is an index used to gauge the difficulties posed by a hoarding disorder (a higher score indicates a greater tendency to hoard). It measures mental anguish caused by hoarding, and the impacts of hoarding on life. A higher score indicates a greater tendency to hoard.[20,21] There is presently no Japanese version of the Child Saving Inventory [24]. It appeared reasonable to use the SI-R for those under age 18 based on the pre-test; we therefore used the SI-R in this study. The primary effect size is the difference changes in SI-R score from baseline to 7 months between the two groups. The hypothesis of this study is that the mean SI-R score of the intervention group will decrease more than that of the control group.

Secondary outcomes

Secondary outcomes include:

- Score on the Clutter Image Rating (CIR) scale, which measures clutter using photographs of rooms (a higher score indicates more clutter).[25,26]
- Score on the Japanese version of the Rosenberg Self-Esteem Scale (RSES).[27] This measures the degree of self-respect and the value placed on the self. A high score shows stronger feelings of self-esteem.
- Score on the Roles of Private Space Scale (RPSS). This linear measure observes the function, the necessity and securement level of private space. [28]

These outcomes will be used to examine the amount of decluttering and organizing, including frequency of cleaning and number of visitors in a week. They will allow the researchers to assess the state of subjects' living spaces and their ability to declutter and organize as a result of the workshop.

Subject characteristics

During the first meeting, participants will provide information about their gender, age, co-morbidities, previous history, medication, number of roommates/family members in their household, number and size of living spaces, how often their living space is organized and tidied up, number of visitors to their living space in the previous week, whether they have difficulty decluttering and organizing, and are living with a roommate or family members, and aspects of social status such as occupation, marital status, educational background, and work pattern (whether they are working on a shift basis).

Statistical Analysis

Sample size

A previous study divided 46 patients with a hoarding disorder (mean age: 53.9 years) into two groups. One group of 23 subjects was scheduled for 26 sessions of cognitive behavioral therapy approximately on a weekly basis. The second group of 23 subjects had yet to undergo cognitive behavioral therapy.[16] A comparison of the two groups indicated that subjects who had completed the 12th session of cognitive behavioral therapy had a 10-point drop in their SI-R score. Around 10 points were improved upon using a pre-test on the general population. A meta-analysis in another study indicated that intervention was more effective in younger individuals than older ones. This study therefore assumed a potential 10-point improvement in SI-R score.[16] Previous results indicated that the SI-R score had a normal distribution and SD was 13.0.[19] The sample size was therefore calculated as 28 per group under the assumptions of a significance level of 5%

For peer review only

For peer review only - http://bmjopen.bmj.com/site/about/guidelines.xhtml

(two-tailed), a power of 80%, and an effect size of 0.75. Considering the likely drop-out rate, we set the sample size needed for this trial as 60 subjects in total, 30 in each group.

Statistical Analysis

Analyses will be conducted under the intention-to-treat (ITT) principle. The main target set for analysis is the full analysis set (FAS). Missing data will be permutated under the assumption of missing at random (MAR) following the last observation carried forward (LOCF) principle. A sensitivity analysis will be performed using the multiple imputation (MI) method. The secondary target set for the analyses will be the per protocol set (PPS).

Summary statistics (maximum, median, minimum, mean, and standard deviation) will be calculated for all continuous data, and frequency and proportion for categorical data.

The differences in effect sizes between the two groups will be examined using a t-test. Differences will be assessed in a general linear model by adjusting for gender, age and marital status at the start of the study, and outcome measure at the baseline. Crude and adjusted odds ratios will be calculated for binary variables, and the two groups will be compared using logistic regression analysis. As secondary analysis, mixed-effects random-effects models will be used as a longitudinal data analysis. The significance level for testing will be set at $p < 0.05$ (two-tailed).

Timeframe of the study

Participants were registered between January and May 2016. Eight workshops have been run between 3 February and 11 June 2016. Data are currently being collected.

Data management

Personal information obtained in this study will be coded. Participant files will be stored in numerical order in a secure but accessible place and manner. Data will be anonymized before analysis. Data will be kept on a password-protected computer with limited access. The study organizer will keep all documents related to this trial in a locked cabinet. Five years after the study is presented, or three years after the final publication of the results (whichever is later), all documents will be disposed of using a document destruction service contracted by the University.

Conditions for discontinuation of this study and actions in that event

When significant information about the safety or effectiveness of the intervention is obtained, the study organizer will determine whether to continue the study.

Treatment of subjects after this study is conducted

If the intervention group is performing significantly better than the control group at the end of the study, the control group will be informed. Members of the control group who wish to do so may then attend the workshop after the conclusion of this study.

Monitoring

Information on when the study begins, the conduct of the study (sample size), ethical considerations that have been made, the occurrence of any detrimental or adverse events, the results of the study, and registration of the study with a public database will be submitted to the ethics committee in an annual interim report. A report will also be submitted to the ethics committee upon the conclusion of the study, and when the final results are presented.

Follow-up of adverse events

This study involves attending a workshop program to help people declutter and organize, and receiving visits from a professional organizing and cleaning service. There is little likelihood of any health hazards to the subjects. If any serious adverse events occur, they

For peer review only - http://bmjopen.bmj.com/site/about/guidelines.xhtml

will be reported in line with the standard operating procedure on Reporting Serious Adverse Events in Clinical Research.

Ethics and dissemination

The study protocol has been approved by the Medical Ethical Committee of Teikyo University (No. 15-065). The findings will be disseminated widely through peer-reviewed publication and conference presentations.

Conflicts of interest

Based on a random number table, home organizing and cleaning services will be randomly assigned to clean the subjects' living space (the organizers will be blind to whether they are in the control or intervention group), to maintain the fairness of this study. Effectiveness of the intervention will be determined using a survey completed by the subject and sent directly to the Study Office, together with a photograph to assist determination of room cleanliness or clutter. Home organizing and cleaning services will not be able to see any results for individual subjects.

DISCUSSION

One aim of this study is to assess the effect of an intervention (a workshop) on the state of living spaces and the quality of life of younger people who have difficulty decluttering and organizing and who therefore have a cluttered living space. If the approach is found to be effective, then the findings of this study will be widely publicized and the workshop materials will be made widely available, so that it can be conducted by others, including administrative bodies and professional organizers and cleaners. This should improve the quality of life and help to prevent a hoarding disorder in younger people who have difficulty decluttering and organizing.

This study has strengths and weaknesses. Blinding is not possible, because of the type of intervention. It is impossible to create a convincing placebo for the workshop on decluttering and organizing, but this lack of blinding may affect the results. As far as possible, subjects need to have an experience equivalent to undergoing training from a professional organizing and cleaning service.

Acknowledgments

We are grateful for advice from Yukiko Mochizuki, Yuka Nojiri, and Mihoko Shimozono about preparing a study protocol.

The text for the workshop was prepared with help from Keiko Takayama and Mayumi Takahara.

Contributors

YA conceived the study. KY and NA participated in the development of the protocol. MS and YN contributed to study conception and design for the education programme. All authors prepared and revised the manuscript, including relevant scientific content. All authors approved the final version of the manuscript.

Funding

This work was supported by JSPS KAKENHI Grant Number JP26671045(2014–2017) Grant-in-Aid for Scientific Research on "The state of young people who chronically fail to organize or tidy up and development of a program for effective intervention" (Challenging Exploratory Research, Study Organizer: Yasuko Aso, grant no. 26671045).

Competing interests statement

No financial assistance or free services will be received from any company or

For peer review only - http://bmjopen.bmj.com/site/about/guidelines.xhtml

organization during this study. The researchers have no equity, such as stock, in any of the companies involved, and are not involved in their management, nor have the researchers received any services, free goods or pharmaceuticals, or discounts from those companies.

Home organizing and cleaning services will, based on a random number table, be randomly assigned to clean a subject's living space to maintain the fairness of this study. Effectiveness of the intervention will be determined based on a survey sent by the subject directly to the Study Office and a determination of room cleanliness or clutter based on photographs. Home organizing and cleaning services will not be able to view results for individual subjects.

Subjects' consent

Obtained.

Ethics approval

This trial has been approved by Teikyo University Review Board15-065
Protocol version 1.Issue date 11 November 2015, Authors: *YA, KY,NA.*

Data sharing statement

The informed consent documents are available on request.

REFERENCES

1. American Psychiatric Association. Diagnostic and statistical manual of mental disorder (DSM). Washington, DC: American Psychiatric Association 1994:143–7.
2. Tolin DF, Frost RO, Steketee G, et al. The economic and social burden of compulsive hoarding. *Psychiatry Res* 2008;160(2):200–11.
3. Kishi E, Nojiri Y, Yonezawa J, et al. An analysis of forms of intervention by nurses at comprehensive community support centers in cases of self-neglect. *Journal of the Japan Academy for the Prevention of Elder Abuse* 2014;10(1):106–20.
4. Hall BJ, Tolin DF, Frost RO, et al. An exploration of comorbid symptoms and clinical correlates of clinically significant hoarding symptoms. *Depress Anxiety* 2013;30(1):67–76.
5. Frank H, Stewart E, Walther M, et al. A. Hoarding behavior among young children with obsessive–compulsive disorder. *Journal of obsessive-compulsive and related disorders* 2014; 3(1): 6-11.
6. Samuels JF, Bienvenu OJ, Riddle MA, et al. Hoarding in obsessive compulsive disorder: results from a case-control study. Behav Res Ther 2002 May;40(5):517–528. [PubMed]
7. Frost RO, Krause MS, Steketee G. Hoarding and obsessive-compulsive symptoms. Behavior Modification 1996;20:116–132. [PubMed]
8. Rasmussen SA, Eisen JL. Clinical features and phenomenology of obsessive compulsive disorder. *Psychiatric Annals* 1989;19:67–73.
9. Sobin C, Blundell ML, Weiller F, et al. Evidence of a schizotypy subtype in OCD. *Journal of Psychiatric Research* 2000;34:15–24. [PubMed]
10. Saxena S, Maidment KM, Vapnik T, et al. Obsessive-compulsive hoarding: symptom severity and response to multimodal treatment. *J Clin Psychiatry* 2002 Jan;63(1):21–27. [PubMed]
11. Timpano K R, Raines A M, Shaw A M, et al. Effects of a brief anxiety sensitivity reduction intervention on obsessive compulsive spectrum symptoms in a young adult sample. *Journal of Psychiatric Research* 2016;83, 8-15.
12. Grisham JR, Frost RO, Steketee G, et al. Age of onset of compulsive hoarding. *J Anxiety Disord* 2006;20(5):675–86.

For peer review only - http://bmjopen.bmj.com/site/about/guidelines.xhtml

13. Eckfield MB, Wallhagen MI. The synergistic effect of growing older with hoarding behaviors. *Clin Nurs Res* 2013;22(4):475–91.
14. Nordsletten AE. Epidemiology of hoarding disorder. *Br J Psychiatry* 2013;203:445–52.
15. Tolin DF, Frost RO, Steketee G. Cognitive behavioral therapy for hoarding disorder: A meta-analysis. *Depress Anxiety* 2015;32(3):158–66.
16. Steketee G, Frost R O, Tolin D F, et al. Waitlist - controlled trial of cognitive behavior therapy for hoarding disorder. *Depression and anxiety* 2010;27(5):476-484.
17. Frost R O, Ruby D, Shuer L J. The buried in treasures workshop: waitlist control trial of facilitated support groups for hoarding. *Behaviour research and therapy* 2012; 50(11), 661-667.
18. Katadukeha nigatedesuka? [Do you have difficulty organizing your home?] Asahi newspaper 2013 Mar 30,;sect.b 10(col.1).
19. Aso Y, Masashige S, Yuka N, et al. Attitudes of undergraduates studying nursing or welfare work towards organizing and tidying up and the state of their living spaces. [abstract]. Nagasaki; Japan, The 74th Annual Meeting of the Japanese Society of Public Health 2015;p294.
20. Aki T, Ken-ichi K, Toko I, et al. A consideration of clinical characteristics of non-clinical hoarding among Japanese adolescents. *Japanese Society of Anxiety and Related Disorder* 2015;6(2):72–85.
21. Frost RO, Steketee G, Grisham JR. Measurement of compulsive hoarding: saving inventory-revised. *Behav Res Ther* 2004;42(10):1163–82.
22. Kazue Y, Toshiro T. "Effects of lifestyle modification on metabolic syndrome: a systematic review and meta-analysis. *BMC medicine* 2012; 1
23. Anderson L M, Quinn T A, Glanz K, et al. The effectiveness of worksite nutrition and physical activity interventions for controlling employee overweight and obesity: a systematic review. *American journal of preventive medicine* 2009;37(4):340-357.
24. Storch E A, Muroff J, Lewin A B, et al. Development and preliminary psychometric evaluation of the Children's Saving Inventory. *Child Psychiatry & Human Development* 2011; 42(2): 166-182.
25. Frost RO, Steketee G. Compulsive Hoarding and Acquiring: Therapist Guide. Japanese edition. Toko I, translator. Tokyo: Kanekoshobou 2013:170–3.
26. Frost RO, Steketee G, Tolin DF, et al. Development and validation of the Clutter Image Rating. *J Psychopathol Behav Assess* 2008;30(3):193–203.
27. MarikoY, Yutaka M, Yukiko Y. The Structure of Perceived Aspects of Self. *Japanese Association of Educational Psychology* 1982;30(1):64–8.
28. Tomari S, Yoshida F. Psychological review of meanings of private space and its privacy research and proposal for functions: new models. *Tsukuba Psychological Research* 1998;20:173–90.

FIGURE TITLES

Figure 1. Trial design

For peer review only - http://bmjopen.bmj.com/site/about/guidelines.xhtml

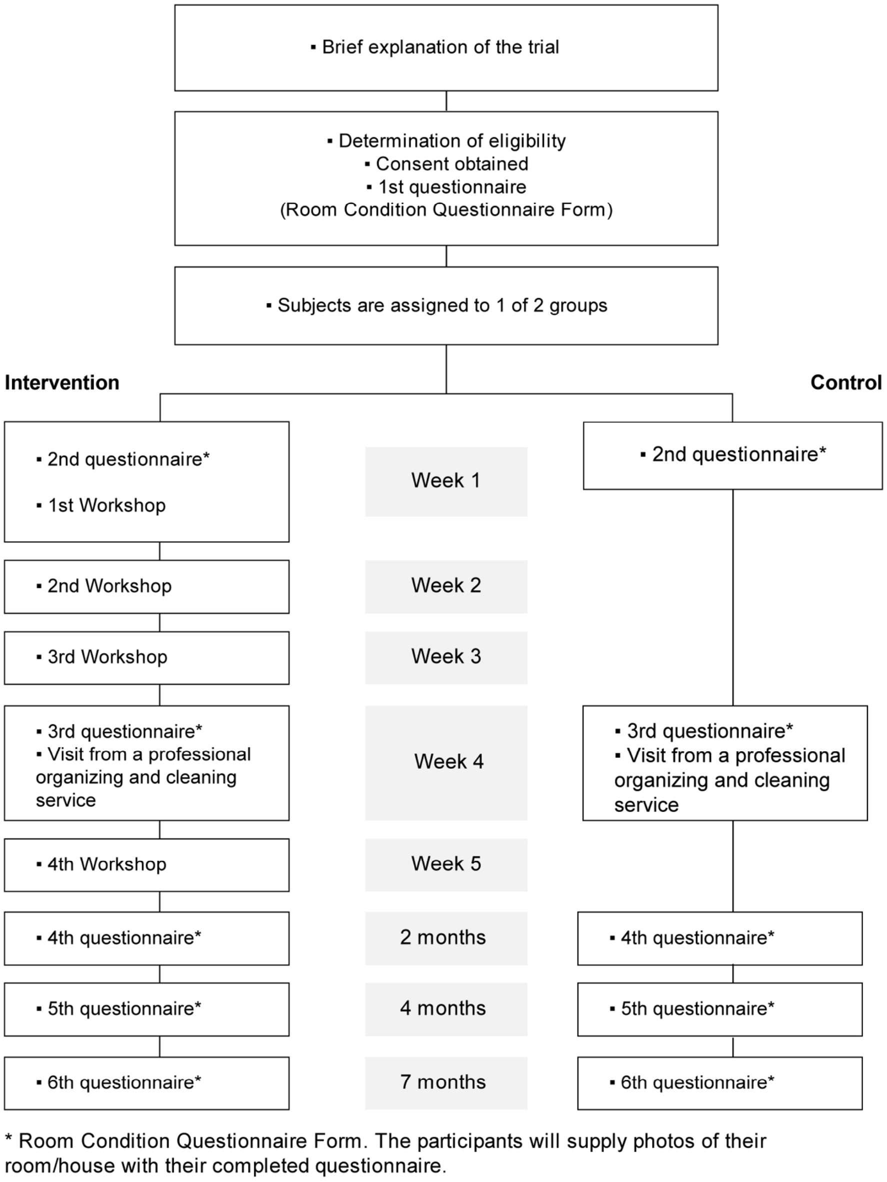

Figure 1. Trial design

140x177mm (300 x 300 DPI)

SPIRIT

STANDARD PROTOCOL ITEMS: RECOMMENDATIONS FOR INTERVENTIONAL TRIALS

SPIRIT 2013 Checklist: Recommended items to address in a clinical trial protocol and related documents*

Section/item	Item No	Description	Addressed on page number
**Administrative information**			
Title	1	Descriptive title identifying the study design, population, interventions, and, if applicable, trial acronym	1
Trial registration	2a	Trial identifier and registry name. If not yet registered, name of intended registry	2
	2b	All items from the World Health Organization Trial Registration Data Set	All items were covered.
Protocol version	3	Date and version identifier	10
Funding	4	Sources and types of financial, material, and other support	9
Roles and responsibilities	5a	Names, affiliations, and roles of protocol contributors	9, 1
	5b	Name and contact information for the trial sponsor	not applicable
	5c	Role of study sponsor and funders, if any, in study design; collection, management, analysis, and interpretation of data; writing of the report; and the decision to submit the report for publication, including whether they will have ultimate authority over any of these activities	not applicable as stated at P9（Competing interests statement）

For peer review only - http://bmjopen.bmj.com/site/about/guidelines.xhtml

	5d	Composition, roles, and responsibilities of the coordinating centre, steering committee, endpoint adjudication committee, data management team, and other individuals or groups overseeing the trial, if applicable (see Item 21a for data monitoring committee)	Study Protocol(p9-10)
**Introduction**			
Background and rationale	6a	Description of research question and justification for undertaking the trial, including summary of relevant studies (published and unpublished) examining benefits and harms for each intervention	3 -4
	6b	Explanation for choice of comparators	6
Objectives	7	Specific objectives or hypotheses	4
Trial design	8	Description of trial design including type of trial (eg, parallel group, crossover, factorial, single group), allocation ratio, and framework (eg, superiority, equivalence, noninferiority, exploratory)	4
**Methods: Participants, interventions, and outcomes**			
Study setting	9	Description of study settings (eg, community clinic, academic hospital) and list of countries where data will be collected. Reference to where list of study sites can be obtained	5
Eligibility criteria	10	Inclusion and exclusion criteria for participants. If applicable, eligibility criteria for study centres and individuals who will perform the interventions (eg, surgeons, psychotherapists)	4, Table 1
Interventions	11a	Interventions for each group with sufficient detail to allow replication, including how and when they will be administered	6,Table 2
	11b	Criteria for discontinuing or modifying allocated interventions for a given trial participant (eg, drug dose change in response to harms, participant request, or improving/worsening disease)	8
	11c	Strategies to improve adherence to intervention protocols, and any procedures for monitoring adherence (eg, drug tablet return, laboratory tests)	Exhibit 1-1 Interference Group Manual

For peer review only - http://bmjopen.bmj.com/site/about/guidelines.xhtml

	11d	Relevant concomitant care and interventions that are permitted or prohibited during the trial	Exhibit2 Decluttering and Organizing Class Text
Outcomes	12	Primary, secondary, and other outcomes, including the specific measurement variable (eg, systolic blood pressure), analysis metric (eg, change from baseline, final value, time to event), method of aggregation (eg, median, proportion), and time point for each outcome. Explanation of the clinical relevance of chosen efficacy and harm outcomes is strongly recommended	7
Participant timeline	13	Time schedule of enrolment, interventions (including any run-ins and washouts), assessments, and visits for participants. A schematic diagram is highly recommended (see Figure)	Figure 1, 6
Sample size	14	Estimated number of participants needed to achieve study objectives and how it was determined, including clinical and statistical assumptions supporting any sample size calculations	7
Recruitment	15	Strategies for achieving adequate participant enrolment to reach target sample size	5
**Methods: Assignment of interventions (for controlled trials)**			
Allocation:			
Sequence generation	16a	Method of generating the allocation sequence (eg, computer-generated random numbers), and list of any factors for stratification. To reduce predictability of a random sequence, details of any planned restriction (eg, blocking) should be provided in a separate document that is unavailable to those who enrol participants or assign interventions	5
Allocation concealment mechanism	16b	Mechanism of implementing the allocation sequence (eg, central telephone; sequentially numbered, opaque, sealed envelopes), describing any steps to conceal the sequence until interventions are assigned	5
Implementation	16c	Who will generate the allocation sequence, who will enrol participants, and who will assign participants to interventions	5
Blinding (masking)	17a	Who will be blinded after assignment to interventions (eg, trial participants, care providers, outcome assessors, data analysts), and how	5-6

For peer review only - http://bmjopen.bmj.com/site/about/guidelines.xhtml

	17b	If blinded, circumstances under which unblinding is permissible, and procedure for revealing a participant's allocated intervention during the trial	Not applicable

Methods: Data collection, management, and analysis

Data collection methods	18a	Plans for assessment and collection of outcome, baseline, and other trial data, including any related processes to promote data quality (eg, duplicate measurements, training of assessors) and a description of study instruments (eg, questionnaires, laboratory tests) along with their reliability and validity, if known. Reference to where data collection forms can be found, if not in the protocol	Exhibit 1-1 Interference Group Manual、 Exhibit 1-2 Control Group Manual、
	18b	Plans to promote participant retention and complete follow-up, including list of any outcome data to be collected for participants who discontinue or deviate from intervention protocols	Exhibit 1-1 Interference Group Manual 、Exhibit 1-2 Control Group Manual
Data management	19	Plans for data entry, coding, security, and storage, including any related processes to promote data quality (eg, double data entry; range checks for data values). Reference to where details of data management procedures can be found, if not in the protocol	8
Statistical methods	20a	Statistical methods for analysing primary and secondary outcomes. Reference to where other details of the statistical analysis plan can be found, if not in the protocol	7- 8
	20b	Methods for any additional analyses (eg, subgroup and adjusted analyses)	8
	20c	Definition of analysis population relating to protocol non-adherence (eg, as randomised analysis), and any statistical methods to handle missing data (eg, multiple imputation)	8

Methods: Monitoring

Data monitoring	21a	Composition of data monitoring committee (DMC); summary of its role and reporting structure; statement of whether it is independent from the sponsor and competing interests; and reference to where further details about its charter can be found, if not in the protocol. Alternatively, an explanation of why a DMC is not needed	8
	21b	Description of any interim analyses and stopping guidelines, including who will have access to these interim results and make the final decision to terminate the trial	Not applicable
Harms	22	Plans for collecting, assessing, reporting, and managing solicited and spontaneously reported adverse events and other unintended effects of trial interventions or trial conduct	8
Auditing	23	Frequency and procedures for auditing trial conduct, if any, and whether the process will be independent from investigators and the sponsor	8
**Ethics and dissemination**			
Research ethics approval	24	Plans for seeking research ethics committee/institutional review board (REC/IRB) approval	10
Protocol amendments	25	Plans for communicating important protocol modifications (eg, changes to eligibility criteria, outcomes, analyses) to relevant parties (eg, investigators, REC/IRBs, trial participants, trial registries, journals, regulators)	Study Protocol P14
Consent or assent	26a	Who will obtain informed consent or assent from potential trial participants or authorised surrogates, and how (see Item 32)	Exhibit 7 -10
	26b	Additional consent provisions for collection and use of participant data and biological specimens in ancillary studies, if applicable	10
Confidentiality	27	How personal information about potential and enrolled participants will be collected, shared, and maintained in order to protect confidentiality before, during, and after the trial	8
Declaration of interests	28	Financial and other competing interests for principal investigators for the overall trial and each study site	9-10
Access to data	29	Statement of who will have access to the final trial dataset, and disclosure of contractual agreements that limit such access for investigators	10

For peer review only - http://bmjopen.bmj.com/site/about/guidelines.xhtml

Ancillary and post-trial care	30	Provisions, if any, for ancillary and post-trial care, and for compensation to those who suffer harm from trial participation	8
Dissemination policy	31a	Plans for investigators and sponsor to communicate trial results to participants, healthcare professionals, the public, and other relevant groups (eg, via publication, reporting in results databases, or other data sharing arrangements), including any publication restrictions	8-9 , Study Protocol P14, Exhibit 7(p5), Exhibit 7(p4),
	31b	Authorship eligibility guidelines and any intended use of professional writers	9
	31c	Plans, if any, for granting public access to the full protocol, participant-level dataset, and statistical code	10
**Appendices**			
Informed consent materials	32	Model consent form and other related documentation given to participants and authorised surrogates	Exhibit 7 - 10
Biological specimens	33	Plans for collection, laboratory evaluation, and storage of biological specimens for genetic or molecular analysis in the current trial and for future use in ancillary studies, if applicable	Not Applicable

*It is strongly recommended that this checklist be read in conjunction with the SPIRIT 2013 Explanation & Elaboration for important clarification on the items. Amendments to the protocol should be tracked and dated. The SPIRIT checklist is copyrighted by the SPIRIT Group under the Creative Commons "Attribution-NonCommercial-NoDerivs 3.0 Unported" license.

For peer review only

For peer review only - http://bmjopen.bmj.com/site/about/guidelines.xhtml

Effectiveness of “Workshop on Decluttering and Organizing” program for teens and middle-aged adults with difficulty decluttering: a study protocol of an open-label, randomized, parallel-group, superiority trial in Japan.

Journal:	*BMJ Open*
Manuscript ID	bmjopen-2016-014687.R2
Article Type:	Protocol
Date Submitted by the Author:	26-Feb-2017
Complete List of Authors:	Aso, Yasuko; Teikyo University, Department of Nursing, Faculty of Medical Technology<br>Yamaoka, Kazue; Teikyo University, Graduate School of Public Health<br>Nemoto, Asuka; Teikyo University, Graduate School of Public Health<br>Naganuma, Yuki; Teikyo University, Department of Nursing, Faculty of Medical Technology<br>Saito, Masashige; Nihon Fukushi University, Department of Social Welfare
**Primary Subject Heading**:	Public health
Secondary Subject Heading:	Nursing, Mental health, Health services research
Keywords:	Saving Inventory–Revised, Workshop on Decluttering and Organizing, Study protocol, Randomized controlled trial, Teens and midd, Protocols & guidelines < HEALTH SERVICES ADMINISTRATION & MANAGEMENT, PUBLIC HEALTH

SCHOLARONE™ Manuscripts

Effectiveness of "Workshop on Decluttering and Organizing" program for teens and middle-aged adults with difficulty decluttering: a study protocol of an open-label, randomized, parallel-group, superiority trial in Japan.

Yasuko Aso[1,2], Kazue Yamaoka[2], Asuka Nemoto[2], Yuki Naganuma[1], Masashige Saito[3]

Author Affiliations
[1] Department of Nursing, Faculty of Medical Technology, Teikyo University, Tokyo, Japan
[2] Teikyo University Graduate School of Public Health, Tokyo, Japan
[3] Department of Social Welfare, Faculty of Social Welfare, Nihon Fukushi University, Aichi, Japan

Correspondence to
Yasuko Aso.
Postal address: 2-1-11 Kaga, Itabashi-ku, Tokyo 173-8605
Email: asou@med.teikyo-u.ac.jp
Tel: +81-3-3964-1211 Ext.46115

Key words: Saving Inventory–Revised, Workshop on Decluttering and Organizing, Study protocol, Randomized controlled trial, Teens and middle-aged adults, Effectiveness

Words 3805

For peer review only

Abstract

Introduction: Hoarding disorder can cause problems with work performance, personal hygiene, health, and well-being. The disorder is a growing social problem in Japan. Having difficulty discarding rubbish, decluttering and organizing can signal a future hoarding disorder, and early intervention is important. We developed an educational workshop on decluttering and organizing for teens and adults with difficulty organizing. The objective of this study is to evaluate the effectiveness of the workshop for reducing clutter and improving quality of life among younger people with difficulty decluttering and organizing.

Methods and analysis: An open-label, parallel-group randomized controlled trial will be conducted among volunteers aged 12–55 years with mild difficulty decluttering and organizing. The intervention group will attend the workshop and receive a visit from a professional cleaning company to declutter their living space. The control group will have only the latter. The primary outcome will be the score on the Japanese version of the Saving Inventory–Revised. Secondary outcomes will be scores on the Clutter Image Rating Scale, the Japanese version of the Rosenberg Self-Esteem Scale, and the Roles of Private Space Scale. The results will be examined for differences between the two groups in changes from baseline to 7 months. We will examine crude effects and adjust for gender, age and marital status, using a general linear model for continuous variables and a logistic regression model for dichotomous variables. Sample size was calculated assuming a significance level of 5% (two-tailed), a power of 80%, and an effect size of 0.75. In total, 60 subjects (30 in each group) will be required.

Ethics and dissemination: The study protocol has been approved by the Medical Ethical Committee of Teikyo University (No. 15-065). The findings will be disseminated widely through peer-reviewed publication and conference presentations.

Trial registration number: UMIN000020568. Issue Date: 16 January 2016

Strengths and limitations of this study

- The study will be an open-label, parallel-group randomized controlled trial and, to our knowledge, is the first such study on this subject in Japan.
- It will test a simple but feasible intervention for those having difficulty decluttering and organizing.
- The outcomes measures are clearly set using reliable scales.
- The test is adequately powered to detect effects on the outcomes.
- Blinding is not possible because of the type of intervention, which may affect the results.

For peer review only

For peer review only - http://bmjopen.bmj.com/site/about/guidelines.xhtml

INTRODUCTION

A hoarding disorder can cause problems with work performance, personal hygiene, health, and wellbeing. It occasionally results in a house with great amounts of garbage, and with key symptoms including difficulty discarding, excessive clutter, and excessive acquisition. [1, 2] This poses a number of dangers, such as the potential for fire and falls. The trash or clutter also affects an individual's overall quality of life. [3] This issue is a growing social problem in Japan.

An individual living in such conditions can become isolated from the rest of society. This can be particularly the case for older people. [4] The unsanitary living conditions will eventually become a public health problem affecting neighbors. Hoarding disorder is therefore not only an individual problem but also a problem for society, and a practical solution is warranted from a public health point of view.

Hoarding disorder is often accompanied by psychiatric disorders such as attention deficit hyperactivity disorder (ADHD), depression, or obsessive–compulsive disorder (OCD) [5-10]. Its relationship with distress tolerance and anxiety sensitivity has also been noted in a sample of young adults. [11] Signs of hoarding appear in early adolescence, and its severity increases with age: age 138 [12] or 11–15 [13]. Many sufferers report symptom onset before age 20 [13], and symptoms tend to worsen past age 55. [12] These points suggest prevention in the young generation is important and we therefore targeted those aged 12–55 years old.

Concerning the United States and Europe, a previous study estimated that the incidence of hoarding disorder is 1.5%.[14] Another study reported that cognitive behavioral therapy, either individually or in groups, and home visits are effective at assisting patients with hoarding disorder. However, these cognitive behavioral therapies needed relatively long intervention periods and the subject samples were older people [15-17].

However, according to the investigation of the effectiveness of a brief, anxiety sensitivity-focused on a single-session intervention for reducing obsessive–compulsive (OC) spectrum and hoarding among a young adult sample's symptoms. The Anxiety Sensitivity Education and Reduction Training (ASERT) group demonstrated greater reductions in OC symptoms immediately following treatment, and continued to make gains across the follow-up period. In contrast, a hypothesis regarding similar effects of an intervention on reduction of hoarding symptoms was not supported [11]. Therefore, there is a need to identify new avenues through which to develop additional interventions.

Aging of society is progressing in Japan, and it is important to prevent hoarding that may be associated with those who display this behavior. Therefore, there is urgent need to establish a system of early intervention and support to prevent younger people who have difficulty decluttering and organizing from developing a hoarding disorder.

A previous study examined the state of living space of 450 undergraduates from two universities. [18] The results showed that younger people who had difficulty decluttering and organizing tended to live in cluttered or disorganized living spaces and to have low self-esteem. The study used the Japanese version of the Saving Inventory–Revised (SI-R).[19] This was developed in the United States[2] and translated into Japanese, and its reliability and validity have been verified.[2] The previous study's SI-R scores had a mean of 32.1 points (±13.0) and a normal distribution. SI-R score has been reported as high among people who said that they did not like to clean, [18] but many such people also want to improve the state of their living space. [18] There are various ways to learn how to clean effectively in Japan, including workshops, books and professional support, but it is not clear which method is most effective.

We have therefore developed an education program, of a series of workshops on decluttering and organizing, for teens and adults who find it difficult to organize. In the workshops, we emphasize the importance of a sense of values (such as necessity) that each person has for things. It also covers the state of the individual's living space and its

For peer review only - http://bmjopen.bmj.com/site/about/guidelines.xhtml

relationship with physical and mental health. The purpose of the workshop is to improve recognition of the importance of cleaning and to change behavior.

We have therefore developed an education program, of a series of workshops on decluttering and organizing, for teens and adults who find it difficult to organize. In the workshops, we will help identify what belongings a person truly needs. It also covers the state of the individual's living space and its relationship with physical and mental health. The purpose of the workshop is to improve recognition of the importance of cleaning and to change behavior.

This study examines behavior 7 months after the intervention, using a control group, and is designed to assess both changes in habits, and levels of stress. It is impossible to blind this type of intervention, so the study was made open label to minimize bias.

The aim of the study is to evaluate the effectiveness of the program in reducing clutter and improving the quality of life in teens and middle-aged adults who have difficulty decluttering and organizing, and who have a cluttered living space.

METHODS AND ANALYSIS

Study design

The design of this study is an open-label, parallel-group, stratified, randomized controlled trial (RCT). The aim of this trial is to investigate the effect of the workshop plus a professional organizing and cleaning service. We hope that the program will improve the quality of life and help to prevent a hoarding disorder in younger people who currently have difficulty decluttering and organizing. Participants will be involved for a period of 7 months. The design of the trial is shown in Figure 1. Based on previous experience, it is expected that the study will include around 90% of eligible participants. Perioperative protocols are standardized.

[Insert Figure 1 (Trial design) about here]

Participants

The study will look for volunteers aged between 12 and 55 with mild organization difficulties. They will need to meet all of the inclusion criteria below and not meet any of the exclusion criteria (see Table 1).

Table 1 Inclusion criteria and exclusion criteria

Inclusion criteria

- Individuals who have difficulty decluttering and organizing. (SI-R score≥30)
- Individuals with a total score of four points or more on the Japanese version of the Clutter Image Rating (CIR) scale [Note 1].
- Individuals who are responsible for decluttering and organizing their own rooms.
- Individuals with no planned relocations, extensive renovations of living space, or room changes (to allow comparison of the state of living spaces before and after the intervention).
- Individuals who are able to attend at least three workshop sessions and receive visits from an organizing and cleaning service.
- Individuals who responded in the questionnaire, "I want to organize my room according to my will".
- Individuals who can send photographs of their living spaces at the specified time, i.e. without delay.
- Individuals or a roommate/family member (the informed consent form for minors if the individual is under the age of 15) who consent in writing to their participation in the study, and to having a better organized and tidied living space. If the individual is a minor, consent must be provided by a guardian.
- Individuals between the ages of 12 and 55 at the start of the study.

For peer review only - http://bmjopen.bmj.com/site/about/guidelines.xhtml

Exclusion criteria

- Individuals who are unable to organize and tidy up their living spaces during the study period, for example:
  - ✓ Individuals with an illness or disability
  - ✓ Individuals who are not living at the registered residence or are physically unable to regularly organize and tidy up
- Individuals living in a space that is too messy (An organization expert and two cleaning experts judge the space and deem whether it can be sufficiently organized within about 3 hours.)
- Individuals who harass other attendees while attending the workshop or who are deemed incapable of obeying the specified rules of attendance.
- Individuals who are rendered incapable of participation during this study.
- Individuals suffering from a condition or disability that is deemed to preclude study participation. If there are difficulties with that determination, an individual may be barred from participation at the discretion of a physician.
- Individuals who are otherwise deemed ineligible by an investigator (or sub-investigator).
-

Note 1 The CIR scale score for the general public is a mean of 3.8 points (±1),[10] so the subjects in this study must have at least that score.

Recruitment and allocation

Subject recruitment

We will recruit participants in several ways, including notices posted at Teikyo University; and open recruitment from notices at an ADHD mutual self-help group, society, helper station and visiting care station. We will also put up posters at helper stations, visiting care stations, community pharmacies, and a university, high school and junior high school in northwest Tokyo. Participants will also be recruited from among the general public via a research recruitment website, an events firm, publicity information sent to cleaning companies, a non-profit organization providing support for people with ADHD and a notice at the university.

Applicants will visit Teikyo University to receive an oral and written explanation of the purpose of the study. After initial agreement, and assessment using the questionnaire, we will determine eligibility.

Subject assignment

We will provide potential participants with an overview of the study. They will be asked to complete a preliminary study to assess their eligibility. When potential subjects are verified as eligible, they will be asked to attend an information session about the trial. At the session, if they give their full consent, they will be enrolled. Each subject will be randomly assigned to either the intervention or control group. Subjects will be enrolled until the required sample size (30 subjects per group) is obtained. We consider that the effects of lifestyle education need several months to show progress, and many studies use 6 months or longer [20,21]. We therefore set a term of 7 months by considering 6 months after the intervention begins as the observation period in this study.

Randomization and blinding

A sealed envelope method is used for random allocation. This is based on a permuted block method with a block size of four and a random number table created before the start of the study. Each pre-sealed envelope, prepared by a person not involved with the research, contains a random number. Participants publicly choose one envelope and open it at the place of the session meeting, where consent is obtained.

For peer review only - http://bmjopen.bmj.com/site/about/guidelines.xhtml

Subject are randomly assigned to either the intervention or control group, and then a professional organizing and cleaning service is randomly assigned, also via random number. The group assignment is not based on participants' previous experiences with organizing and cleaning.

Because of the open-label design, the participants know whether they are assigned to the intervention or control group. However, this information is blinded for the professional organizing and cleaning service to avoid risk of bias.

Effectiveness of the intervention will be determined using surveys completed by the participants, and sent directly to the Study Office, and a photograph of the room to assess room cleanliness or clutter. The home organizing and cleaning services will not be able to see the results for individual subjects.

Interventions

Intervention group

This group will attend a workshop program. The expert of an organizing service will receive a visit from a professional cleaning company to declutter the living space. The workshop will consist of four sessions. Room cleanliness or clutter will be inspect six times. The first inspection will take place once eligibility is verified and consent is obtained. The second will take place before the start of the study. Others will take place 4 weeks, 2 months, 4 months and 7 months after the start of the study (see Figure 1).

Control group

The control group will receive an initial inspection of room cleanliness or clutter once eligibility is verified and consent obtained. The second inspection will take place before the intervention group starts the workshop. The control group will receive a visit from a professional cleaning company only to declutter the living spaces. Further inspections will take place 4 weeks, 2 months, 4 months, and 7 months after the start of the study. Members of the control group who wish to attend the workshop may do so after the study period ends.

Additionally, until the end of the research period 7 months after commencement of the study, no participants are allowed to directly request any cleaning service be done.

The content of the workshop

The workshop's purpose is to improve participant behavior and develop habits of decluttering and organizing. It starts by covering the relationship between physical and mental health and the state of the living space. It then discusses participants' ideal life. A study representative has developed a workshop program based on the material from the Japan Association of Life Organizers and Edison Club (a nonprofit organization supporting patients with ADHD, their families, and teachers). The textbook used in the workshop is based on a text used in elementary schools [22]; therefore, 12-year-olds should have no difficulty understanding the contents.

A public health nurse and an organizer deliver four lectures, with the former leading 30 minutes and the latter 1.5 hours. (See Table 2).

Table 2 Syllabus for "Workshop on Decluttering and Organizing"

1st (2_hours)	<Reduce><br>·Orientation and goal setting. (public health nurse)<br>·Understanding the importance of working as a group. (public health nurse)<br>·Three fundamentals for healthy and comfortable living (reduce, organize, maintain). (organizer)<br>·Organizing in small steps: around your desk and your bed. (organizer)<br>·Explanation of homework. (public health nurse)
2nd (2_hours)	<Organize><br>·Review of previous session (public health nurse)

For peer review only - http://bmjopen.bmj.com/site/about/guidelines.xhtml

For peer review only

	·The relation between decluttering and organizing and health (fall prevention, allergic disease) (public health nurse)<br>·Organizing in small steps: the kitchen (organizer)<br>·Explanation of homework. (public health nurse)
3rd (2_hours)	< Review ><br>·Review of previous session (public health nurse)<br>·Solutions for people who have difficulty discarding things and buy (or receive) unnecessary things (public health nurse)<br>·Organizing in small steps: a place to relax(organizer)<br>·Explanation of homework. (public health nurse)
4th (2_hours)	< Maintain><br>·Review of previous session (public health nurse)<br>·Organizing in small steps: closets and wardrobes (organizer)<br>·Schedule of questionnaire submission (public health nurse)

Outcome measures and subject's characteristics

Primary outcome

The primary outcome is the score on the Japanese version of the Saving Inventory-Revised (SI-R). The SI-R score is an index used to gauge the difficulties posed by a hoarding disorder (a higher score indicates a greater tendency to hoard). It measures mental anguish caused by hoarding, and the impacts of hoarding on life. A higher score indicates a greater tendency to hoard. [2, 19] There is presently no Japanese version of the Child Saving Inventory. [23] The academic ability of Japanese teenagers, however, ranks among the top in the world. [24] Only 292 Japanese *kanji* characters are used for the Japanese version of the SI-R. And most of these *kanji* have already been learned in elementary school. In a pilot study on elementary school sixth graders, no comprehension problems were encountered. (Unpublished).

The primary effect size is the difference changes in SI-R score from baseline to 7 months between the two groups. The hypothesis of this study is that the mean SI-R score of the intervention group will decrease more than that of the control group.

Secondary outcomes

Secondary outcomes include:

- Score on the Clutter Image Rating (CIR) scale, which measures clutter using photographs of rooms (a higher score indicates more clutter).[25,26]
- Score on the Japanese version of the Rosenberg Self-Esteem Scale (RSES). [27] This measures the degree of self-respect and the value placed on the self. A high score shows stronger feelings of self-esteem.
- Score on the Roles of Private Space Scale (RPSS). This linear measure observes the function, the necessity and securement level of private space. [28]

These outcomes will be use to examine the amount of decluttering and organizing, including frequency of cleaning and number of visitors in a week. They will allow the researchers to assess the state of subjects' living spaces and their ability to declutter and organize because of the workshop.

Subject characteristics

During the first meeting, participants will provide information about their gender, age, co-morbidities, previous history, medication, number of roommates/family members in their household, number and size of living spaces, how often their living space is organized and tidied up, number of visitors to their living space in the previous week, whether they have difficulty decluttering and organizing, and are living with a roommate or family members, and aspects of social status such as occupation, marital status, educational background, and work pattern (whether they are working on a shift basis).

For peer review only - http://bmjopen.bmj.com/site/about/guidelines.xhtml

Statistical Analysis

Sample size

A previous study divided 46 patients with a hoarding disorder (mean age: 53.9 years) into two groups. One group of 23 subjects was scheduled for 26 sessions of cognitive behavioral therapy approximately on a weekly basis. The second group of 23 subjects had yet to undergo cognitive behavioral therapy. [17] A comparison of the two groups indicated that subjects who had completed the 12th session of cognitive behavioral therapy had a 10-point drop in their SI-R score. Around 10 points were improved upon using a pre-test on the general population. A meta-analysis in another study indicated that intervention was more effective in younger individuals than older ones. This study therefore assumed a potential 10-point improvement in SI-R score.[17] Previous results indicated that the SI-R score had a normal distribution and SD was 13.0.[18] The sample size was therefore calculated as 28 per group under the assumptions of a significance level of 5% (two-tailed), a power of 80%, and an effect size of 0.75. Considering the likely dropout rate, we set the sample size needed for this trial as 60 subjects in total, 30 in each group.

Statistical Analysis

Analyses will be conduct under the intention-to-treat (ITT) principle. The main target set for analysis is the full analysis set (FAS). Missing data will be permutated under the assumption of missing at random (MAR) following the last observation carried forward (LOCF) principle. A sensitivity analysis will be performed using the multiple imputation (MI) method. The secondary target set for the analyses will be the per protocol set (PPS).

Summary statistics (maximum, median, minimum, mean, and standard deviation) will be calculated for all continuous data, and frequency and proportion for categorical data.

The differences in effect sizes between the two groups will be examined using a t-test. Differences will be assessed in a general linear model by adjusting for gender, age and marital status at the start of the study, and outcome measure at the baseline. Crude and adjusted odds ratios will be calculated for binary variables and the two groups will be compared using logistic regression analysis. As secondary analysis, mixed-effects random-effects models will be used as a longitudinal data analysis. The significance level for testing will be set at $p < 0.05$ (two-tailed).

Timeframe of the study

Participants were registered between January and May 2016. Eight workshops have been run between 3 February and 11 June 2016. Data are currently being collected.

Data management

Personal information obtained in this study will be coded. Participant files will be stored in numerical order in a secure but accessible place and manner. Data will be anonymized before analysis. Data will be kept on a password-protected computer with limited access. The study organizer will keep all documents related to this trial in a locked cabinet. Five years after the study is presented, or three years after the final publication of the results (whichever is later), all documents will be disposed of using a document destruction service contracted by the University.

Conditions for discontinuation of this study and actions in that event

When significant information about the safety or effectiveness of the intervention is obtained, the study organizer will determine whether to continue the study.

Treatment of subjects after this study is conducted

If the intervention group is performing significantly better than the control group at the end of the study, the control group will be informed. Members of the control group who

For peer review only - http://bmjopen.bmj.com/site/about/guidelines.xhtml

wish to do so may then attend the workshop after the conclusion of this study.

Monitoring

Information on when the study begins, the conduct of the study (sample size), ethical considerations that have been made, the occurrence of any detrimental or adverse events, the results of the study, and registration of the study with a public database will be submitted to the ethics committee in an annual interim report. A report will also be submitted to the ethics committee upon the conclusion of the study, and when the final results are presented.

Protocol amendments

If any occur, the Ethics Committee may be notified as necessary.

Follow-up of adverse events

This study involves attending a workshop program to help people declutter and organize, and receiving visits from a professional organizing and cleaning service. There is little likelihood of any health hazards to the subjects. If any serious adverse events occur, they will be reported in line with the standard operating procedure on Reporting Serious Adverse Events in Clinical Research.

Ethics and dissemination

The Medical Ethical Committee of Teikyo University (No. 15- 065) has approved the study protocol. The findings will be disseminated widely through peer-reviewed publication and conference presentations.

Study participant candidates will be given an explanation form with the items listed below. The study will be fully explained orally and in writing, and voluntary consent for study participation will be sought in writing. Considerations have been made in the event a participant consents to take part but later withdraws that consent. Participants will be assured they will not be penalized for withdrawal or for not participating. Participants will be informed of the study's results after its conclusion and upon their request. A separate informed consent form for minors is applicable for subjects under age 15. Minors living with a guardian or family member (in this section hereinafter "relevant party") will have a similar form mailed to the relevant party or the minor will be asked to deliver it to the relevant party. If information is obtained that may affect the participant or the relevant party, or if the protocol is modified to such an extent that the change would affect the consent of all relevant parties, that information will be promptly provided to the relevant parties and the participant's desire to continue will be verified. In either of the aforementioned instances, the Research Ethics Committee will revise and approve the consent and explanation form and the consent of all relevant parties will be sought.

Participants who wish to view their information or withdraw consent may contact the Study Chair for resolution. Any modifications to the protocol that may affect the conduct of the study will be presented to the aforementioned Committee.

Conflicts of interest

Based on a random number table, home organizing and cleaning services will be randomly assigned to clean the subjects' living space (the organizers will be blind to whether they are in the control or intervention group), to maintain the fairness of this study. Effectiveness of the intervention will be determined using a survey completed by the subject and sent directly to the Study Office, together with a photograph to assist determination of room cleanliness or clutter. Home organizing and cleaning services will not be able to see any results for individual subjects.

For peer review only - http://bmjopen.bmj.com/site/about/guidelines.xhtml

DISCUSSION

One aim of this study is to assess the effect of an intervention (a workshop) on the state of living spaces and the quality of life of younger people who have difficulty decluttering and organizing and who therefore have a cluttered living space. If the approach is found to be effective, then the findings of this study will be widely publicized and the workshop materials will be made widely available, so that it can be conducted by others, including administrative bodies and professional organizers and cleaners. This should improve the quality of life and help to prevent a hoarding disorder in younger people who have difficulty decluttering and organizing.

This study has strengths and weaknesses. Blinding is not possible, because of the type of intervention. It is impossible to create a convincing placebo for the workshop on decluttering and organizing, but this lack of blinding may affect the results. As far as possible, subjects need to have an experience equivalent to undergoing training from a professional organizing and cleaning service.

Acknowledgments
We are grateful for advice from Yukiko Mochizuki, Yuka Nojiri, and Mihoko Shimozono about preparing a study protocol.
The text for the workshop was prepared with help from Keiko Takayama and Mayumi Takahara.

Contributors
YA conceived the study. KY and NA participated in the development of the protocol. MS and YN contributed to study conception and design for the education program. All authors prepared and revised the manuscript, including relevant scientific content. All authors approved the final version of the manuscript.

Funding
This work was supported by JSPS KAKENHI Grant Number JP26671045 (2014–2017) Grant-in-Aid for Scientific Research on "The state of young people who chronically fail to organize or tidy up and development of a program for effective intervention" (Challenging Exploratory Research, Study Organizer: Yasuko Aso, grant no. 26671045). This funding source had no role in the design of this study and will have no role during its execution, analyses, interpretation of the data, or in any decision to submit results.

Competing interests statement
No financial assistance or free services will be received from any company or organization during this study. The researchers have no equity, such as stock, in any of the companies involved, and are not involved in their management, nor have the researchers received any services, free goods or pharmaceuticals, or discounts from those companies.

Subjects' consent
Obtained.

Ethics approval
This trial has been approved by Teikyo University Review Board15-065
Protocol version 1.Issue date 11 November 2015, Authors: *YA, KY,NA.*

Data sharing statement
The informed consent documents are available on request.

For peer review only - http://bmjopen.bmj.com/site/about/guidelines.xhtml

REFERENCES

1. American Psychiatric Association. Diagnostic and statistical manual of mental disorder (DSM). Washington, DC: American Psychiatric Association 1994:143–7.
2. Frost RO, Steketee G, Grisham JR. Measurement of compulsive hoarding: saving inventory-revised. Behav Res Ther 2004;42(10):1163–82.
3. Tolin DF, Frost RO, Steketee G, et al. The economic and social burden of compulsive hoarding. *Psychiatry Res* 2008;160(2):200–11.
4. Kishi E, Nojiri Y, Yonezawa J, et al. An analysis of forms of intervention by nurses at comprehensive community support centers in cases of self-neglect. *Journal of the Japan Academy for the Prevention of Elder Abuse* 2014;10(1):106–20.
5. Frank H, Stewart E, Walther M, et al. A. Hoarding behavior among young children with obsessive–compulsive disorder. *Journal of obsessive-compulsive and related disorders* 2014; 3(1): 6-11.
6. Samuels JF, Bienvenu OJ, Riddle MA, et al. Hoarding in obsessive compulsive disorder: results from a case-control study. Behav Res Ther 2002 May;40(5):517–528.[PubMed]
7. Frost RO, Krause MS, Steketee G. Hoarding and obsessive-compulsive symptoms. Behavior Modification 1996;20:116–132. [PubMed]
8. Rasmussen SA, Eisen JL. Clinical features and phenomenology of obsessive compulsive disorder. *Psychiatric Annals* 1989;19:67–73.
9. Sobin C, Blundell ML, Weiller F, et al. Evidence of a schizotypy subtype in OCD. *Journal of Psychiatric Research* 2000;34:15–24.[PubMed]
10. Saxena S, Maidment KM, Vapnik T, et al. Obsessive-compulsive hoarding: symptom severity and response to multimodal treatment.*J Clin Psychiatry* 2002 Jan;63(1):21–27.[PubMed]
11. Timpano K R, Raines A M, Shaw A M, et al. Effects of a brief anxiety sensitivity reduction intervention on obsessive compulsive spectrum symptoms in a young adult sample.*Journal of Psychiatric Research* 2016;83, 8-15.
12. Grisham JR, Frost RO, Steketee G, et al. Age of onset of compulsive hoarding. *J Anxiety Disord* 2006;20(5):675–86.
13. Tolin, D. F., Meunier, S. A., Frost, R. O., Steketee, G. Course of compulsive hoarding and its relationship to life events. *Depression and Anxiety*, 2010;27(9):29-838.
14. Nordsletten AE. Epidemiology of hoarding disorder. *Br J Psychiatry* 2013;203:445–52.
15. Tolin DF, Frost RO, Steketee G. Cognitive behavioral therapy for hoarding disorder: A meta-analysis. *Depress Anxiety* 2015;32(3):158–66.
16. Steketee G, Frost R O, Tolin D F, et al. Waitlist - controlled trial of cognitive behavior therapy for hoarding disorder. *Depression and anxiety* 2010;27(5):476-484.
17. Frost R O, Ruby D, Shuer L J. The buried in treasures workshop: waitlist control trial of facilitated support groups for hoarding. *Behaviour research and therapy* 2012; 50(11), 661-667.
18. Aso Y, Masashige S, Yuka N, et al. Attitudes of undergraduates studying nursing or welfare work towards organizing and tidying up and the state of their living spaces. [abstract]. Nagasaki; Japan, The 74th Annual Meeting of the Japanese Society of Public Health 2015;p294.
19. Aki T, Ken-ichi K, Toko I, et al. A consideration of clinical characteristics of non-clinical hoarding among Japanese adolescents. *Japanese Society of Anxiety and Related Disorder* 2015;6(2):72–85.
20. Kazue Y, Toshiro T. "Effects of lifestyle modification on metabolic syndrome: a systematic review and meta-analysis. *BMC medicine* 2012; 1

For peer review only - http://bmjopen.bmj.com/site/about/guidelines.xhtml

21. Anderson L M, Quinn T A, Glanz K, et al. The effectiveness of worksite nutrition and physical activity interventions for controlling employee overweight and obesity: a systematic review. *American journal of preventive medicine* 2009;37(4):340-357.
22. Fun Cleansing for Elementary School Children - Life Organization to Start at the age of 10:Japan Association of Life Organizers:2015.
23. Storch E A, Muroff J, Lewin A B, et al. Development and preliminary psychometric evaluation of the Children's Saving Inventory.Child Psychiatry & Human Development 2011;42(2): 166-182.
24. http://www.compareyourcountry.org/pisa/country/oecd (accessed 20 Feb 2017).
25. Frost RO, Steketee G. Compulsive Hoarding and Acquiring: Therapist Guide. Japanese edition. Toko I, translator. Tokyo: Kanekoshobou 2013:170–3.
26. Frost RO, Steketee G, Tolin DF, et al. Development and validation of the Clutter Image Rating. *J Psychopathol Behav Assess* 2008;30(3):193–203.
27. MarikoY, Yutaka M, Yukiko Y. The Structure of Perceived Aspects of Self. *Japanese Association of Educational Psychology* 1982;30(1):64–8.
28. Tomari S, Yoshida F. Psychological review of meanings of private space and its privacy research and proposal for functions: new models. *Tsukuba Psychological Research* 1998;20:173–90.

FIGURE TITLES

Figure 1. Trial design

For peer review only

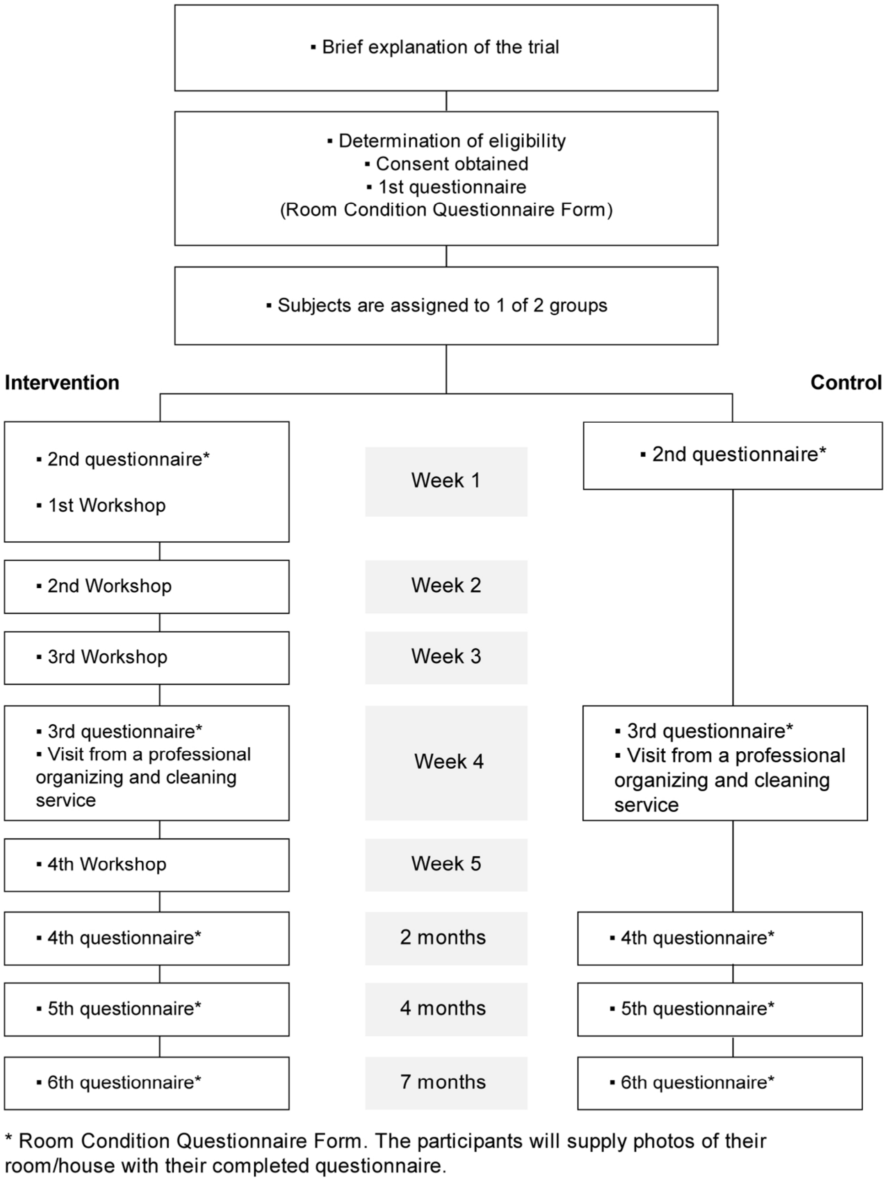

Figure 1. Trial design

140x177mm (300 x 300 DPI)

STANDARD PROTOCOL ITEMS: RECOMMENDATIONS FOR INTERVENTIONAL TRIALS

SPIRIT 2013 Checklist: Recommended items to address in a clinical trial protocol and related documents*

Section/item	Item No	Description	Addressed on page number
**Administrative information**			
Title	1	Descriptive title identifying the study design, population, interventions, and, if applicable, trial acronym	1
Trial registration	2a	Trial identifier and registry name. If not yet registered, name of intended registry	2
	2b	All items from the World Health Organization Trial Registration Data Set	All items were covered.
Protocol version	3	Date and version identifier	10
Funding	4	Sources and types of financial, material, and other support	10
Roles and responsibilities	5a	Names, affiliations, and roles of protocol contributors	10, 1
	5b	Name and contact information for the trial sponsor	not applicable
	5c	Role of study sponsor and funders, if any, in study design; collection, management, analysis, and interpretation of data; writing of the report; and the decision to submit the report for publication, including whether they will have ultimate authority over any of these activities	10
	5d	Composition, roles, and responsibilities of the coordinating centre, steering committee, endpoint adjudication committee, data management team, and other individuals or groups overseeing the trial, if applicable (see Item 21a for data monitoring committee)	Study Protocol(p9-10)

For peer review only - http://bmjopen.bmj.com/site/about/guidelines.xhtml

Introduction

Background and rationale	6a	Description of research question and justification for undertaking the trial, including summary of relevant studies (published and unpublished) examining benefits and harms for each intervention	3 -4
	6b	Explanation for choice of comparators	6
Objectives	7	Specific objectives or hypotheses	4
Trial design	8	Description of trial design including type of trial (eg, parallel group, crossover, factorial, single group), allocation ratio, and framework (eg, superiority, equivalence, noninferiority, exploratory)	4

Methods: Participants, interventions, and outcomes

Study setting	9	Description of study settings (eg, community clinic, academic hospital) and list of countries where data will be collected. Reference to where list of study sites can be obtained	1,5
Eligibility criteria	10	Inclusion and exclusion criteria for participants. If applicable, eligibility criteria for study centres and individuals who will perform the interventions (eg, surgeons, psychotherapists)	4, Table 1
Interventions	11a	Interventions for each group with sufficient detail to allow replication, including how and when they will be administered	6,Table 2
	11b	Criteria for discontinuing or modifying allocated interventions for a given trial participant (eg, drug dose change in response to harms, participant request, or improving/worsening disease)	8-9
	11c	Strategies to improve adherence to intervention protocols, and any procedures for monitoring adherence (eg, drug tablet return, laboratory tests)	6
	11d	Relevant concomitant care and interventions that are permitted or prohibited during the trial	6
Outcomes	12	Primary, secondary, and other outcomes, including the specific measurement variable (eg, systolic blood pressure), analysis metric (eg, change from baseline, final value, time to event), method of aggregation (eg, median, proportion), and time point for each outcome. Explanation of the clinical relevance of chosen efficacy and harm outcomes is strongly recommended	7
Participant timeline	13	Time schedule of enrolment, interventions (including any run-ins and washouts), assessments, and visits for participants. A schematic diagram is highly recommended (see Figure)	Figure 1,

For peer review only - http://bmjopen.bmj.com/site/about/guidelines.xhtml

Sample size	14	Estimated number of participants needed to achieve study objectives and how it was determined, including clinical and statistical assumptions supporting any sample size calculations	8
Recruitment	15	Strategies for achieving adequate participant enrolment to reach target sample size	5
**Methods: Assignment of interventions (for controlled trials)**			
Allocation:			
Sequence generation	16a	Method of generating the allocation sequence (eg, computer-generated random numbers), and list of any factors for stratification. To reduce predictability of a random sequence, details of any planned restriction (eg, blocking) should be provided in a separate document that is unavailable to those who enrol participants or assign interventions	5
Allocation concealment mechanism	16b	Mechanism of implementing the allocation sequence (eg, central telephone; sequentially numbered, opaque, sealed envelopes), describing any steps to conceal the sequence until interventions are assigned	5
Implementation	16c	Who will generate the allocation sequence, who will enrol participants, and who will assign participants to interventions	5
Blinding (masking)	17a	Who will be blinded after assignment to interventions (eg, trial participants, care providers, outcome assessors, data analysts), and how	5-6
	17b	If blinded, circumstances under which unblinding is permissible, and procedure for revealing a participant's allocated intervention during the trial	Not applicable
**Methods: Data collection, management, and analysis**			
Data collection methods	18a	Plans for assessment and collection of outcome, baseline, and other trial data, including any related processes to promote data quality (eg, duplicate measurements, training of assessors) and a description of study instruments (eg, questionnaires, laboratory tests) along with their reliability and validity, if known. Reference to where data collection forms can be found, if not in the protocol	3

For peer review only - http://bmjopen.bmj.com/site/about/guidelines.xhtml

	18b	Plans to promote participant retention and complete follow-up, including list of any outcome data to be collected for participants who discontinue or deviate from intervention protocols	6
Data management	19	Plans for data entry, coding, security, and storage, including any related processes to promote data quality (eg, double data entry; range checks for data values). Reference to where details of data management procedures can be found, if not in the protocol	8
Statistical methods	20a	Statistical methods for analysing primary and secondary outcomes. Reference to where other details of the statistical analysis plan can be found, if not in the protocol	8
	20b	Methods for any additional analyses (eg, subgroup and adjusted analyses)	8
	20c	Definition of analysis population relating to protocol non-adherence (eg, as randomised analysis), and any statistical methods to handle missing data (eg, multiple imputation)	8
**Methods: Monitoring**			
Data monitoring	21a	Composition of data monitoring committee (DMC); summary of its role and reporting structure; statement of whether it is independent from the sponsor and competing interests; and reference to where further details about its charter can be found, if not in the protocol. Alternatively, an explanation of why a DMC is not needed	Not applicable
	21b	Description of any interim analyses and stopping guidelines, including who will have access to these interim results and make the final decision to terminate the trial	Not applicable
Harms	22	Plans for collecting, assessing, reporting, and managing solicited and spontaneously reported adverse events and other unintended effects of trial interventions or trial conduct	9-10
Auditing	23	Frequency and procedures for auditing trial conduct, if any, and whether the process will be independent from investigators and the sponsor	9
**Ethics and dissemination**			
Research ethics approval	24	Plans for seeking research ethics committee/institutional review board (REC/IRB) approval	10

For peer review only - http://bmjopen.bmj.com/site/about/guidelines.xhtml

Protocol amendments	25	Plans for communicating important protocol modifications (eg, changes to eligibility criteria, outcomes, analyses) to relevant parties (eg, investigators, REC/IRBs, trial participants, trial registries, journals, regulators)	9
Consent or assent	26a	Who will obtain informed consent or assent from potential trial participants or authorised surrogates, and how (see Item 32)	9
	26b	Additional consent provisions for collection and use of participant data and biological specimens in ancillary studies, if applicable	Not applicable
Confidentiality	27	How personal information about potential and enrolled participants will be collected, shared, and maintained in order to protect confidentiality before, during, and after the trial	8
Declaration of interests	28	Financial and other competing interests for principal investigators for the overall trial and each study site	10
Access to data	29	Statement of who will have access to the final trial dataset, and disclosure of contractual agreements that limit such access for investigators	10
Ancillary and post-trial care	30	Provisions, if any, for ancillary and post-trial care, and for compensation to those who suffer harm from trial participation	8-9
Dissemination policy	31a	Plans for investigators and sponsor to communicate trial results to participants, healthcare professionals, the public, and other relevant groups (eg, via publication, reporting in results databases, or other data sharing arrangements), including any publication restrictions	9
	31b	Authorship eligibility guidelines and any intended use of professional writers	10
	31c	Plans, if any, for granting public access to the full protocol, participant-level dataset, and statistical code	10
**Appendices**			
Informed consent materials	32	Model consent form and other related documentation given to participants and authorised surrogates	9
Biological specimens	33	Plans for collection, laboratory evaluation, and storage of biological specimens for genetic or molecular analysis in the current trial and for future use in ancillary studies, if applicable	Not Applicable

For peer review only - http://bmjopen.bmj.com/site/about/guidelines.xhtml

*It is strongly recommended that this checklist be read in conjunction with the SPIRIT 2013 Explanation & Elaboration for important clarification on the items. Amendments to the protocol should be tracked and dated. The SPIRIT checklist is copyrighted by the SPIRIT Group under the Creative Commons "Attribution-NonCommercial-NoDerivs 3.0 Unported" license.

For peer review only

For peer review only - http://bmjopen.bmj.com/site/about/guidelines.xhtml

BMJ Open

Effectiveness of a "Workshop on Decluttering and Organizing" program for teens and middle-aged adults with difficulty decluttering: a study protocol of an open-label, randomized, parallel-group, superiority trial in Japan

Journal:	*BMJ Open*
Manuscript ID	bmjopen-2016-014687.R3
Article Type:	Protocol
Date Submitted by the Author:	07-Apr-2017
Complete List of Authors:	Aso, Yasuko; Teikyo University, Department of Nursing, Faculty of Medical Technology<br>Yamaoka, Kazue; Teikyo University, Graduate School of Public Health<br>Nemoto, Asuka; Teikyo University, Graduate School of Public Health<br>Naganuma, Yuki; Teikyo University, Department of Nursing, Faculty of Medical Technology<br>Saito, Masashige; Nihon Fukushi University, Department of Social Welfare
**Primary Subject Heading**:	Public health
Secondary Subject Heading:	Nursing, Mental health, Health services research
Keywords:	Saving Inventory–Revised, Workshop on Decluttering and Organizing, Study protocol, Randomized controlled trial, Teens and midd, Protocols & guidelines < HEALTH SERVICES ADMINISTRATION & MANAGEMENT, PUBLIC HEALTH

SCHOLARONE™
Manuscripts

Effectiveness of a "Workshop on Decluttering and Organizing" program for teens and middle-aged adults with difficulty decluttering: a study protocol of an open-label, randomized, parallel-group, superiority trial in Japan

Yasuko Aso[1,2], Kazue Yamaoka[2], Asuka Nemoto[2], Yuki Naganuma[3], Masashige Saito[4]

Author Affiliations
[1] Department of Nursing, Faculty of Health Sciences,Tsukuba International University, Tuchiura ,Ibaraki, Japan
[2] Teikyo University Graduate School of Public Health, Tokyo, Japan
[3] Department of Nursing, Faculty of Nursing, Tokyo Ariake University of Medical and Health Sciences, Tokyo, Japan
[4] Department of Social Welfare, Faculty of Social Welfare, Nihon Fukushi University, Aichi, Japan

Correspondence to
Yasuko Aso.
Postal address: 6-8-33 Manabe, Tuchiura-shi, Ibaraki 300-0051
Email: y-asou@tius.ac.jp
Tel: +81-29-826- 6622(6003)

Key words: Saving Inventory–Revised, Workshop on Decluttering and Organizing, Study protocol, Randomized controlled trial, Teens and middle-aged adults, Effectiveness

Words 3753

For peer review only

For peer review only - http://bmjopen.bmj.com/site/about/guidelines.xhtml

Abstract

Introduction: Hoarding disorder can cause problems with work performance, personal hygiene, health, and well-being. The disorder is a growing social problem in Japan. Having difficulty discarding rubbish, decluttering, and organizing can signal a future hoarding disorder, and early intervention is important. We developed an educational workshop on decluttering and organizing for teens and adults with difficulty organizing. The objective of this study is to evaluate the effectiveness of a workshop for reducing clutter and improving quality of life among younger people with difficulty decluttering and organizing.

Methods and analysis: An open-label, parallel-group randomized controlled trial will be conducted among volunteers aged 12–55 years with mild difficulty decluttering and organizing. Those in the intervention group will attend the workshop and receive a visit from a professional cleaning company to declutter their living space. The control group will have only the latter. The primary outcome will be the score on the Japanese version of the Saving Inventory–Revised. Secondary outcomes will be scores on the Clutter Image Rating Scale, the Japanese version of the Rosenberg Self-Esteem Scale, and the Roles of Private Space Scale. The results will be examined for differences between the two groups in changes from baseline to 7 months. We will examine crude effects and adjust for gender and age using a general linear model for continuous variables and a logistic regression model for dichotomous variables. Sample size was calculated assuming a significance level of 5% (two-tailed), a power of 80%, and an effect size of 0.75. In total, 60 subjects (30 in each group) will be required.

Ethics and dissemination: The study protocol has been approved by the Medical Ethical Committee of Teikyo University (No. 15-065-2). The findings will be disseminated widely through peer-reviewed publication and conference presentations.

Trial registration number: UMIN000020568. Issue Date: 16 January 2016

Strengths and limitations of this study

- The study will be an open-label, parallel-group randomized controlled trial and, to our knowledge, is the first such study on this subject in Japan.
- The study will test a simple but feasible intervention for those with difficulty decluttering and organizing.
- The outcomes measures are clearly set using reliable scales.
- The test is adequately powered to detect effects on the outcomes.
- Blinding is not possible because of the type of intervention, which may affect the results.

For peer review only

For peer review only - http://bmjopen.bmj.com/site/about/guidelines.xhtml

INTRODUCTION

Having a hoarding disorder can cause problems with work performance, personal hygiene, health, and well-being. Such disorders occasionally result in individuals living in environments with large amounts of garbage. Key symptoms of hoarding include difficulty discarding, excessive clutter, and excessive acquisition of objects.[1,2] The latter symptoms pose a number of dangers, such as the potential for fire and tripping over clutter. The presence of trash or clutter in one's living space also affects an individual's overall quality of life.[3] This issue is a growing social problem in Japan.[4]

An individual living in such conditions can become isolated from the rest of society; this can be particularly true for older people.[5] Unsanitary living conditions can eventually become a public health problem if they affect one's neighbors. Hoarding disorder is therefore not only an individual problem but also a problem for society, and a practical solution is warranted from a public health point of view.

Hoarding disorder is often accompanied by psychiatric disorders such as attention deficit hyperactivity disorder (ADHD), depression, or obsessive–compulsive disorder (OCD).[6-11] A relationship between hoarding disorder and distress tolerance and anxiety sensitivity has also been noted in a sample of young adults.[12] Signs of hoarding were separately reported in children aged 13[13] and 11–15,[14] and the severity of the disorder evidently increases with age. Many sufferers report symptom onset before age 20,[14] with symptoms tending to worsen past age 55.[13] This suggests that prevention among younger people is important. To accommodate this, we targeted a wide range of participants aged 12–55 years old.

A previous study in England and Germany estimated that incidence of hoarding disorder was 1.5%–5.8%.[15,16] Another study reported that cognitive behavioral therapy, either individually or in groups, as well as home visits, are effective at assisting patients with hoarding disorder. However, these cognitive behavioral therapies required relatively long intervention periods and the subject samples were middle-aged and elderly people.[17-19]

To the contrary, a study on the effects of a brief anxiety sensitivity reduction intervention on obsessive–compulsive spectrum symptoms in a young adult sample showed reduced OC symptoms across the post-intervention follow-up period. However, the intervention did not show a specific effect on reducing hoarding symptoms.[12] There is a clear need to identify new avenues through which to develop additional interventions.

Japan's population is aging at an increasingly rapid rate. Given the association with age, early intervention is imperative. There is urgent need to establish a system to help prevent younger people who show signs of difficulty decluttering and organizing from developing a hoarding disorder.

A previous study in Japan examined the state of the living space of 450 undergraduates from two universities.[20] The results showed that younger people who had difficulty decluttering and organizing tended to live in cluttered or disorganized living spaces and to have low self-esteem. The study used the Japanese version of the Saving Inventory–Revised (SI-R).[21] This was developed in the United States[2] and translated into Japanese, and its reliability and validity have been verified.[2] The previous study's SI-R scores had a mean of 32.1 points (±13.0) and a normal distribution. SI-R scores have been reported to be high in people who said that they did not like to clean, but many such people also want to improve the state of their living space.[20] There are various ways to learn how to clean effectively in Japan, including workshops, books, and professional support, but it is not clear which method is most effective.

We have therefore developed an education program, of a series of workshops on decluttering and organizing, for teens and adults who find it difficult to organize. In the workshops, we will help identify what belongings a person truly needs. It also covers the state of the individual's living space and its relationship with physical and mental health.

For peer review only - http://bmjopen.bmj.com/site/about/guidelines.xhtml

The purpose of the workshop is to improve recognition of the importance of cleaning and to change behavior.

This study examines behavior 7 months after the intervention, using a control group, and is designed to assess both changes in habits and levels of stress. It is impossible to blind this type of intervention, so the study was made open-label to minimize bias.

The aim of the study is to evaluate the effectiveness of the program in reducing clutter and improving the quality of life in teens and middle-aged adults who have difficulty decluttering and organizing and have a cluttered living space.

METHODS AND ANALYSIS

Study design

The design of this study is an open-label, parallel-group, stratified, randomized controlled trial (RCT). The aim of this trial is to investigate the effect of the workshop plus a professional organizing and cleaning service. We hope that the program will improve quality of life and help prevent hoarding disorder in younger people who currently have difficulty decluttering and organizing. Participants will be involved for a period of 7 months. The design of the trial is shown in Figure 1. Based on previous experience, it is expected that the study will include around 90% of eligible participants. Perioperative protocols are standardized.

[Insert Figure 1 (Trial design) about here]

Participants

The study will look for volunteers aged between 12 and 55, and who have mild organization difficulties. Volunteers will need to meet all inclusion criteria below and not meet any of the exclusion criteria (see Table 1).

Table 1 Inclusion criteria and exclusion criteria

Inclusion criteria

- Individuals who have difficulty decluttering and organizing. (SI-R score≥30)
- Individuals with a total score of four points or more on the Japanese version of the Clutter Image Rating (CIR) scale Note 1.
- Individuals who are responsible for decluttering and organizing their own rooms.
- Individuals with no planned relocations, extensive renovations of living space, or room changes (to allow comparison of the state of living spaces before and after the intervention).
- Individuals who are able to attend at least three workshop sessions and receive visits from an organizing and cleaning service.
- Individuals who responded in the questionnaire, "I want to organize my room according to my will".
- Individuals who can send photographs of their living spaces at the specified time, i.e. without delay.
- Individuals or a roommate/family member (the informed consent form for minors if the individual is under the age of 15) who consent in writing to their participation in the study, and to having a better organized and tidied living space. If the individual is a minor, consent must be provided by a guardian.
- Individuals between the ages of 12 and 55 at the start of the study.

Exclusion criteria

- Individuals who are unable to organize and tidy up their living spaces during the study period, for example:
  - ✓ Individuals with an illness or disability
  - ✓ Individuals who are not living at the registered residence or are physically unable to regularly organize and tidy up

For peer review only - http://bmjopen.bmj.com/site/about/guidelines.xhtml

- Individuals living in a space that is too messy (An organization expert and two cleaning experts judge the space and deem whether it can be sufficiently organized within about 3 hours.)
- Individuals who harass other attendees while attending the workshop or who are deemed incapable of obeying the specified rules of attendance.
- Individuals who are rendered incapable of participation during this study.
- Individuals suffering from a condition or disability that is deemed to preclude study participation. If there are difficulties with that determination, an individual may be barred from participation at the discretion of a physician.
- Individuals who are otherwise deemed ineligible by an investigator (or sub-investigator).
-

Note 1 The CIR scale score for the general public is a mean of 3.8 points (±1)[20] so the subjects in this study must have at least that score.

Recruitment and allocation

Subject recruitment

We will recruit participants in several ways, including through posters put up in visiting nursing stations, visiting care stations, community pharmacies, and at a university, high school, and junior high school in northwest Tokyo. We will also recruit online using a homepage, blog, and social media for an events corporation, an association of organizers, and a non-profit organization that provides support for people with ADHD.

Subject assignment

Applicants will visit Teikyo University to receive an explanation of the study objectives. After assessment via questionnaire, those verified as eligible will be immediately enrolled if they give their full consent. Each subject will then be randomly assigned to either the intervention or control group. Enrollment will continue until the required sample size (30 participants per group) is reached. We consider that the effects of lifestyle education need several months to show progress; many studies use 6 months or longer.[22,23] We therefore set a term of 7 months by considering 6 months after the intervention begins as the observation period in the present study.

Randomization and blinding

A sealed envelope method will be used for random allocation. This is based on a permuted block method, with a block size of four and a random number table created before the study begins. Each pre-sealed envelope, prepared by a person independent from the research, will contain a random number. Participants will open this at the location of the session meeting where they gave consent, and cannot change their designated group. Because of the open-label design, participants will know whether they are assigned to the intervention or control group. However, this information is blinded for the professional organizing and cleaning service in order to avoid risk of bias.

Effectiveness of the intervention will be determined via surveys completed by the participants, and sent directly to the study chair at Teikyo-Univercity along with a photograph of the room to assess the present level of cleanliness or clutter. The organizing and cleaning services will not be able to see the results for individual subjects.

Interventions

Intervention group

This group will attend a workshop program, undergo inspections of cleanliness or clutter of their rooms, and receive visits from an organizing and cleaning service. The workshop will consist of four sessions. Room cleanliness or clutter will be inspected six times. The first inspection will take place once eligibility is verified and consent is obtained. The

For peer review only

second will take place before the start of the study. Others will take place 4 weeks, 2 months, 4 months, and 7 months after the start of the study (see Figure 1).

Control group

The control group will receive an initial inspection to evaluate room cleanliness or clutter once eligibility is verified and consent obtained. The second inspection will take place before the intervention group starts the workshop. The control group will receive a visit from a professional cleaning company only to declutter the living spaces. Further inspections will take place 4 weeks, 2 months, 4 months, and 7 months after the start of the study. Members of the control group who wish to attend the workshop may do so after the study period ends.

Additionally, until the end of the research period 7 months after commencement of the study, no participants are allowed to directly request any cleaning service be done.

The content of the workshop

The workshop's purpose is to improve participant behavior and develop habits of decluttering and organizing. The workshop starts by covering the relationship between physical and mental health and the state of the living space and then discusses the participants' ideal life. A study representative has developed a workshop program based on material from the Japan Association of Life Organizers and Edison Club, a nonprofit organization supporting patients with ADHD, their families, and teachers. The textbook used in the workshop is based on a text used in elementary schools;[24] therefore, 12-year-olds should have no difficulty understanding the contents.

A public health nurse and an organizer will deliver four lectures, with the former leading the first 30 minutes of each lecture and the latter the final 1.5 hours (see Table 2 for details).

Table 2 Syllabus for "Workshop on Decluttering and Organizing"

1st<br>(2_hours)	<Reduce><br>·Orientation and goal setting. (public health nurse)<br>·Understanding the importance of working as a group. (public health nurse)<br>·Three fundamentals for healthy and comfortable living (reduce, organize, maintain). (organizer)<br>·Organizing in small steps: around your desk and your bed. (organizer)<br>·Explanation of homework. (public health nurse)
2nd<br>(2_hours)	<Organize><br>·Review of previous session (public health nurse)<br>·The relation between decluttering and organizing and health (fall prevention, allergic disease) (public health nurse)<br>·Organizing in small steps: the kitchen (organizer)<br>·Explanation of homework. (public health nurse)
3rd<br>(2_hours)	< Review ><br>·Review of previous session (public health nurse)<br>·Solutions for people who have difficulty discarding things and buy (or receive) unnecessary things (public health nurse)<br>·Organizing in small steps: a place to relax(organizer)<br>·Explanation of homework. (public health nurse)
4th<br>(2_hours)	< Maintain><br>·Review of previous session (public health nurse)<br>·Organizing in small steps: closets and wardrobes (organizer)<br>·Schedule of questionnaire submission (public health nurse)

Outcome measures and subject's characteristics

Primary outcome

The primary outcome is the score on the Japanese version of the Saving

For peer review only

For peer review only - http://bmjopen.bmj.com/site/about/guidelines.xhtml

Inventory-Revised (SI-R). The SI-R score is an index used to gauge the difficulties posed by a hoarding disorder (a higher score indicates a greater tendency to hoard). It measures mental anguish caused by hoarding, and the impacts of hoarding on life. A higher score indicates a greater tendency to hoard.[2,21] There is presently no Japanese version of the Child Saving Inventory.[25] The academic ability of Japanese teenagers, however, ranks among the top in the world.[26] Only 292 Japanese *kanji* characters are used for the Japanese version of the SI-R, and most have already been learned in elementary school. Therefore, the Japanese version of the SI-R is applicable for junior high school students and should not create problems related to comprehension.

The primary effect size is the difference changes in SI-R score from baseline to 7 months between the two groups. The hypothesis of this study is that the mean SI-R score of the intervention group will decrease more than that of the control group.

Secondary outcomes

Secondary outcomes include:

- Score on the Clutter Image Rating (CIR) scale, which measures clutter using photographs of rooms (a higher score indicates more clutter).[27,28]
- Score on the Japanese version of the Rosenberg Self-Esteem Scale (RSES).[29] This measures the degree of self-respect and the value placed on the self. A high score shows stronger feelings of self-esteem.
- Score on the Roles of Private Space Scale (RPSS). This linear measure observes the function, the necessity and securement level of private space.[30]

These outcomes will be used to examine the amount of decluttering and organizing, including frequency of cleaning and number of visitors in a week. They will allow the researchers to assess the state of subjects' living spaces and their ability to declutter and organize because of the workshop.

Subject characteristics

During the first meeting, participants will provide information about their gender, age, co-morbidities, previous history, medication, number of roommates/family members in their household, number and size of living spaces, how often their living space is organized and tidied up, number of visitors to their living space in the previous week, whether they have difficulty decluttering and organizing, and are living with a roommate or family members, and aspects of social status such as occupation, marital status, educational background, and work pattern (whether they are working on a shift basis).

Statistical Analysis

Sample size

A previous study divided 46 patients with a hoarding disorder (mean age: 53.9 years) into two groups. One group of 23 subjects was scheduled for 26 sessions of cognitive behavioral therapy approximately on a weekly basis. The second group of 23 subjects had yet to undergo cognitive behavioral therapy.[19] A comparison of the two groups indicated that subjects who had completed the 12th session of cognitive behavioral therapy had a 10-point drop in their SI-R score. Around 10 points were improved upon using a pre-test on the general population. A meta-analysis in another study indicated that intervention was more effective in younger individuals than older ones. This study therefore assumed a potential 10-point improvement in SI-R score.[19] Previous results indicated that the SI-R score had a normal distribution and SD was 13.0.[20] The sample size was therefore calculated as 28 per group under the assumptions of a significance level of 5% (two-tailed), a power of 80%, and an effect size of 0.75. Considering the likely dropout rate, we set the sample size needed for this trial as 60 subjects in total, 30 in each group.

Statistical Analysis

For peer review only - http://bmjopen.bmj.com/site/about/guidelines.xhtml

Analyses will be conducted under the intention-to-treat (ITT) principle. The main target set for analysis is the full analysis set (FAS). Missing data will be permutated under the assumption of missing at random (MAR) following the last observation carried forward (LOCF) principle. A sensitivity analysis will be performed using the multiple imputation (MI) method. The secondary target set for the analyses will be the per protocol set (PPS).

Summary statistics (maximum, median, minimum, mean, and standard deviation) will be calculated for all continuous data, and frequency and proportion for categorical data.

The differences in effect sizes between the two groups will be examined using a t-test. Differences will be assessed in a general linear model by adjusting for gender, age and marital status at the start of the study, and outcome measure at the baseline. Crude and adjusted odds ratios will be calculated for binary variables and the two groups will be compared using logistic regression analysis. As secondary analysis, mixed-effects random-effects models will be used as a longitudinal data analysis. The significance level for testing will be set at $p < 0.05$ (two-tailed).

Timeframe of the study

Participants were registered between January and May 2016. Eight workshops have been run between 3 February and 11 June 2016. Data are currently being collected.

Data management

Personal information obtained in this study will be coded. Participant files will be stored in numerical order in a secure but accessible place and manner. Data will be anonymized before analysis. Data will be kept on a password-protected computer with limited access. The study organizer will keep all documents related to this trial in a locked cabinet. Five years after the study is presented, or three years after the final publication of the results (whichever is later), all documents will be disposed of using a document destruction service contracted by the University.

Conditions for discontinuation of this study and actions in that event

When significant information about the safety or effectiveness of the intervention is obtained, the study organizer will determine whether to continue the study.

Treatment of subjects after this study is conducted

If the intervention group is performing significantly better than the control group at the end of the study, the control group will be informed. Members of the control group who wish to do so may then attend the workshop after the conclusion of this study.

Monitoring

Information on when the study begins, the conduct of the study (sample size), ethical considerations that have been made, the occurrence of any detrimental or adverse events, the results of the study, and registration of the study with a public database will be submitted to the ethics committee in an annual interim report. A report will also be submitted to the ethics committee upon the conclusion of the study, and when the final results are presented.

Protocol amendments

If any occur, the Ethics Committee may be notified as necessary.

Follow-up of adverse events

This study involves attending a workshop program to help people declutter and organize, and receiving visits from a professional organizing and cleaning service. There is little likelihood of any health hazards to the subjects. If any serious adverse events occur, they will be reported in line with the standard operating procedure on Reporting Serious

For peer review only - http://bmjopen.bmj.com/site/about/guidelines.xhtml

Adverse Events in Clinical Research.

Ethics and dissemination

The Medical Ethical Committee of Teikyo University (No. 15- 065-2) has approved the study protocol. The findings will be disseminated widely through peer-reviewed publication and conference presentations.

Study participant candidates will be given an explanation form. The study will be fully explained orally and in writing, and voluntary consent for study participation will be sought in writing. The explanation and consent form is shown in Supplementary files 1-8. Considerations have been made in the event a participant consents to take part but later withdraws that consent. Participants will be assured they will not be penalized for withdrawal or for not participating. Participants will be informed of the study's results after its conclusion and upon their request. Those aged 12–14 must provide written informed assent together with an adult consent form from the parents. Those aged 15–18 must provide written consent (same as for adults) and a guardian consent form. The ages for requisite informed assent were decided with reference to the guidelines of the American Academy of Pediatrics[31,32] and the decision of the Teikyo University Ethics Committee. Minors living with a guardian or family member (in this section hereinafter "relevant party") will have a similar form mailed to the relevant party or the minor will be asked to deliver it to the relevant party. If information is obtained that may affect the participant or the relevant party, or if the protocol is modified to such an extent that the change would affect the consent of all relevant parties, that information will be promptly provided to the relevant parties and the participant's desire to continue will be verified. In either of the aforementioned instances, the Research Ethics Committee will revise and approve the consent and explanation form and the consent of all relevant parties will be sought.

Participants who wish to view their information or withdraw consent may contact the Study Chair for resolution. Any modifications to the protocol that may affect the conduct of the study will be presented to the aforementioned Committee.

[Insert supplementary Files 1
(Explanation Document (The Subjects themselves from 12 years old to 14 years old)Japanese version) about here]

[Insert supplementary Files 2
(Explanation Document (The Subjects themselves from 12 years old to 14 years old) about here]

[Insert supplementary Files 3
(Explanation Document (The subjects themselves of 15 years old or older_Parents_Those who live together) Japanese version) about here]

[Insert supplementary Files 4
(Explanation Document (The subjects themselves of 15 years old or older_Parents_Those who live together) about here]

[Insert supplementary Files 5
(Consent Form (Subject themselves from 12 years old to 14 years old) Japanese version) about here]

[Insert supplementary Files 6
(Consent Form (Subject themselves from 12 years old to 14 years old) about here]

For peer review only - http://bmjopen.bmj.com/site/about/guidelines.xhtml

[Insert supplementary Files 7
(Consent Form (Subjects themselves from 15 years old or older and parents and those who live together Japanese version) about here]

[Insert supplementary Files 8
(Consent Form (Subjects themselves from 15 years old or older and parents and those who live together) about here]

Conflicts of interest

Based on a random number table, home organizing and cleaning services will be randomly assigned to clean the subjects' living space (the organizers will be blind to whether they are in the control or intervention group), to maintain the fairness of this study. Effectiveness of the intervention will be determined using a survey completed by the subject and sent directly to the Study Office, together with a photograph to assist determination of room cleanliness or clutter. Home organizing and cleaning services will not be able to see any results for individual subjects.

DISCUSSION

One aim of this study is to assess the effect of an intervention (a workshop) on the state of living spaces and the quality of life of younger people who have difficulty decluttering and organizing and who therefore have a cluttered living space. If the approach is found to be effective, then the findings of this study will be widely publicized and the workshop materials will be made widely available, so that it can be conducted by others, including administrative bodies and professional organizers and cleaners. This should improve the quality of life and help to prevent a hoarding disorder in younger people who have difficulty decluttering and organizing.

This study has certain strengths and weaknesses. Blinding is not possible, because of the type of intervention. It is impossible to create a convincing placebo for the workshop on decluttering and organizing, and this lack of blinding may affect the results. As far as possible, subjects need to have an experience equivalent to undergoing training from a professional organizing and cleaning service.

Acknowledgments

We are grateful for advice from Yukiko Mochizuki, Yuka Nojiri, and Mihoko Shimozono about preparing a study protocol.

The text for the workshop was prepared with help from Keiko Takayama and Mayumi Takahara.

Contributors

YA conceived the study. KY and NA participated in the development of the protocol. MS and YN contributed to the study conception and design of the education program. All authors prepared and revised the manuscript, including relevant scientific content. All authors approved the final version of the manuscript.

Funding

This work was supported by JSPS KAKENHI Grant Number JP26671045 (2014–2017) Grant-in-Aid for Scientific Research on "The state of young people who chronically fail to organize or tidy up and development of a program for effective intervention" (Challenging Exploratory Research, Study Organizer: Yasuko Aso, grant no. 26671045). This funding source had no role in the design of this study and will have no role during

For peer review only - http://bmjopen.bmj.com/site/about/guidelines.xhtml

its execution, analyses, interpretation of the data, or in any decision to submit results.

Competing interests statement

No financial assistance or free services will be received from any company or organization during this study. The researchers have no equity, such as stock, in any of the companies involved, and are not involved in their management, nor have the researchers received any services, free goods or pharmaceuticals, or discounts from those companies.

Subjects' consent

Obtained.

Ethics approval

This trial has been approved by Teikyo University Review Board 15-065
Protocol version 2. Issue date 23 January 2017, Authors: *YA, KY, NA.*

Data sharing statement

The informed consent documents are available on request.

REFERENCES

1. American Psychiatric Association.American Psychiatric Association. Diagnostic and statistical manual of mental disorder (DSM). Washington, DC: American Psychiatric Association 1994:143–7.
2. Frost RO, Steketee G, Grisham JR. Measurement of compulsive hoarding: saving inventory-revised. *Behav Res Ther* 2004;42(10):1163–82.
3. Tolin DF, Frost RO, Steketee G, et al. The economic and social burden of compulsive hoarding.*Psychiatry Res* 2008;160(2):200–11.
4. Ministry of Land, Infrastructure.Transport and Tourism in japan. Understanding of the actual condition of land use bringing marked annoyance to the area (external uneconomic) Questionnaire results2009; http://tochi.mlit.go.jp/wp-content/uploads/2015/07/9fd5e7d0af1a3defbe2ede833715 78d3.pdf(accessed 26 Feb 2017)
5. Kishi E, Nojiri Y, Yonezawa J, et al. An analysis of forms of intervention by nurses at comprehensive community support centers in cases of self-neglect. *Journal of the Japan Academy for the Prevention of Elder Abuse* 2014;10(1):106–20.
6. Frank H, Stewart E, Walther M, et al. A. Hoarding behavior among young children with obsessive–compulsive disorder.*Journal of obsessive-compulsive and related disorders* 2014; 3(1): 6-11.
7. Samuels JF, Bienvenu OJ, Riddle MA, et al. Hoarding in obsessive compulsive disorder: results from a case-control study. *Behav Res Ther*2002;May;40(5):517–528.[PubMed]
8. Frost RO, Krause MS, Steketee G. Hoarding and obsessive-compulsive symptoms.*Behavior Modification*1996;20:116–132. [PubMed]
9. Rasmussen SA, Eisen JL. Clinical features and phenomenology of obsessive compulsive disorder. *Psychiatric Annals* 1989;19:67–73.
10. Sobin C, Blundell ML, Weiller F, et al. Evidence of a schizotypy subtype in OCD.*Journal of Psychiatric Research* 2000;34:15–24.[PubMed]
11. Saxena S, Maidment KM, Vapnik T, et al. Obsessive-compulsive hoarding: symptom severity and response to multimodal treatment.*J Clin Psychiatry*2002 Jan;63(1):21–27.[PubMed]
12. Timpano K R, Raines A M, Shaw A M, et al. Effects of a brief anxiety sensitivity reduction intervention on obsessive compulsive spectrum symptoms in a young

For peer review only - http://bmjopen.bmj.com/site/about/guidelines.xhtml

adult sample.*Journal of Psychiatric Research* 2016;83, 8-15.
13. Grisham JR, Frost RO, Steketee G, et al. Age of onset of compulsive hoarding. *J Anxiety Disord* 2006;20(5):675–86.
14. Tolin, D. F., Meunier, S. A., Frost, R. O, et al.Course of compulsive hoarding and its relationship to life events. *Depression and Anxiety*, 2010;27(9):29-838.
15. Timpano, K. R., Exner, C., Glaesmer, et al.The epidemiology of the proposed DSM-5 hoarding disorder: exploration of the acquisition specifier, associated features, and distress. *The Journal of clinical psychiatry,*2011; 72(6), 780-6.
16. Nordsletten AE. Epidemiology of hoarding disorder. *Br J Psychiatry* 2013;203:445–52.
17. Tolin DF, Frost RO, Steketee G. Cognitive behavioral therapy for hoarding disorder: A meta-analysis. *Depress Anxiety* 2015;32(3):158–66.
18. Steketee G, Frost R O, Tolin D F, et al. Waitlist - controlled trial of cognitive behavior therapy for hoarding disorder.*Depression and anxiety* 2010;27(5):476-484.
19. Frost R O, Ruby D, Shuer L J.The buried in treasures workshop: waitlist control trial of facilitated support groups for hoarding. *Behaviour research and therapy* 2012; 50(11), 661-667.
20. Aso Y, Masashige S, Yuka N, et al. Attitudes of undergraduates studying nursing or welfare work towards organizing and tidying up and the state of their living spaces. [abstract]. The 74th Annual Meeting of the Japanese Society of Public Health 2015;p294.
21. Aki T, Ken-ichi K, Toko I, et al. A consideration of clinical characteristics of non-clinical hoarding among Japanese adolescents. *Japanese Society of Anxiety and Related Disorder* 2015;6(2):72–85.
22. Kazue Y, Toshiro T. "Effects of lifestyle modification on metabolic syndrome: a systematic review and meta-analysis.*BMC medicine* 2012; 1
23. Anderson L M, Quinn T A, Glanz K, et al. The effectiveness of worksite nutrition and physical activity interventions for controlling employee overweight and obesity: a systematic review.*American journal of preventive medicine* 2009;37(4):340-357.
24. Japan Association of Life Organizers.Fun Cleansing for Elementary School Children - Life Organization to Start at the age of 10,Tokyo:Japan Association of Life Organizers2015.
25. Storch E A, Muroff J, Lewin A B, et al. Development and preliminary psychometric evaluation of the Children's Saving Inventory.*Child Psychiatry & Human Development* 2011;42(2): 166-182.
26. http://www.compareyourcountry.org/pisa/country/oecd (accessed 20 Feb 2017).
27. Frost RO, Steketee G. Compulsive Hoarding and Acquiring: Therapist Guide. Japanese edition. Toko I, translator. Tokyo: Kanekoshobou 2013:170–3.
28. Frost RO, Steketee G, Tolin DF, et al. Development and validation of the Clutter Image Rating. *J Psychopathol Behav Assess* 2008;30(3):193–203.
29. MarikoY, Yutaka M, Yukiko Y. The Structure of Perceived Aspects of Self. *Japanese Association of Educational Psychology* 1982;30(1):64–8.
30. Tomari S, Yoshida F. Psychological review of meanings of private space and its privacy research and proposal for functions: new models. *Tsukuba Psychological Research* 1998;20:173–90.
31. Kauffman, R. E., Banner Jr, W., & Berlin Jr, C. M. (1995). Guidelines for the ethical conduct of studies to evaluate drugs in pediatric populations. *Pediatrics*, *95*(2), 286-294.
32. Modi, N., Vohra, J., Preston, J., Elliott, C., Van't Hoff, W., Coad, J., ... & Greenough, A. (2014). Guidance on clinical research involving infants, children and young people: an update for researchers and research ethics committees. Archives of disease in childhood, archdischild-2014

For peer review only - http://bmjopen.bmj.com/site/about/guidelines.xhtml

FIGURE TITLES

Figure 1. Trial design

SUPPLEMENTAL FILES

File 1. Explanation Document (The Subjects themselves from 12 years old to 14 years old)Japanese version

File 2. Explanation Document (The Subjects themselves from 12 years old to 14 years old)

File 3.Explanation Document (The subjects themselves of 15 years old or older_Parents_Those who live together) Japanese version

File 4.Explanation Document (The subjects themselves of 15 years old or older_Parents_Those who live together)

File 5. Consent Form (Subject themselves from 12 years old to 14 years old) Japanese version

File 6. Consent Form (Subject themselves from 12 years old to 14 years old)

File 7. Consent Form (Subjects themselves from 15 years old or older and parents and those who live together Japanese version

File 8. Consent Form (Subjects themselves from 15 years old or older and parents and those who live together

For peer review only

For peer review only - http://bmjopen.bmj.com/site/about/guidelines.xhtml

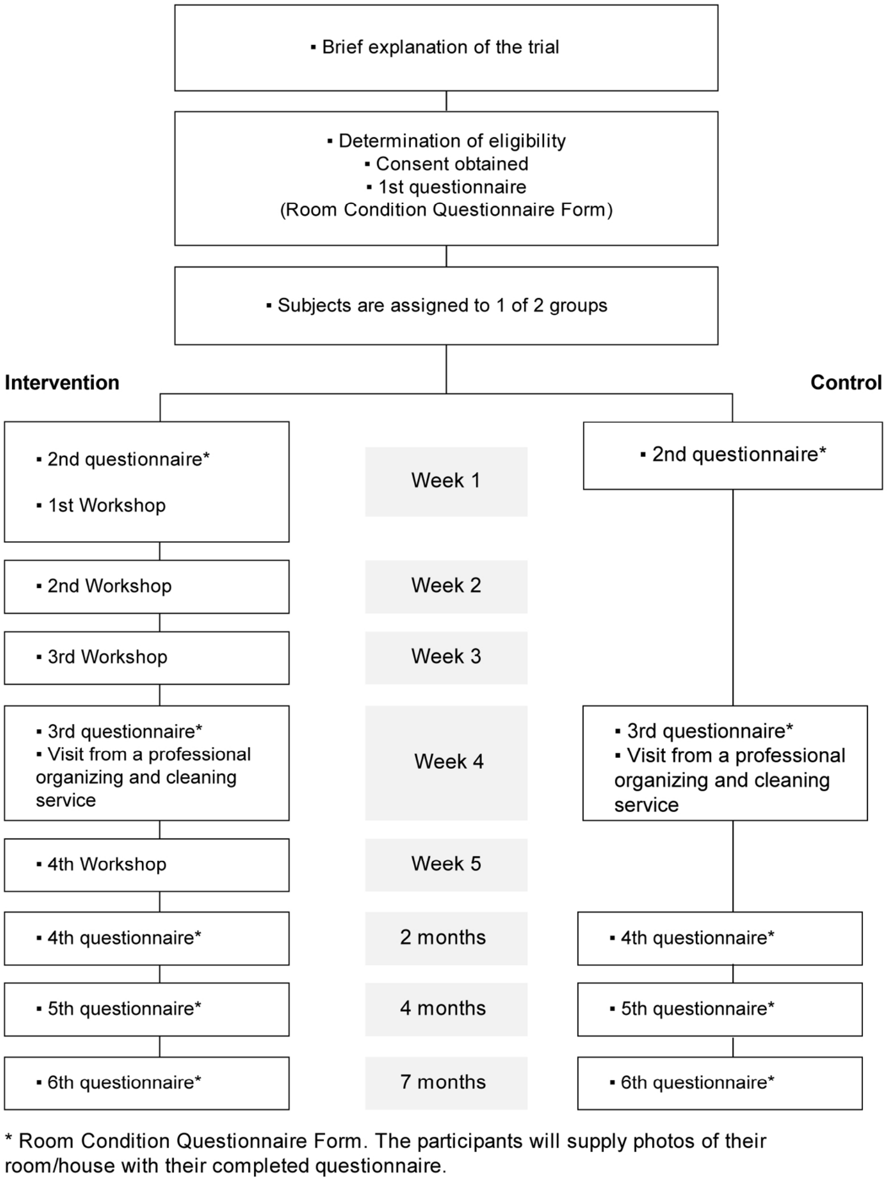

Figure 1. Trial design

140x177mm (300 x 300 DPI)

研究参加者への説明文書（12 歳～14 歳のご本人用）

研究名「整理整頓が苦手な人向けの“片づけ教室”の効果を調べる研究（くじ引きで、片づけ教室を受けるか、受けないかを分けることによって片づけ教室の効果を見る研究)」への参加をお願いしたく、その内容を説明いたします。この研究へ参加するかどうかは、説明を聞いたうえで、あなたが自由に決めることができます。いつでも質問に応じますし、いったん決めた後でも取り消すことができます。ただ、研究を受けていただくことの条件が合わない場合、こちらから参加をお断りする場合もあります。

□研究の目的・意味

この研究の目的は、整理整頓が苦手な 12 歳から 54 歳までの人に「片付け教室+専門家の訪問による片づけ」を行うチーム(教室あり群)と、「専門家の訪問による片づけ」だけを実施するチーム（教室なし群）にくじ引きで分け、調査前、調査開始すぐ、1 か月後、2 カ月後、4 か月後、7 か月後にあなたのお部屋の使いやすさ、片づけが苦手かどうかなどについてアンケートで伺い、「片付け教室」の効果を見ます。どちらの群になるかは、くじ引きで決めるため、あなたがどちらになるかはわかりません。

若い時に整理整頓や片づけがとても苦手な人が年を取った時に、さらにお部屋が散らかっている人が多いことが分かっています。また、片づけや整理整頓の方法は、学校や本で勉強することができますが、片づけ教室の先生から学ぶことで整理整頓が上手になることが考えられます。今回の研究の結果をたくさんの人に伝え、将来、「片付け教室」が、いろいろなところで実施されるようになることによって、片づけが苦手な人のお部屋がきれいになり、生活がしやすくなることを目指します。

□調査を受ける人の集め方と調査の方法

応募をしてくれた人へ、「お部屋の状態を聞くアンケート」を行い、お部屋が散らかっていたり、片づけが苦手な人を調査を受ける人とします。ただし、病気やケガ、障害などで、自分ではお部屋の片づけができない人は、今回の調査は受けられません。調査を受ける人を教室ありチームと教室なしチームにクジで分け、教室ありチームには「4 回の片付け教室+1 回の専門家の訪問による片づけ」を行います。教室なしチームには、「1 回の専門家による片づけ」だけを行い、片づけ教室は行いませんが、調査期間が終わった後に希望する場合は「片づけ教室」を受けることができます。両方のチームについて、あなたと専門家で一緒にあなたのお部屋の片づけを行い、その後のお部屋の片づけの回数や状態、自分のことが好きかどうかなどについてなどのアンケートと、あなたが撮影したお部屋の写真を見て、片づけ状態を判定し、教室ありチームと教室なしチームを比べます。あなたは定期的にアンケートに答えたり、お部屋の写真をメールや郵送で送る必要があります。

この調査に参加することによりあなたのお部屋は整理整頓された状態になりますが、その後、時間がたつにつれ、お部屋がまた、散らかった状態に戻ってしまう可能性はあります。この調査に参加しない場合や途中で参加をやめた場合でも、希望があれば片づけ方法について書かれた本や相談できる場所をお知らせすることができます。

For peer review only - http://bmjopen.bmj.com/site/about/guidelines.xhtml

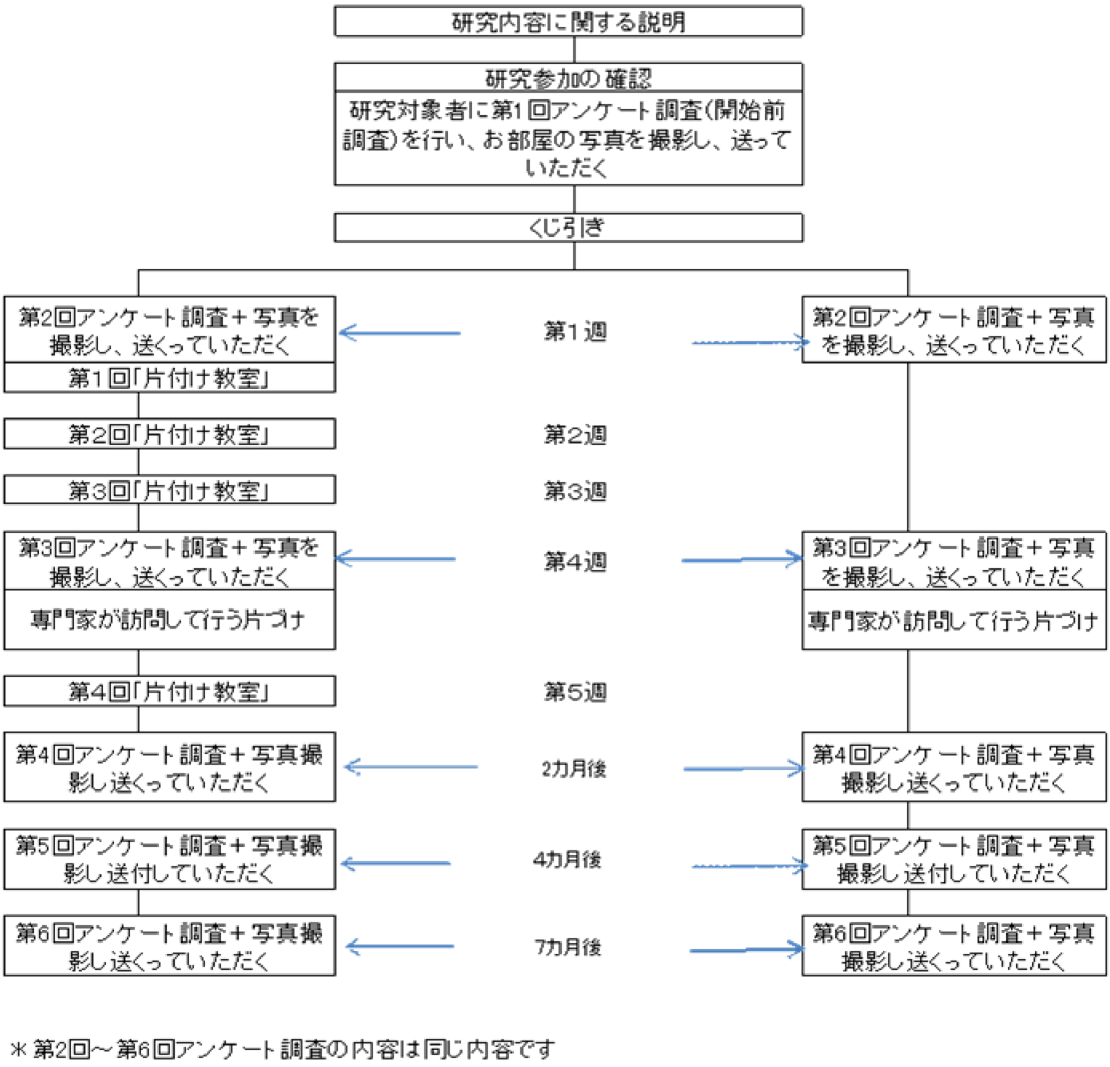

図１　研究全体の流れ

□参加をやめたいとき

この研究に参加するかどうかは、あなたが自由に決定し、参加しなくても、また、途中で参加をやめても、損をしたり、つらい目にあうことはありません。また、調査が始まった後や、調査が終わった後に、研究への参加をやめたいと思った時は、全員のアンケート結果をまとめる前であれば、あなたのデータを消すことができ、その事により、あなたが損をすることや困ることはありません。

研究に参加しないと決めた場合や、途中でやめることにした場合でも、研究結果を知りたい場合は、連絡をいただければ、結果についてご報告を致します。

□研究を行う人

研究を担当する人は以下の通りです。

＜研究代表者＞

＜担当者＞

For peer review only - http://bmjopen.bmj.com/site/about/guidelines.xhtml

＜研究協力者＞

＜研究事務局＞

□研究の場所・期間

「片づけ教室」は帝京大学内で行い、専門家の訪問による片づけ・整理整頓作業は、あなたのお部屋であなたと専門家が一緒に行います。

研究開始の許可が下りてから参加する人を募集し、調査開始後 7 か月後までが、あなたにアンケート調査を行う期間ですが、すべての人の調査が終了する時期は、2017 年 3 月までとなります。

□研究で使う資料と情報の取り扱い

調査によって集まったデータは番号が付けられ、誰のものかわからないようになっています。データの扱いは、パソコンに保管してパソコンには他の人が勝手に開けられないよう、パスワードをかけます。紙に書いた情報はデータをパソコンに入力後、お部屋の写真は、判定した乱雑レベルを入力後すぐにシュレッダーで細かく切って内容が分からないようにします。

また、研究代表者は、研究で使用した書類（倫理委員会からのお知らせ文書、申請書・報告書などのコピー、対象者番号が書かれた物、同意書、報告書等のコピー、その他必要な書類）などは保存し、研究発表後 5 年後に捨てます。研究がきちんと行われているかチェックされる場合や、上に書いた研究メンバーが行う将来の研究で使用する時も、データからどの人の内容かが特定されることはありません。

あなたのご自宅に専門業者が訪問する時は、あなたの個人情報が守られるように、文書で専門業者と約束を取り交わします。また、あなたの個人情報が守られるよう、きまりを守るように求めます。

□研究結果の扱い

研究結果は、学会等へ発表することと帝京大学への提出を予定しています。また、片づけ教室のテキストなどは、公表する予定ですが、あなたの個人情報や個人データが広まることはありません。

□研究に必要な費用の出どころ

本研究は平成 26～29 年度「慢性的に片づけられない若年者の実態と効果的介入プログラムの開発」（科学研究費補助金：挑戦的萌芽研究　研究代表者　麻生保子、課題番号 26671045）の研究として行われ、そちらから費用を使用します。

□損・得に関わる行為

この研究に関して、特定の企業や団体から資金はもらっていません。また、この研究によって得をする人と損をする人がいるかどうかについては、「帝京大学板橋キャンパス利益相反管理委員会」で確認してもらっています。また、この調査に参加していただくこと事により、整理整頓方法を学ぶことはできますが、参加費は不要です。アンケート調査や写真を送付していただいた時に、お礼として 200 円～500 円程度の文房具またはクオカード等を差し上げます。

For peer review only - http://bmjopen.bmj.com/site/about/guidelines.xhtml

□研究参加の費用等について

「片づけ教室」参加費用や、専門家によるお部屋の片づけ作業の費用、専門家があなたの自宅へ行く時の交通費等をあなたが支払う必要はありません。ただし、お部屋の片づけをする時に、物品をしまう戸棚類をあなたやあなたの保護者が買うことを決めた場合は、あなたや保護者の方が支払うことになります。また、あなたの教室参加の際の交通費およびお部屋の写真撮影やデータを送る時にかかる費用はあなたや保護者の方に支払っていただく事になります。

□研究に参加中にあなたに困ることが起きた際の対応

調査中に体調の悪化やいやな気持ちになった時などは、すぐに調査を中止します。調査中にあなたが何らかのケガや病気になった場合は、研究チームが対応し、大学の保健室へ一緒に行きます。あなたのお部屋の片づけの時に、家具類が壊れたり、あなたやご家族の健康に害があると考えられることがあった場合は、片づけの専門家が入っている損害保険により対応しますが、健康保険での対応となります。担当者の言葉がけや態度、作業内容により、あなたがいやな思いをした場合は、下記の連絡先に連絡してください。

□研究中止の条件

１．あなたが以下の条件となった際には、調査を中止します。

1) あなたや、保護者、一緒に住んでいる方が調査参加をやめると決めた場合
2) 調査の対象者でないことが解った場合
3) あなたの健康や生活にとっての損や不都合が大きく、調査を続けることが難しい場合
4) あなたの体調等により調査を続ける事が良くないと判断された場合
5) あなたがかかっている病気の悪化により調査が難しい場合
6) 調査研究全体が中止された場合
7) その他の理由により研究代表者が調査研究を中止した方が良いと決めた場合

２．調査自体が中止となる条件

1) 調査の安全性や効果がないのではないかと考えられる情報が得られたとき。
2) 調査に参加してくれる人を探す事が難しく予定の人数を探すことが不可能と考えられる時。
3) 予定調査数または予定期間になる前に、調査の目的が達成されたとき。

＊以上の事があった場合は、直ぐに大学内の倫理委員会で話し合い、検討結果をご連絡します。

□質問への対応の仕方・連絡先

研究計画書や研究の方法に関する資料を見たり欲しい場合や質問がある場合は研究代表者に連絡し、資料を見たり、もらったり、質問することができます。連絡先は次の通りです。

説明日：　　年　　月　　日

説明者：

For peer review only

Explanation Document for Research Participants
(Individuals from 12 years old to 14 years old)

We would like to ask for your participation in, and explain the research on the effect of the "Decluttering and Organizing Class" for people who have difficulty in organizing (separating participants into groups of those with or without class sessions by drawing in order to fairly monitor the effectiveness). You can decide whether you want to join or not after reviewing detailed information on the study. We will address your questions at any time, and you can cancel your participation even after agreeing to participate. If you do not meet our research requirements, we may not be eligible for participation in the study.

□ Purpose and meaning of the research

The purpose of this research is to examine the effect of the "Decluttering and Organizing class" by separating the participants into two groups by drawings: one for those who will attend the Decluttering and Organizing Class as well as an organizing work visit at home (the group with class) and the other for those who conduct an organizing work visit at home for those who have difficulty in organizing between the age of 12 and 54. We will ask questions about the functionality of your room and whether you can organize the room after one month, two months, four months, and seven months. Which group you belong to will be decided by a drawing.

When people have difficulty in organizing at a young age, they tend to be more disorganized when they grow older. Also, while people can learn organizing methods at school or by reading books, they can learn more by working hands on with professionals in organizing classes or workshops. With our research findings, we hope to see more decluttering and organizing classes available for those who are disorganized so that their lives will be a lot easier.

□ How to recruit participants for the research; how the research will be conducted

We will ask the candidates to fill out the "questionnaire about conditions of the rooms", and select people whose rooms are cluttered, or who have difficulty organizing. However, those who are disorganized due to illness, injury, or disabilities cannot participate. The participants will be separated into groups by drawings: one with the class, and one without the class. For the group with the class, 4 "Decluttering & Organizing class sessions and 1 organizing work session at home by organizing professionals" will be offered. For the group without the class, they will have only 1 organizing work session with professionals so that we can measure whether conducting the class is effective or not. In both groups, the organizing professionals will conduct the decluttering & organizing work sessions with the participants. We will ask questions to total of 60 people from both groups, about the number of organizing work sessions conducted, room conditions, and whether you feel positive about yourself or not, and compare the group with the class and the

For peer review only - http://bmjopen.bmj.com/site/about/guidelines.xhtml

group without the class. The participants need to answer questionnaires periodically, and send the photos of their rooms via email or a regular mail.

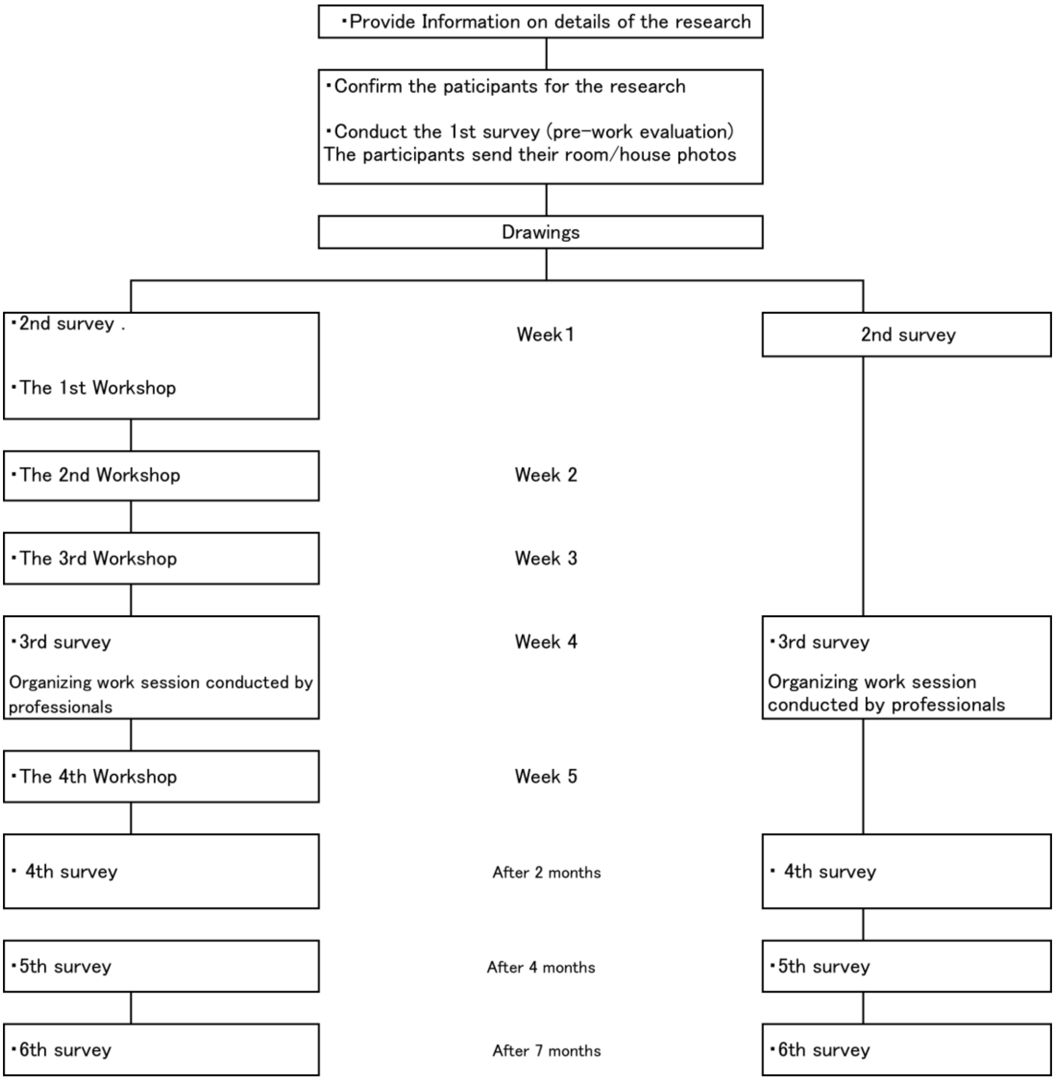

＊The contents of the 2nd through the 6th questionnaire are the same. The participants send their room/house photos when surveyed .

☐ If you want to quit participating

You can freely decide whether you would like to participate in the research or not, and even if you don't participate, you won't lose anything or be at a disadvantage. Also, after the research has started, or has been completed, if you think you want to quit participation, we can delete your data if it is before we compile the findings of the questionnaire. You won't lose anything because of your withdrawal.

In the event that you decide not to participate in the research or chose to withdraw, but you

For peer review only - http://bmjopen.bmj.com/site/about/guidelines.xhtml

would like to know the research findings, you can still obtain that information by contacting us.

□ Who will be conducting the research?

<Research representatives>

< Research members>

< Associate Researchers>

<Research administrative>

☐ Location and timeframe of the Research

We will host “the Decluttering & Organizing Class” at Teikyo University. Organizing professionals will visit your home to conduct organizing work sessions with you.

When the research is approved and ready to proceed, we will start recruiting for participants. Seven months after the start will be the end of your (the participants) evaluation period.

☐ Handling of research materials and personal information

Data collected for research will be numbered, so no one will know whose data it is. Then the data will be stored in a computer, which is secured with passwords, so unauthorized people cannot access the computer. Any information on paper will be shredded and destroyed immediately after they are entered in the computer, and photos of your rooms will be shredded after the review.

Also, the research representative chief securely stores documents used for research (announcements from the ethics committee, copies of applications and the reports, documents containing the participants numbers, consents copies of the reports, and other necessary documents), and he or she deletes them after five years. If we check the data in the future to see if the research was done properly or revisit the data for other research projects, we will not be able to identify whose data it is.

When the organizing professionals visit your home, we will sign an agreement with them to not disclose any of your personal information. Additionally, we will ask them to comply with the rules so your personal information is securely protected.

☐ Handling Research Results

We plan to submit and publish the research findings to Teikyo University and academia. Also the

For peer review only - http://bmjopen.bmj.com/site/about/guidelines.xhtml

class materials for the Decluttering and Organizing Class will be released; however personal information and individual data will not be shared.

□ Funding for the research

This research will be done as a part of the study "The reality of young people who are Chronically Disorganized; program development for the effective intervention" (From 2014 to From 2017) (The research subsidy: Challenging *Houga* (Bud flush) Research, Research Representative, Yasuko Aso, Research Number 26671045)

□ Conflicts of interest

We don't receive any funds from specific corporate sponsors or organizations for this research. Also, we will have potential conflict of interest of the research screened by the Committee by Itabashi Campus of Teikyo University. You can gain knowledge about organizing by participating in this research, but you don't have to pay for it. Also, you will not earn any compensation for participating in this research.

□ Cost for research participation

You are not required to pay for participating in the Decluttering and Organizing Class. You are also not required to pay for the organizing work sessions and any expenses for the vendor's transportation. However, in the event that the participants (or their parents / guardians) need to purchase closets to store goods when they conduct the organizing work and decide the purchase it, the participants will have to pay for them. It is the participants' responsibility to pay for any transportation to attend the class, and the cost of mailing their room photos.

□ How to address adverse events

If your health condition becomes worse or you do not feel well, we will stop the evaluation immediately. If you need to seek medical attention, the medical staff members in the research group will respond accordingly. We will then take you to the nurse's office at the University. If a piece of furniture or other items at your home are damaged, or you or your family's health is affected during the organizing work, the liability insurance policy of the vendor will address the issue, but your health insurance is also needed. If you are uncomfortable with words, attitudes, or actions of the workers during the research period, please contact the research members or the following research administrator.

□ Conditions to stop research

１. If you fall under the following conditions, the evaluation will be cancelled.

1) If you or your parents or those who live together with you wish for your withdrawal from

For peer review only - http://bmjopen.bmj.com/site/about/guidelines.xhtml

the research

2) If it is determined that you are not eligible for the research
3) If you suffer a large serious setback to your health and lifestyle, and have difficulties continuing the evaluation.
4) If it is decided that continuing the research is not recommended due to health, etc. ,
5) If you develop complications from an illness, and have difficulty continuing the evaluation,
6) If the entire research program is cancelled.
7) If the research representative decides that it is appropriate to stop the examination due to other reasons

２． Conditions under which the entire research program would be cancelled.

1) Significant information about a lack of safety and effectiveness in the research is reported
2) It is decided that recruiting participants for the study is difficult and it interferes with the schedule.
3) The research purpose is achieved before the scheduled date or goal of the evaluation is achieved.

* If any of the above matters are observed, the internal screening committee and ethics screening committee at the university will discuss the matters and the decision will be announced.

□ How to address questions and contact information

If you wish to obtain the materials regarding the research plan or the method, to access them, or to ask questions, please contact the research representative. You will be provided with access, and obtain information or an opportunity to ask questions about the materials.

Please contact:

Date of Explanation ： Year Month Date

Explained by ：

＊ （English version (actually using Japanese version)

For peer review only - http://bmjopen.bmj.com/site/about/guidelines.xhtml

研究参加者への説明文書（15 歳以上のご本人・保護者または同居の方用）

研究名「整理整頓が苦手な若年者を対象とした、無作為割り付けによる “片づけ・整理整頓教室”の効果に関する研究（くじ引きで教室をする群としない群を分け、教室の効果を見る研究)」の参加を依頼したく、その概要を説明いたします。この研究への参加について下記の項目に従い、十分な説明をいたしますので、よく理解された上で、あなた(調査研究対象の方）の自由意思により参加するか否か決めてください。いつでも質問に応じますし、いったん決めた後でも取り消すこともできます。この際に不利益はありません。ただ、条件が合わない場合、こちらの方から参加をお断りする場合もあります。

□研究の目的・意義

本研究の目的は、整理整頓が苦手で、かつ、アンケート調査により居室状態が比較的乱雑であると判定された方を対象とし、くじ引きにより「片付け・整理整頓教室+家庭訪問による整理整頓作業」を実施する群(教室実施群)と、「家庭訪問による整理整頓作業」のみを実施する群（教室なし群）に分けて、教室の効果を見ることを目的としています。尚、研究対象者の方がどちらの群になるかを選択することはできません。両群の比較は、調査前、調査直後、1 か月後、2 カ月後*、4 か月後、7 か月後に対象者の居室状態と生活の質、整理整頓への知識や苦手意識、自尊感情等について評価し、「片付け・整理整頓教室」の効果を比較します。極端に居室状態が乱雑な人は、加齢により体力の衰えとともに、整理整頓能力が衰えることが分かっています。昨今、いわゆる「片付け本」に沿って、自分で整理整頓方法を学ぶ人も増えていますが、片づけ支援教室による学びと、効果的な整理整頓作業は、片付けが苦手な人にとっては有効であることも、既存の研究で公表されています。本調査結果を広く公表し、将来、効果的な「片付け・整理整頓教室」が、広く行政や片付け支援業者によって実施されるようになることによって、整理整頓が苦手な方の生涯にわたる生活の質の向上に寄与することを目指します。

□研究の対象と方法

公募等による応募者へ質問紙により事前調査を行い、研究代表者が以前行った調査結果（2 大学 450 人の調査結果）の平均点より高かった人のうち、自力で整理整頓作業を行うことができる人を今回調査の対象者とします。対象者を教室実施群と教室なし群にくじ引きで分け、教室実施群には「4 回の片付け・整理整頓教室+1 回の家庭訪問による整理整頓作業」を行います。教室なし群には、「1 回の家庭訪問による整理整頓作業」のみを行い、教室の効果を比較します。教室なし群には「片づけ・整理整頓教室」は行いませんが、調査終了後、希望があれば「片づけ・整理整頓教室」を実施します。両群の比較に当たっては、写真による居室状況の把握の他、居室状況に関する調査票や、自尊感情尺度得点、整理整頓への知識や苦手意識、直近 1 週間の整理整頓状況（整理整頓の頻度、居宅内環境活用状況)、直近 1 週間の自宅への来訪者数等を伺います（以下、これら一連の調査を「アンケート調査」とします)。また、調査開始前に同居者数や整理整頓の頻度、居室の数と広さ他、整理整頓状況に関わる質問や、あなた(調査研究対象の方）の状況がわかる、年齢、学歴、勤務形態等に係る研究開始前調査を行います。調査実施に当たり、定期的に居宅状況に関するアンケート調査への回答やご自宅の写真をメールか郵送で送付していただく必要があります。(図 1)本研究参加による、専門家があなたのご自宅を整理整頓することにより一時的にあなたのお部屋は整理整頓されますが、時間の経過とともに、再度、お部屋が乱雑状態になる可能性はあります。本研究に参加しない場合や途中で参加を取りやめ

For peer review only - http://bmjopen.bmj.com/site/about/guidelines.xhtml

た場合でも、ご希望があれば片づけ方法について書かれている資料や、状態に応じた相談機関をご紹介することができます。

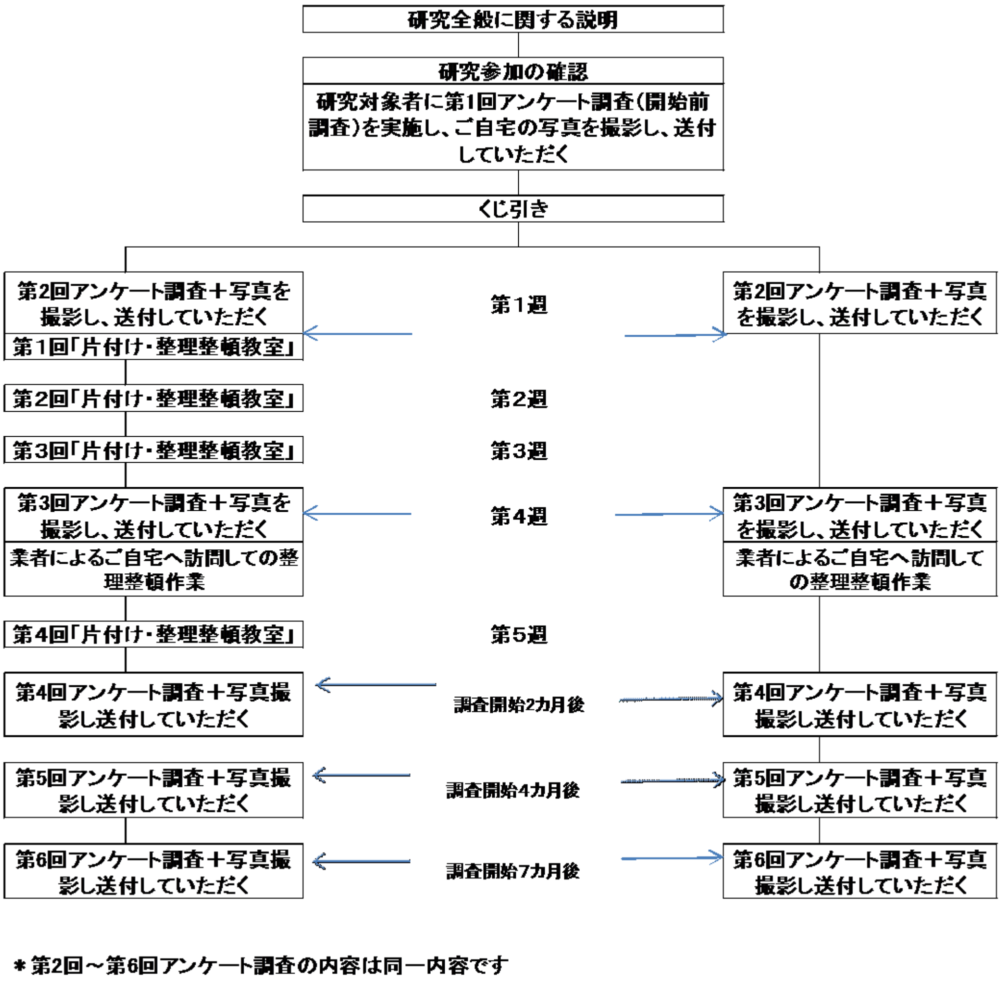

図１　研究全般の流れ図

□研究への自由意思参加・同意取消しの自由

本研究への参加は全くの自由意思で決定し、調査への参加の拒否をしても不利益を被ることはありません。また、調査の途中で参加を中止する場合も、不利益を被ることは一切ありません。調査が行われた後においても同意を取り消すことができます。調査開始または終了後に同意を取り消した場合、データが統計解析前であれば、データの削除を行います。また、同意を取り消すことにより不利益は生じません。研究への参加に同意されなかった場合または途中で参加を取りやめた場合であっても、希望があれば研究結果をご報告いたします。

□研究の責任者・組織

本研究は帝京大学を主とした多施設共同研究です。研究組織は以下の通りです。

For peer review only - http://bmjopen.bmj.com/site/about/guidelines.xhtml

＜研究代表者＞

＜研究担当者＞

＜連携研究者＞

＜研究事務局＞

□研究の場所・期間

「片づけ・整理整頓教室」は帝京大学内で開催し、家庭訪問による整理整頓作業は、あなた(調査研究対象の方）のご自宅に専門業者が訪問して行います。

研究の実施期間は帝京大学倫理審査終了後から参加者を募集し、調査開始後 7 か月後までが、あなた(調査研究対象の方）の調査実施期間ですが、最終調査終了時期は、2017 年 3 月までとなります。

□研究試料と情報の取り扱い

調査によって得られた居室の写真を含むデータは ID 番号によって管理され、個人が特定される事はありません。データの取り扱いは、パソコンに保管してパソコンには重層的にセキュリティーをかけ、紙媒体の情報はデータ入力後、居室の写真は、判定した乱雑状態レベルを入力後速やかに裁断します。また、研究代表者は、調査等の実施に係わる文書（申請書類の控え、倫理委員会からの通知文書、各種申請書・報告書の控、対象者識別コードリスト、同意書、報告書等の控、その他データの信頼性を保証するのに必要な書類または記録などは保存し、研究発表後 5 年後に廃棄します。モニタリング・監査を行う場合の閲覧や、研究組織員が行う将来の研究での使用の際も、ID 番号ごとに得点化されたデータのみを取扱い、データから個人が特定されることはありません。

ご自宅に専門業者が訪問する際の個人情報保護に関して、収集する情報と管理、保護に関し別紙誓約書を取り交わし、研究対象者の個人情報が保護されるよう、規則を遵守するよう求めます。

□研究結果の扱い

研究結果および得られた知見は学術学会や帝京大学公衆衛生大学院特殊研究報告書への提出を予定しています。学会発表や論文投稿、報告書記載に当たり、個人情報および個人データが公表されることはありません。

□研究資金源

本研究は平成 26～29 年度「慢性的に片づけられない若年者の実態と効果的介入プログラムの開発」（科学研究費補助金：挑戦的萌芽研究　研究代表者　麻生保子、課題番号 26671045）の研究の一環として行われます。

□利益相反

本研究の実施に際しては特定の企業や団体からの資金援助は受けておりません。また、本臨床研究の利益相反関係は、帝京大学板橋キャンパス利益相反管理委員会の審査を受けております。

□研究参加に伴う負担や支払いの有無

「片づけ・整理整頓教室」参加費用および業者による居宅の整理整頓作業代金、整理整頓作業者の対象者自宅への交通費等への対象者の負担はありません。但し、居宅内整理整頓作業を実施する際に、物品を収納する戸棚類が必要となった場合で、対象者が購入等を決定した場合は、対象者の自己負担とします。また、教室参加の際の交通費および居室の写真撮影、データ送付に要する費用は対象者の自己負

For peer review only - http://bmjopen.bmj.com/site/about/guidelines.xhtml

担となります。居宅状況調査や写真データを送付していただいた際に 200 円から 500 円程度の文房具またはクオカードの謝礼を予定しております。

□被る可能性のある個人の利益、不利益、人的トラブルを含む有害事象とその対応

研究に参加することで、整理整頓方法について、教室または居宅内整理整頓作業にて専門家より学ぶ機会を得ることができます。また、将来の「溜め込み症」予防の方法論確立の進歩に貢献できる可能性があります。調査中に体調の悪化や著しい情動の変化等が認められた際は、即座に調査を中止します。万が一何らかの医療的措置が必要な事態が大学内で生じた場合は、研究チーム内の医療従事者が適宜対応し、大学内保健室へ同行します。対象者の居宅内整理整頓作業に伴う、家財等の破損や、整理整頓作業による健康被害が認められた場合は、整理整頓業者が加入する損害保険により対応しますが、健康保険の範囲とします。研究期間中の研究者や担当者の言動や態度、配慮に欠けた対応等により対象者が不快と受け取れる事象等があった場合は、研究担当者または下記の研究事務局代表へご連絡ください。

□研究中止の条件

１．あなた(調査研究対象の方）が以下の条件となった際には、調査を中止します。

1) あなた(調査研究対象の方）や、保護者、同居人に方から調査参加の辞退の申し出や同意の撤回があった場合
2) 登録後に調査に適格でないと判明された場合
3) あなた(調査研究対象の方）の健康や生活にとっての不利益が大きく、調査の継続が困難な場合
4) あなた(調査研究対象の方）の体調等により調査継続が好ましくないと判断された場合
5) あなた(調査研究対象の方）の合併症等の増悪により調査実施が困難な場合
6) 調査研究全体が中止された場合
7) その他の理由により研究代表者が調査研究実施を中止することが適当と判断した場合

２．調査研究自体が中止となる条件

1) 研究調査の安全性、有効性に関する重大な情報が得られたとき。
2) 対象者の確保が困難で予定例を達成することが、困難であると判断されたとき。
3) 予定調査数または予定期間に達する前に、調査の目的が達成されたとき。

＊上記の事象が生じた際は、すみやかに大学内の倫理委員会で検討し、検討結果を対象者にご連絡します。

□質問への対応の仕方・連絡先

研究計画書及び研究の方法に関する資料を入手又は閲覧希望の際や質問がある際は研究代表者に連絡し、資料を閲覧・入手、質問できます。連絡先は以下の通りです。

説明日：　　年　　月　　日

説明者：

For peer review only - http://bmjopen.bmj.com/site/about/guidelines.xhtml

Explanation Document for Research Participants

(Individuals 15 years of age or older, Parents or Guardians)

We would like to ask for your participation in, and provide an overview of, the research on the effect of the “Decluttering and Organizing Class” by RCT for young people who have difficulty with organizing (separating participants into groups of those with or without class sessions by drawing in order to fairly monitor the effectiveness). I will explain the research participation fully, based on the following items, so please decide whether you wish to participate (as a participant in the research) or not. We will address your questions or concerns at any time, and you can cancel your participation even after you have signed up for the research. You will not be disadvantaged for any reason due to participation. If you do not meet our research requirements, we may not be eligible for participation.

□Purpose and Meaning of the Research

The purpose of this research is to evaluate the effectiveness of the class, by separating the participants into two groups by drawings: one for those who attend the Decluttering and Organizing Class as well as an organizing work visit at home (the group with class), and the other for those who conduct an organizing work visit at home (the group without class), and targeting those who are determined to have a room condition that is relatively disorganized by answering the questionnaire. Also, the participants of the group cannot select which group they belong to. Both groups will have their room conditions, quality of life, and knowledge and resistance toward organizing, and their self-esteem compared after one month, two months, four months, and seven months, and the effectiveness of the “Decluttering and Organizing Class” will be evaluated.

It is shown that participants whose room conditions are disorderly weaken their ability to organize as their physical energy is weakened. Recently, more and more people are learning how to organize by themselves following so called “tidying up books”. Existing research shows that learning about tidying up in class and effective organizing operations are valuable for those who are not good at tidying up. We aim to contribute to the improvement of the quality of life for the entire life of those who are not good at organizing by widely releasing the results of the research, having an effective “Tidying Up and Organizing Class” held by the governments and vendors that support tidying up.

□ Subjects and Method of the Research

We will conduct the pre-research by sending the questionnaire to applicants through open recruitment, and targeting people who can conduct organizing tasks by themselves out of those whose average score was high in the previous evaluation conducted by the research representative (The findings consist of 450 subjects from two universities.) We will separate the

For peer review only - http://bmjopen.bmj.com/site/about/guidelines.xhtml

participants into two groups: a group with the class and a group without the class by drawing. For the group with the class, 4 Decluttering & Organizing Class sessions and 1 organizing work session at home by organizing professionals will be offered. For the group with no class, they will have only 1 organizing work session by the professionals so that we can measure whether conducting the class is effective or not. In order to compare the groups, we will observe the room conditions by photos, examination forms, scores for self-esteem, and ask about knowledge and resistance toward organizing, the organizing condition in the past week (frequency of organizing, whether they are embracing the housing environment or not), and the number of visitors to their home in the past one week. (The series of research is called “Questionnaire Evaluation.”) Also, we will conduct the evaluation prior to the class, regarding their age, educational background, and work style. It is necessary for you to send the answers to the questionnaire regarding your current house condition periodically, and to also send photos of your home via email or regular mail. (Chart 1)

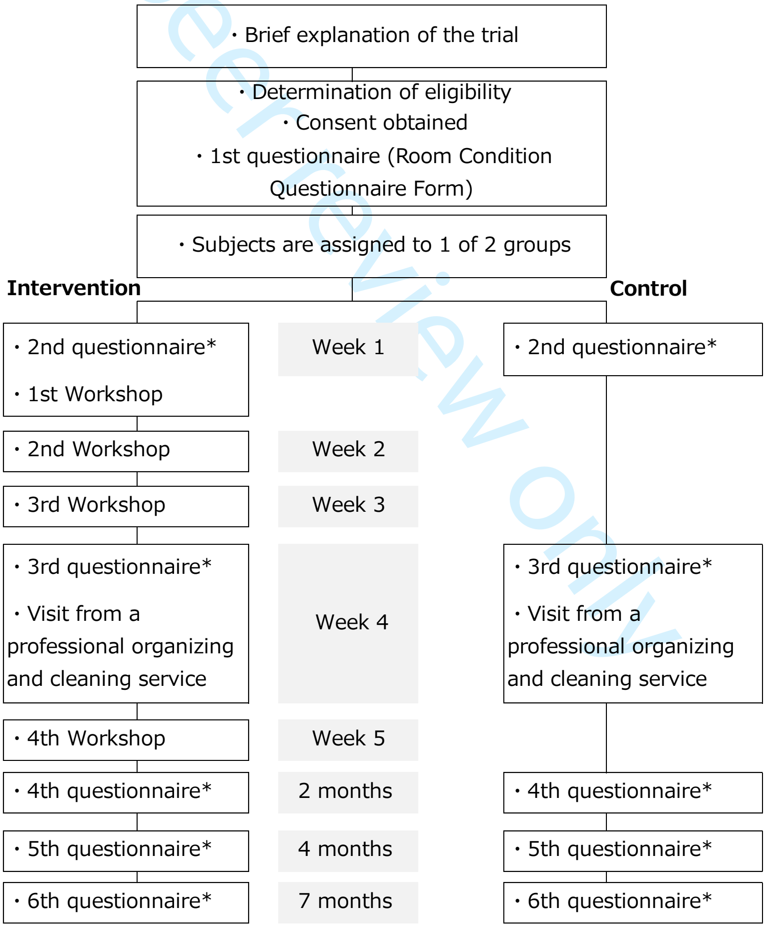

* Room Condition Questionnaire Form. The participants will supply photos of their room/house with their completed questionnaire.

For peer review only - http://bmjopen.bmj.com/site/about/guidelines.xhtml

☐ Voluntary Participation and Right to Withdraw Consent

The participants decide whether they will participate in the research or not of their own will, and won't be at any disadvantage even if they refuse participation in the research. Also, even for participants who want to cancel participation during the research, there will be no disadvantage. The participants can even cancel their consent after the research has been conducted. Furthermore, if the participants cancel their consent at the time of the research commencement or after the research, we will delete the data if the data is not yet analyzed. Also, the participants will not be disadvantaged by cancelling their consent. Additionally, in the event that the participants don't participate or cancel their participation, if they wish, we will provide the research findings to them.

□Persons Responsible for and Organization of the Research

This research involves multiple facilities' common research and is mainly done by Teikyo University. The organization structure is as follows.

<Research representative>

< Research members>

< Associate Researchers>

☐ Location and Timeframe of The Research

We will host the "Decluttering & Organizing Class" at Teikyo University. Organizing professionals will visit your home to conduct organizing work sessions with you.

When the research is approved and ready to proceed, we will start recruiting for participants. Seven months after the start will be the end of your (the participants) evaluation period.

☐ Handling of Research Materials and Personal Information

Data collected for the research will be numbered, so no one will know whose data it is. Then the data will be stored in a computer, which is secured with passwords so unauthorized people cannot access the computer. Any information on paper will be shredded and destroyed immediately after they are entered into the computer, and photos of your rooms will be shredded after the review.

Also, the research representative chief securely stores documents used for the research (announcements from the ethics committee, copies of applications and the reports, documents containing the participants numbers, consent, copies of the reports, and other necessary documents), and he or she deletes them after five years. If we check the data in the future to see if the research was done properly or revisit the data for other research projects, we will not be able to identify whose data it is.

We will pursue compliance to protect the personal information of the people targeted for the

For peer review only - http://bmjopen.bmj.com/site/about/guidelines.xhtml

research by exchanging Commitment Documents on collected information, management and protection regarding personal information protection when the vendor visits the home.

☐ Handling of Research Results

We plan to submit and publish the research findings to Teikyo University and academia. Also, the class materials for the Decluttering and Organizing Class will be released; however personal information and individual data will not be shared.

☐ Funding for the Research

This research will be done as a part of the study “The reality of young people who are Chronically Disorganized; program development for the effective intervention” (From 2014 to From 2017) (The research subsidy: Challenging *Houga* (Bud flush) Research, Research Representative, Yasuko Aso, Research Number 26671045)

☐ Conflicts of Interest

We don’t receive any funds from specific corporate sponsors or organizations for this research. Also, we will have potential conflicts of interest regarding the research screened by the Committee by Itabashi Campus of Teikyo University.

☐ Cost and Payment for Participation

You are not required to pay for participating in the Decluttering and Organizing Class. You are also not required to pay for the organizing work sessions and any expenses for the vendor’s transportation. However, in the event that the participants (or their parents / guardians) need to purchase closets to store goods when they conduct the organizing work and decide to purchase them, the participants will have to pay for them. It is the participants’ responsibility to pay for any transportation to attend the class, and the cost of mailing their room photos.

☐ Adverse Events

If we see the participants feeling worse or experiencing drastic emotional changes, we will cancel the investigation immediately. If they need to have any necessary medical measures, the medical staff members in the research group will respond to them accordingly, and go to the health room at the University. If the participants see any damage to their furniture or health damage caused by organizing work, the vendor's damage insurance will address the issue, but it is limited to the scope of health insurance. If the participants feel discomforted by the language and attitude and uncompassionate acts by the researchers and other staff members during the research period, please contact the research members or the following research administrative representative.

☐ Possible Advantages and Disadvantages for Individuals

For peer review only - http://bmjopen.bmj.com/site/about/guidelines.xhtml

The participants can have the opportunity to learn about organizing by participating in the research, and do not need to pay for participation. Also, they will not be paid for the incentive.

☐ Conditions to Cancel the Research

1. If you (the subject of the research) fall under any of the following conditions, the evaluation will be cancelled.
    1) If you or your parents or those who you live with wish for your withdrawal from the research
    2) If it is determined that you are not eligible for the research
    3) If you suffer a serious setback to your health and lifestyle, and have difficulty continuing the evaluation.
    4) If it is decided that continuing the research is not recommended due to health, etc. ,
    5) If you develop complications from an illness, and have difficulty continuing the evaluation
    6) If the entire research program is cancelled.
    7) If the research representative decides that it is appropriate to stop the examination due to other reasons
2. Conditions under which the entire research program would be cancelled
    1) Significant information about a lack of safety and effectiveness in the research is reported
    2) It is decided that recruiting participants for the study is difficult and it interferes with the schedule.
    3) The research purpose is achieved before the scheduled date or goal of the evaluation is achieved.

* If any of the above matters are observed, the internal screening committee and ethics screening committee at the university will discuss the matter and the decision will be announced.

□ How to Address Questions and Contact Information

If you wish to obtain materials regarding the research plan or the method, to access them, or to ask questions, please contact the research representative. You will be provided with access, and obtain information or an opportunity to ask questions about the materials.

Please contact:

Date of Explanation : Year Month Date

Explained by : ____________________

(English version (actually using Japanese version)

For peer review only - http://bmjopen.bmj.com/site/about/guidelines.xhtml

同意書　（12 歳～14 歳ご本人用）

医学医療技術学部長殿

研究テーマ：「整理整頓が苦手な若年者を対象とした、無作為化比較試験を用いた“片づけ・整理整頓教室”の効果に関する研究（くじ引きで教室をする群としない群を分け、教室の効果を見る研究）」

私は、この研究について書類をもとに、下の内容について説明を受け、十分理解のうえ、自分の意志でこの調査に参加することを決めました。

説明を受けた内容：

☐ 調査の目的・役割
☐ 誰に調査を行うかとその方法
☐ 私が調査への参加や取消しをすることが自由に決められることについて
☐ この調査を行う人とその人が働く場所
☐ 調査の場所・期間
☐ 個人情報の守り方
☐ 調査結果の活用方法
☐ 調査に必要な費用の出どころ
☐ この調査に関わることによる損・得に関わる内容
☐ 私や保護者の負担や支払いについて
☐ 私や保護者が損をすることや困ることが起きた時の対応について
☐ この調査が中止になる時の条件
☐ 質問の仕方・連絡先

平成　　年　　月　　日

本人氏名（直筆）

説明者の所属・部署

説明者の職名・氏名（自署）

印

For peer review only

Consent Form
(For Individuals 12 years old to 14 years old)

Dear Department Head of Medical Technology of Teikyo University

Research Title: Research on the effect of "Decluttering and Organizing Class" using random comparison experiment for the young people who have difficulty in organizing their rooms (separating in groups with or without class sessions by drawing in order to monitor the effects)

I received a document containing the following information from a person who explained the research, and having fully understood it, agree to participate in the research voluntarily.

What was explained to me

- ☐ Purpose and meaning of the research
- ☐ Subjects and method of the research
- ☐ Voluntary participation and right to withdraw consent
- ☐ Personnel responsible for and organization of the research
- ☐ Location and timeframe of the research
- ☐ Handling of research materials and personal information
- ☐ How the research results will be used
- ☐ Funding for the Research
- ☐ Conflicts of interest
- ☐ Any cost to the participants
- ☐ Potential advantages and disadvantages such as adverse events that may cause difficulties and how to handle them
- ☐ Conditions under which the research may be cancelled
- ☐ How to address questions and contact information

Year, Month, Date

Name (Signature)

Seal

The parents or guardians (If the person is a minor, or if there is anyone who lives together with him/her)

Name

Seal

Department of the person who provided the explanation

Name and Title of the person who provided the explanation (Signature) Seal

＊ （English version (actually using Japanese version)

For peer review only

同意書　（15 歳以上本人および保護者・代表同居人用）

帝京大学医療技術学部長殿

研究課題名：「整理整頓が苦手な若年者を対象の無作為化比較試験を用いた“片づけ・整理整頓教室”の効果に関する研究（くじ引きで教室をする群としない群を分け、教室の効果を見る研究）」

私は、この研究について説明者から文書により下記の項目について説明を受け、十分理解のうえ自由意思により本研究に参加することに同意します。

説明を受けた項目：

- □ 研究の目的・意義
- □ 研究の対象と方法
- □ 研究への自由意思参加・同意取消しの自由
- □ 研究の責任者・組織
- □ 研究の場所・期間
- □ 研究試料と情報の取り扱い
- □ 研究結果の扱い
- □ 研究資金源
- □ 利益相反
- □ 研究参加者の負担や支払いの有無
- □ 被る可能性のある個人の利益、不利益、人的トラブルを含む有害事象とその対応
- □ 研究中止の条件
- □ 質問への対応の仕方・連絡先

平成　　年　　月　　日

本人氏名（自署）

印

保護者・同居の代表者（未成年または同居の方がいらっしゃる場合）

氏名

印

説明者の所属・部署

説明者の職名・氏名（自署）

印

For peer review only - http://bmjopen.bmj.com/site/about/guidelines.xhtml

For peer review only

Consent Form

(For Individuals 15 years of age or older, Parents or Guardians)

Dear Department Head of Medical Technology of Teikyo University,

Research Title: Research on the effect of the "Decluttering and Organizing Class" using a random comparison experiment for young people who have difficulty in organizing their rooms (separating participants into groups of those with or without class sessions by drawing in order to monitor the effects)

I received a document containing the following information from a person who explained the research, and having fully understood it, agree to participate in the research voluntarily.

What was explained to me

- ☐ Purpose and meaning of the research
- ☐ Subjects and method of the research
- ☐ Voluntary participation and right to withdraw consent
- ☐ Personnel responsible for and organization of the research
- ☐ Location and timeframe of the research
- ☐ Handling of research materials and personal information
- ☐ Handling of research results
- ☐ Funding for the Research
- ☐ Conflicts of interest
- ☐ Cost and Payment for Participation
- ☐ Potential advantages and disadvantages such as adverse events that may cause difficulties and how to handle them
- ☐ Conditions under which the research may be cancelled
- ☐ How to address questions and contact information

Year, Month, Date

Name (Signature)

Seal

The parents or guardians (If the person is a minor, or if there is anyone who lives together with him/her)

Name

Seal

Department of the person who provided the explanation

Seal

＊ （English version (actually using Japanese version)

For peer review only
